# Rescue from Stx2-Producing *E. coli*-Associated Encephalopathy by Intravenous Injection of Muse Cells in NOD-SCID Mice

**DOI:** 10.1016/j.ymthe.2019.09.023

**Published:** 2019-10-01

**Authors:** Ryo Ozuru, Shohei Wakao, Takahiro Tsuji, Naoya Ohara, Takashi Matsuba, Muhammad Yunus Amuran, Junko Isobe, Morio Iino, Naoki Nishida, Sari Matsumoto, Kimiharu Iwadate, Noriko Konishi, Kaori Yasuda, Kosuke Tashiro, Misato Hida, Arisato Yadoiwa, Shinsuke Kato, Eijiro Yamashita, Sohkichi Matsumoto, Yoichi Kurozawa, Mari Dezawa, Jun Fujii

**Affiliations:** 1 Division of Bacteriology, Department of Microbiology and Immunology, Faculty of Medicine, Tottori University, Yonago 683-8503, Japan; 2 Department of Stem Cell Biology and Histology, Graduate School of Medicine, Tohoku University, Sendai 980-8575, Japan; 3 Department of Oral Microbiology, Okayama University Graduate School of Medicine, Dentistry and Pharmaceutical Sciences, Okayama 700-8525, Japan; 4 Department of Neurology, Hasanuddin University Faculty of Medicine, Makassar 90245, Indonesia; 5 Department of Bacteriology, Toyama Institute of Health, Imizu, Toyama 939-0363, Japan; 6 Division of Legal Medicine, Department of Social Medicine, Faculty of Medicine, Tottori University, Yonago 683-8503, Japan; 7 Department of Legal Medicine, Graduate School of Medicine and Pharmaceutical Sciences, University of Toyama, Toyama 930-0194, Japan; 8 Department of Forensic Medicine, The Jikei University School of Medicine, Nishi-shimbashi, Minato-ku, Tokyo 105-8461, Japan; 9 Department of Food Microbiology, Tokyo Metropolitan Institute of Public, Tokyo 169-0073, Japan; 10 Department of Bioscience and Biotechnology, Faculty of Agriculture, Kyushu University, Fukuoka 812-8581, Japan; 11 Division of Neuropathology, Department of Brain and Neuroscience, Faculty of Medicine, School of Medicine, Tottori University Faculty of Medicine, Yonago 683-8503, Japan; 12 Division of Clinical Radiology, Tottori University Hospital, Yonago 683-8504, Japan; 13 Department of Bacteriology, Niigata University School of Medicine, Niigata 951-8510, Japan; 14 Division of Health Administration and Promotion, Department of Social Medicine, Faculty of Medicine, Tottori University, Yonago 683-8503, Japan

**Keywords:** Shiga toxin-producing *Escherichia coli*, Muse cells, acute encephalopathy

## Abstract

Shiga toxin-producing *Escherichia coli* (STEC) causes hemorrhagic colitis, hemolytic uremic syndrome, and acute encephalopathies that may lead to sudden death or severe neurologic sequelae. Current treatments, including immunoglobulin G (IgG) immunoadsorption, plasma exchange, steroid pulse therapy, and the monoclonal antibody eculizumab, have limited effects against the severe neurologic sequelae. Multilineage-differentiating stress-enduring (Muse) cells are endogenous reparative non-tumorigenic stem cells that naturally reside in the body and are currently under clinical trials for regenerative medicine. When administered intravenously, Musecells accumulate to the damaged tissue, where they exert anti-inflammatory, anti-apoptotic, anti-fibrotic, and immunomodulatory effects, and replace damaged cells by differentiating into tissue-constituent cells. Here, severely immunocompromised non-obese diabetic/severe combined immunodeficiency (NOD-SCID) mice orally inoculated with 9 × 10^9^ colony-forming units of STEC O111 and treated 48 h later with intravenous injection of 5 × 10^4^ Muse cells exhibited 100% survival and no severe after-effects of infection. Suppression of granulocyte-colony-stimulating factor (G-CSF) by RNAi abolished the beneficial effects of Muse cells, leading to a 40% death and significant body weight loss, suggesting the involvement of G-CSF in the beneficial effects of Muse cells in STEC-infected mice. Thus, intravenous administration of Muse cells could be a candidate therapeutic approach for preventing fatal encephalopathy after STEC infection.

## Graphical Abstract

**Figure:**
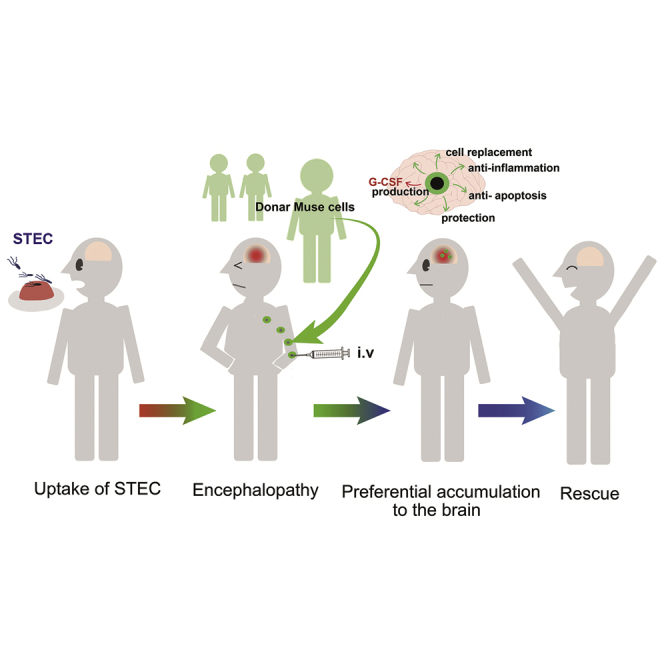

## Teaser

NOD-SCID mice orally inoculated with 9 × 10^9^ colony-forming units of Shiga toxin-producing *Escherichia coli* (STEC) O111 and treated 48 h later with an intravenous injection of 5 × 10^4^ human bone marrow-derived multilineage-differentiating stress-enduring (Muse) cells exhibited 100% survival. Thus, the intravenous administration of Muse cells might be a candidate therapeutic approach for preventing acute encephalopathy after STEC infection.

## Introduction

Shiga toxin-producing *Escherichia coli* (STEC) is a causative agent of hemorrhagic diarrhea, hemolytic uremic syndrome (HUS), and acute encephalopathies, which occasionally lead to sudden death.^1^ Infected individuals may develop serious neurologic complications, including apnea, seizures, coma, cortical blindness, hemiparesis, and loss of consciousness. Children who recover from HUS-related encephalopathies exhibit low IQ, poor academic achievement, and epilepsy.^1^ Current treatments for acute encephalopathy, including plasma exchange, steroid pulse therapy, immunoglobulin G (IgG) immunoadsorption, and the monoclonal C5 antibody eculizumab, have limited effects.^2^

The main Shiga toxins (Stxs) produced by STEC, Stx1a and Stx2a, comprise one A and five B subunit proteins.^3^ The Stxs-B subunit binds with high affinity to globotriaosylceramide Gb3 (CD77) on the plasma membrane of some eukaryotic cells,^4^ which is upregulated by lipopolysaccharide (LPS), tumor necrosis factor-α, and interleukin-1β.^5^, ^6^ The Stxs-B subunit is retrogradely transported from the cell membrane to the endoplasmic reticulum (ER), and only the Stxs-A subunit enters the cytosol.^7^ The Stxs-A subunit removes adenine-4324 in 28S RNA of the 60S ribosomal subunit by *N*-glycosidase activity,^8^ rendering ribosomes inactive for protein synthesis.^9^ Once Stxs translocate across the intestinal tract, they bind to neutrophils, monocytes, platelets, and other leukocytes circulating in the bloodstream and release microvesicles containing the toxins.^10^ Stxs target glomerular endothelial cells, which is the key event in HUS-related acute renal failure, and directly bind to neurons and endothelial cells in the CNS, leading to blood-brain barrier (BBB) damage and acute encephalopathy.^11^, ^12^, ^13^

We previously generated a mouse model of acute encephalopathy by oral inoculation with Stx2c-producing *E. coli* O157:H– (strain E32511).^11^ This model exhibits apoptosis associated with caspase-3 activation in neurons in the anterior horn of the spinal cord and the reticular formation of the medulla oblongata, as well as in brain microvascular endothelial cells.^12^ Signs of infection in our mouse model resemble features of human acute encephalopathy,^14^ such as tremor, paralysis of the lower extremities, and spinal defects.^12^ Intracerebroventricular administration of Stx2a induces reactive astrocytes with high expression of glial fibrillary acidic protein (GFAP) alongside apoptotic neurons in the anterior horn of the spinal cord, reticular formation of the medulla oblongata, and brain microvascular endothelial cells.^15^ Reactive astrocytes aggressively produce tumor necrosis factor-α and nitric oxide, and exhibit polymorphonuclear neutrophil chemoattractant activity,^16^ which affect the permeability and integrity of brain microvascular endothelial cells, thereby impairing BBB function.^17^

A novel non-tumorigenic endogenous pluripotent stem cell type, the multi-lineage differentiating stress-enduring (Muse) cell, was reported in 2010 by Kuroda et al.^18^ Muse cells are identified as cells positive for the pluripotency surface marker stage-specific embryonic antigen (SSEA)-3, and can be collected from the bone marrow, peripheral blood, and organ connective tissues. They are also available as several percent of cultured fibroblasts and mesenchymal stem cells (MSCs).^19^ They have low telomerase activity and are non-tumorigenic, consistent with the fact that they reside in normal adult tissues.^18^ Muse cells have several unique characteristics that might be beneficial for the treatment of STEC-induced acute encephalopathy. First, intravenously injected Muse cells specifically home to the site of damage mainly via sphingosine-1-phosphate signals that are produced by damaged cells and act through their receptors, which are expressed on Muse cells.^20^ Second, homed Muse cells exert anti-inflammatory, anti-apoptotic, anti-fibrotic, immunomodulatory, and paracrine protection effects, which are expected to be therapeutic for STEC-induced encephalopathy.^20^, ^21^, ^22^, ^23^, ^24^ They also replace damaged/apoptotic cells by spontaneous differentiation into tissue-constituent cells.^20^, ^21^, ^22^, ^23^, ^24^ Third, allografted and xenografted Muse cells escape host immunologic attack, successfully home to the damaged site, and remain in the tissue as tissue-constituent cells for longer than 6 months in allografts and ∼2 months in xenografts without need for immunosuppressants.^20^, ^23^ The ability of Muse cells to avoid host immunologic attack may be explained, at least in part, by their expression of histocompatibility leukocyte antigen G (HLA-G), a histocompatibility antigen that mediates immune tolerance.^25^ Fourth, Muse cells are easily accessible from commercially available MSCs and fibroblasts,^26^, ^27^ making them feasible for clinical application. Clinical trials using Muse cells to target four diseases, including stroke and spinal cord injury, were initiated in 2018.^25^ All of the clinical trials are based on intravenous injection of donor-derived Muse cells without HLA matching or long-term immunosuppressant treatment. Fifth, Muse cells tolerate stress by actively secreting prosurvival factors such as 14-3-3 proteins and serpin, which play a key role in regulating the cellular response to DNA damage.^28^, ^29^ Muse cells are thus expected to tolerate the microenvironment of STEC-induced encephalopathy, which contains strong stress factors derived from damaged neurons and glial cells, brain edema, inflammation, and impaired BBB function.

The ready availability of allogeneic Muse cells and the simplicity of intravenous administration are two important considerations for exploring the beneficial effects of Muse cells against STEC-induced encephalopathy. Here, we investigated the effectiveness of intravenous injection of human Muse cells on encephalopathy caused by oral STEC infection in mice and the main factors involved in the beneficial effects.

## Results

### Sensitivity of Crl:CD1 (ICR) and Non-obese Diabetic/Severe Combined Immunodeficiency (NOD-SCID) Mice to STEC O111 and O157

Following the protocol (Figure S1A), we found that among the four STEC strains, E32511 and O111 exhibited higher toxicity and were therefore selected for the present study. All of the mice infected with O157-Sakai and O157 Tokyo survived for 2 weeks, whereas 60% of ICR mice infected with O111 survived for 2 weeks. All of the mice infected with E32511 died within 144 h of infection (Figure S1B). Next, we investigated the minimum dose of E32511 and O111 required to kill all NOD-SCID mice. None of the NOD-SCID mice injected with 1 × 10^9^ colony-forming unit (CFU) of E32511 and O111 died (100% survival; data not shown), whereas all mice inoculated with 1 × 10^10^ CFU E32511 and O111 died within 108 h (Figure S1C). On the basis of these results, we used NOD-SCID mice inoculated with 1 × 10^10^ CFU of E32511 and/or O111 to investigate the effectiveness of treatment with human Muse cells.

### Distribution of Intravenously Injected Muse and Non-Muse Cells in E32511/O111-Infected NOD-SCID Mice

Human Muse cells were collected from human bone marrow-derived mesenchymal stem cells (BM-MSCs) as SSEA-3-positive cells by cell sorting, as previously described.^18^ Human “non-Muse cells,” i.e., cells other than Muse cells in the BM-MSCs, were collected as SSEA-3-negative cells.^18^ Previous studies demonstrated that intravenous administration of 5 × 10^4^ and 2 × 10^4^ human Muse cells into mouse liver damage^30^ and chronic kidney disease models,^23^ respectively, has therapeutic effects, and local administration of 3 × 10^4^ human Muse cells in a rat stroke model provides structural and functional recovery.^31^ Therefore, we considered 5 × 10^4^ Muse/non-Muse cells to be within the effective range for this experiment and injected this amount into infected NOD-SCID mice for the present investigation.

**Figure 1:**
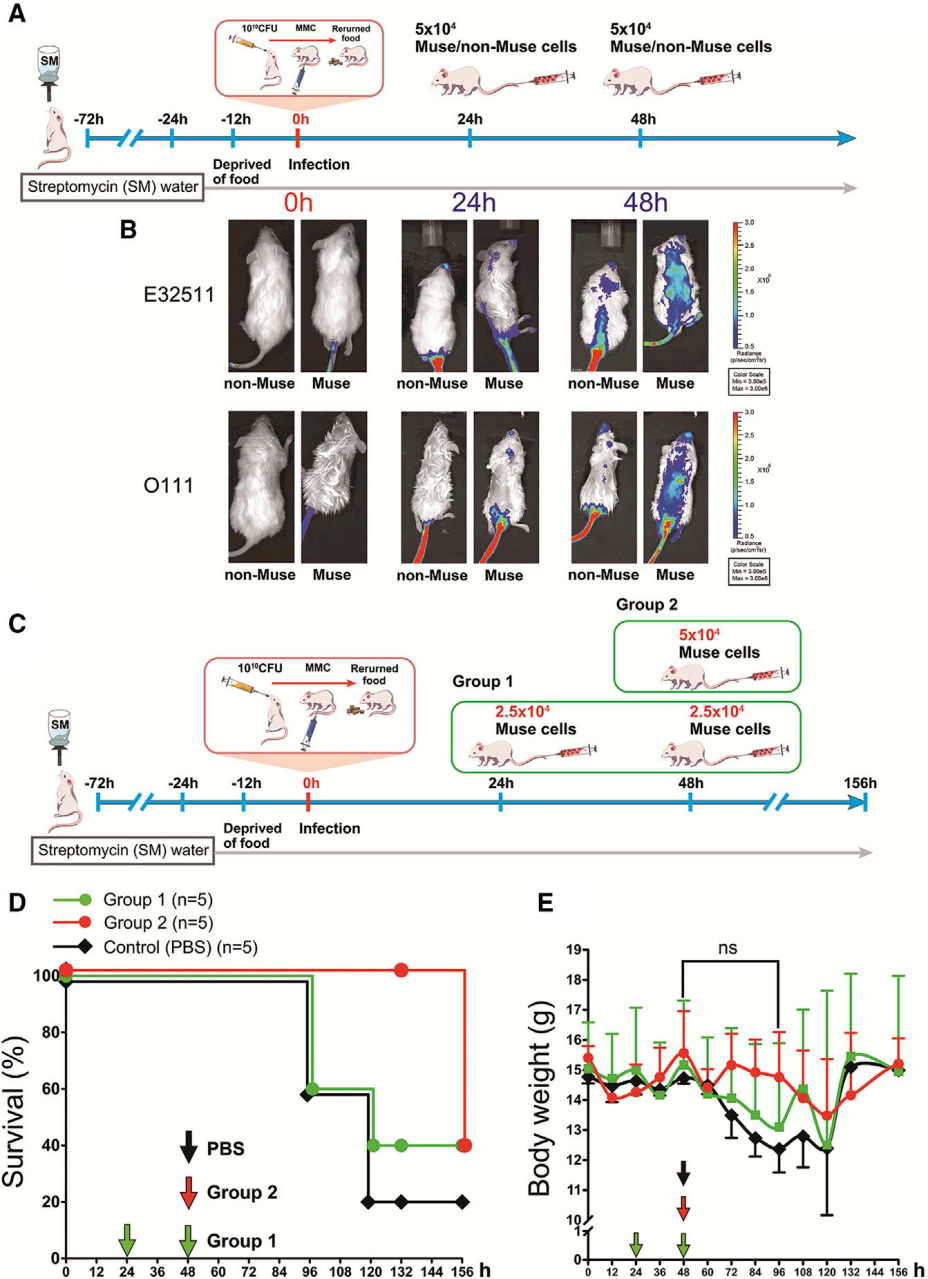
*In Vivo* Dynamics of Intravenously Injected Muse and Non-Muse Cells, and Evaluation of the Administration Method in E32511/O111-Infected NOD-SCID Mice (A) Experimental protocol for *in vivo* dynamics of Muse/non-Muse cells after intravenous injection. (B) Mice were orally inoculated with 1 × 10^10^ CFU of E32511 and O111. After 24 or 48 h, nano-Lantern-labeled Muse or non-Muse cells were intravenously injected into the tail vein. Mouse whole bodies were observed by an *in vivo* imaging system. (C) Experimental protocol for two groups of Muse cell injection. (D and E) Survival rate (D) and change in body weight (E) are shown. NOD-SCID mice infected with 1 × 10^10^ CFU of O111 were divided into two groups: one group (group 1) received an intravenous injection of 2.5 × 10^4^ Muse cells at two time points (24 and 48 h) and the other group (group 2) received an intravenous injection of 5 × 10^4^ Muse cells once at 48 h. Control animals received an intravenous injection of PBS at 48 h after O111 infection. Although the total number of Muse cells injected was the same in the two groups, all group 2 mice survived for 132 h (red line), whereas mice in group 1 (green line) and the PBS (black line) group began to die at 96 and 120 h with only 40% and 20% survival, respectively, at 132 h (D). The next day (156 h), however, the survival rate of mice in group 2 decreased to 40% (D). Body weight did not differ significantly between groups 1 and 2 at 48–96 h (E). Mice that survived in group 1 and the PBS group gained weight compared with mice in group 2 at 132 h, probably because the surviving mice in these groups were healthier. Of the five mice, only one mouse in the PBS group and two mice in group 1 survived at 132 h. These findings suggest that a single intravenous injection of 5 × 10^4^ Muse cells at 48 h was more effective than an intravenous injection of 2.5 × 10^4^ Muse cells twice at 24 and 48 h, even though the total injected Muse cell number was the same. ns, not significant.

The protocol for the *in vivo* imaging system (IVIS) used to evaluate the *in vivo* dynamics of intravenously administered Muse and non-Muse cells is shown in Figure 1A. Mice were orally inoculated with 1 × 10^10^ CFUof E32511 or O111. At 24 or 48 h after oral STEC inoculation, 5 × 10^4^ nano-Lantern-labeled human-Muse or non-Muse cells were injected into the tail vein and analyzed by the *in vivo* imaging system (Figure 1A). Both Muse and non-Muse cell injection at 24 h after infection produced almost no detectable luminescence except in the tails and heads of the E32511- and O111-infected mice, whereas injection of Muse cells at 48 h after infection produced detectable luminescence, particularly in regions corresponding to the brain and spinal cord in the E32511- and O111-infected mice. Injection of non-Muse cells into E32511- and O111-infected mice at 48 h produced less detectable signal (Figure 1B). Luminescence was lightly detected in the kidneys in the Muse group of E32511-infected mice injected at 48 h after infection (Figure 1B). These findings suggested that Muse cells administered intravenously at 48 h after infection distributed more broadly in the body than non-Muse cells administered at 24 h after infection.

We next compared the efficacy of one intravenous injection of 5 × 10^4^ Muse cells at 48 h after infection with that of two intravenous injections of 2.5 × 10^4^ cells, one at 24 h and one at 48 h, in O111-infected NOD-SCID mice (Figure 1C). On the basis of the survival rate up to 132 h after the O111 inoculation, one injection of 5 × 10^4^ Muse cells administered 48 h after infection was more effective than two divided injections of 2.5 × 10^4^ Muse cells administered at 24 and 48 h after infection (Figure 1D).

### Effect of Intravenously Injected Muse Cells on Survival and Body Weight in NOD-SCID Mice Infected with E32511 or O111

**Figure 2:**
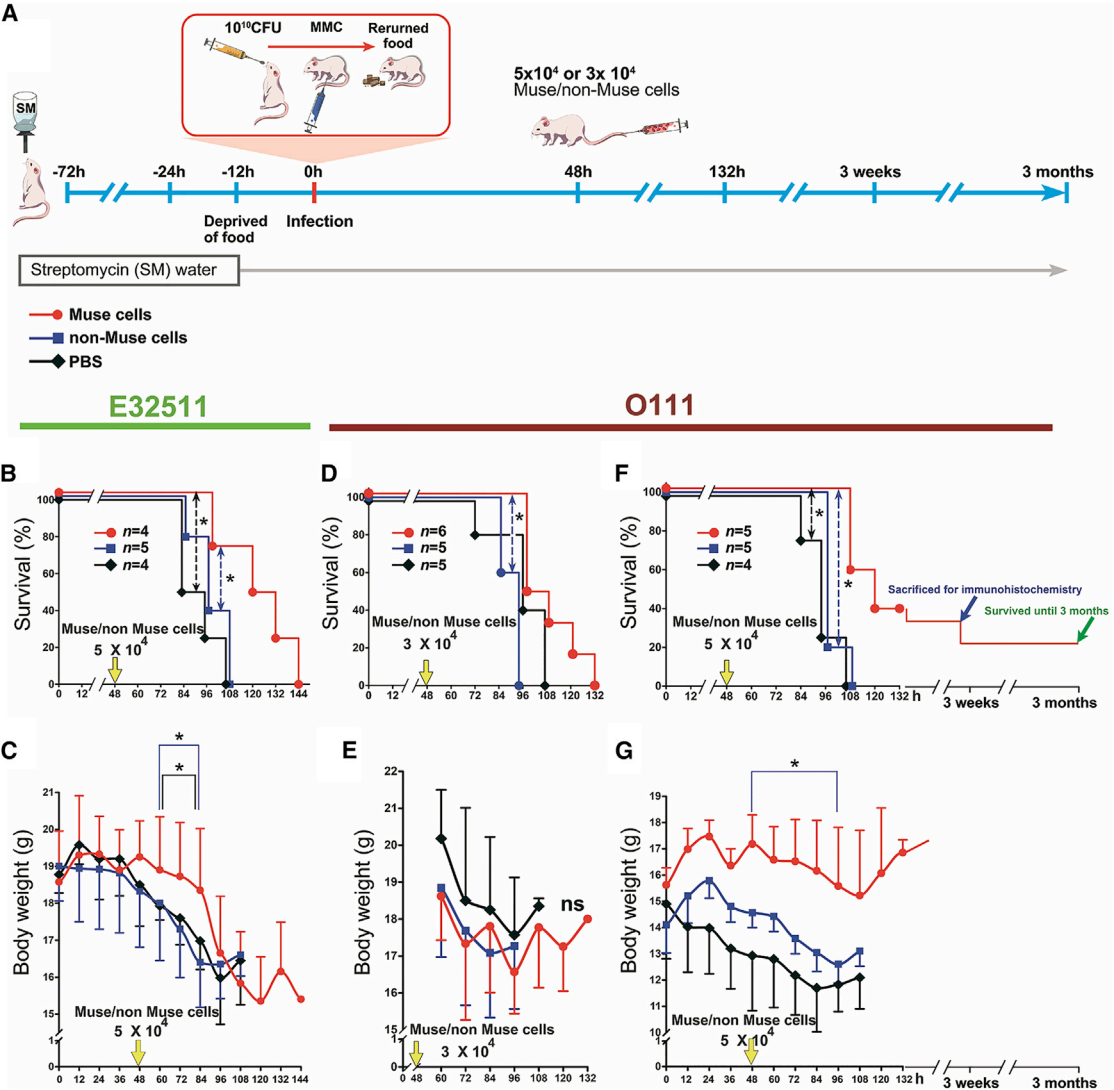
Effect of Intravenously Injected Muse Cells on Survival and Body Weight of NOD-SCID Mice Infected with E32511 or O111 (A) Experimental protocol for evaluating Muse/non-Muse cell injection on survival and change in body weight. (B and C) For E32511, 1 × 10^10^ CFU E32511 was orally inoculated, and survival rate (B) and body weight (C) were observed after intravenous injection of 5 × 10^4^ Muse/non-Muse cells. (D–G) For O111, 1 × 10^10^ CFU of O111 was orally inoculated, and survival rate (D and F) and body weight (E and G) were observed after intravenous injection of either 3 × 10^4^ or 5 × 10^4^ Muse/non-Muse cells, respectively. Mice in the control group were injected with the same volume of PBS. Statistical analysis was performed by the log rank test (Mantel-Cox) for survival curves or post hoc tests (Scheffe’s and Tukey’s) for the change in body weight. *p < 0.05. ns, not significant.

Based on the above findings, 5 × 10^4^ Muse/non-Muse cells were administered intravenously to NOD-SCID mice (∼14- to 18-g females) at 48 h after E32511 and O111 infection (Figure 2A). The control group was injected intravenously with the PBS in the same volume as that used for the Muse and non-Muse injections and at the same time point.

In E32511-infected NOD-SCID mice, survival was significantly longer in the Muse group compared with the non-Muse and control groups (p < 0.05; Figure 2B). A general linear model of repeated measures with Scheffe’s and Tukey’s post hoc tests revealed that body weight was significantly greater in the Muse group compared with the non-Muse and control groups until the first mouse died in the experiment, i.e., ∼60–84 h after infection (p < 0.05; Figure 2C).

In O111-infected NOD-SCID mice, dose dependency was evaluated by injecting 3 × 10^4^ or 5 × 10^4^ Muse/non-Muse cells. In O111-infected NOD-SCID mice injected with 3 × 10^4^ Muse cells, survival was increased compared with that in the non-Muse and control groups (p < 0.05; Figure 2D). Body weight, however, did not differ significantly among the three groups (Figure 2E). In O111-infected NOD-SCID mice injected with 5 × 10^4^ Muse cells, survival was 40% at 132 h, whereas survival in the non-Muse and control groups was 0% at 108 h (Figure 2F). Furthermore, body weight was significantly greater in the Muse group than in the other two groups from 48 to 96 h (p < 0.05; Figure 2G).

Notably, several mice in the Muse group injected with 5 × 10^4^ cells survived more than 132 h. Among the three repeated experiments shown in Figure 2F (n = 5/experiment), one-third of the total (5/15) mice survived for 3 weeks (Figure 2F, 3 weeks). Two mice were killed at 3 weeks for immunohistochemical analysis, and the remaining three mice were followed further. All of them survived for up to 3 months and were killed at this time point for immunohistochemical analysis (Figure 2F, 3 months).

### Distribution of Human Muse Cells in the Mouse Brain at 3 Weeks or 3 Months after the O111 Inoculation

We investigated the distribution of Muse cells in the brains of the mice surviving 3 weeks and 3 months. These long-term surviving mice exhibited no weight loss, spinal deformity, or flaccid paralysis of the extremities (data not shown).

**Figure 3:**
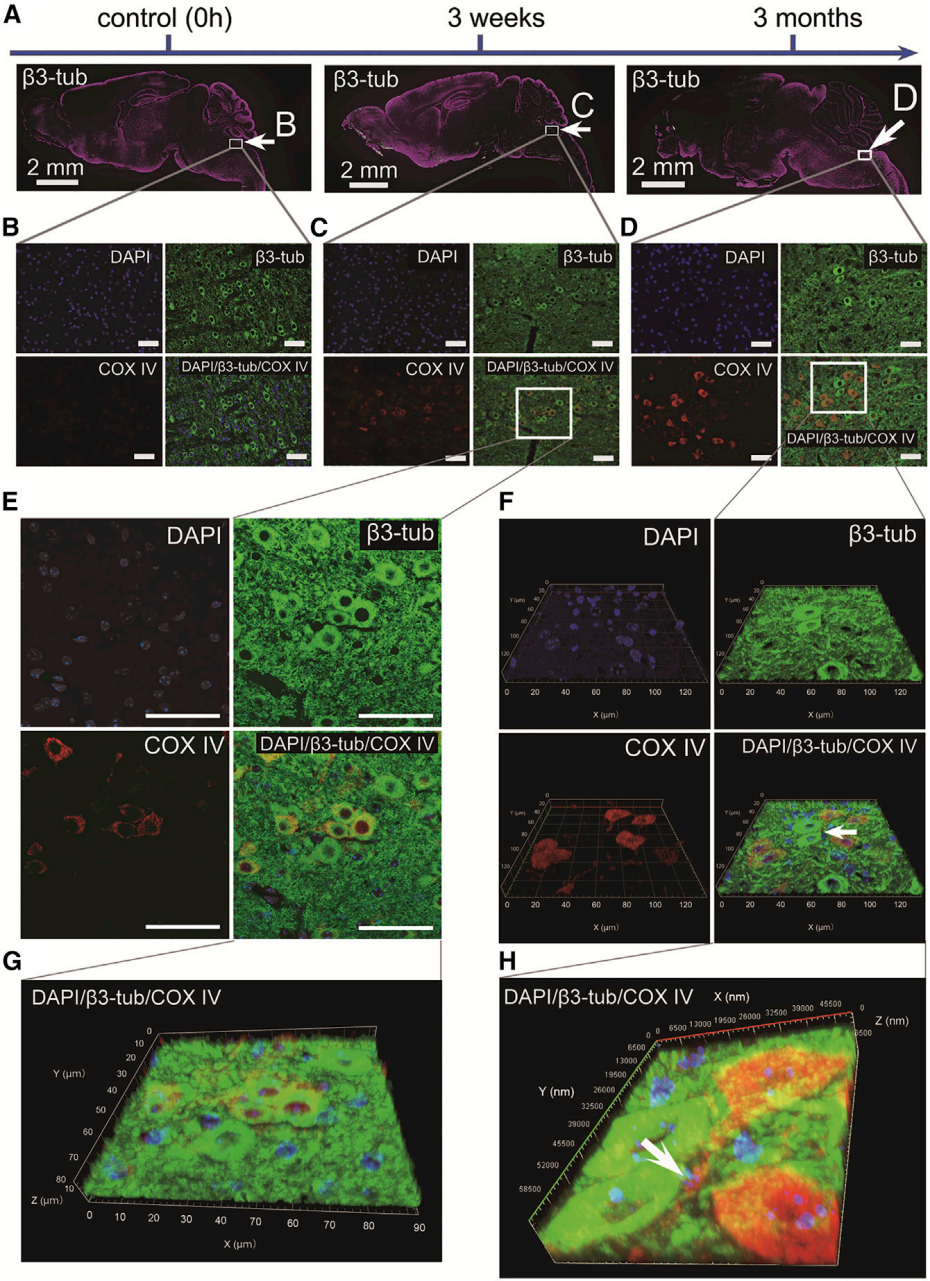
Distribution of Human Muse Cells in the O111-Inoculated Mouse Brain at 3 Weeks and 3 Months after Inoculation (A) Brains in the Muse group. The region indicated by the white rectangle indicates an area in which COX IV^+^-human Muse cells were detected. (B–D) Enlarged images of areas indicated by the white rectangles in (A). The distribution of human Muse cells, recognized as COX IV^+^ (red), and β3-tubulin (green), pan-specific to both human and mouse, is shown. (B) No COX IV immunoreactivity (engrafted white cells) was observed in the reticular formation of the medulla oblongata before injection of Muse cells as a control (0 h). (C) At 3 weeks after O111 infection, COX IV immunoreactivity was observed in mice injected with human Muse cells in the reticular formation of the medulla oblongata. (D) At 3 months after O111 infection, COX IV^+^ human Muse cells were detected in the reticular formation of the medulla oblongata. Scale bars: 50 μm. (E and F) High-magnification images of areas from (C) and (D), respectively. Especially, (F) is shown as 3D image to detect yellow signal, the overlap of red and green signals. Cells that were double positive for COX IV (red cells) and β3-tubulin (green cells) indicated neuronal marker expression in engrafted Muse cells. Scale bars: 50 μm. (G and H) 3D images of (E) and (F), respectively, are shown.

Because the Muse cells used in this study are human cells, they can be identified using the anti-human cytochrome *c* oxidase subunit 4 (COX IV) antibody, which specifically labels human cells, in mouse brain tissue. The brains of non-infected mice without any cell injection at 48 h were used as a negative control (0 h). Human Muse cells positive for COX IV (COX IV^+^ human Muse cells) were detected in the reticular formation of the medulla oblongata (Figures 3C and 3D). We also confirmed the distribution of Muse/non-Muse cells in E32511-infected NOD-SCID mouse brain using the anti-human mitochondria antibody with secondary anti-mouse IgG antibody conjugated with Alexa Fluor 488. Muse cells were integrated around the pons and medulla at 96 h, whereas non-Muse cells were scarcely detected in the brain (Figure S2C).

The majority of the COX IV^+^ human Muse cells did not express CD31, a marker for endothelial cells, or GFAP, a marker for astrocytes and reactive astrocytes (Figure S3). Muse cells expressing the neuronal cell marker β3-tubulin, however, were observed in mice that survived at 3 weeks and 3 months (Figures 3C–3G). These β3-tubulin^+^/COX IV^+^ cells exhibited a neuron-like morphology with nerve fiber-like processes in the 3-month-surviving mouse brain (Figure 3H).

Active caspase-3, an indicator of apoptosis, was evaluated in the E32511-infected NOD-SCID mouse brain. Active caspase-3 was detected at significantly higher rates in the medulla oblongata and pons in the non-Muse group than in the Muse group (p < 0.05; Figure S2D).

### GFAP Staining in the Brain of a STEC-Infected Patient

**Figure 4:**
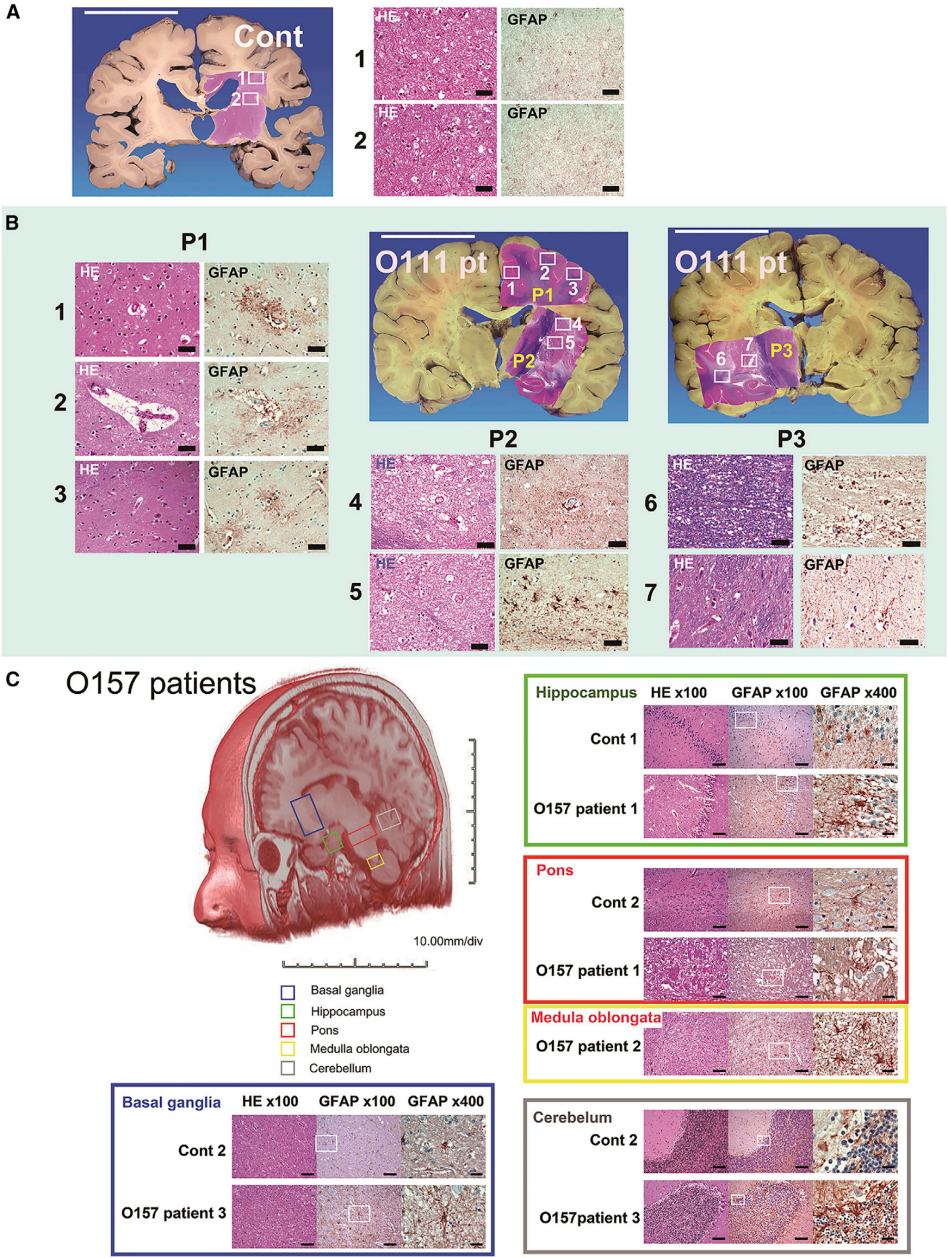
GFAP^+^-Reactive Astrocytes in the Patient Brain of Autopsy Cases Infected with O111 and O157 (A) An autopsy case as a control (43-year-old man). Scale bar: 5 cm. Left and right specimen panels show H&E staining and glial fibrillary acidic protein (GFAP) immunohistochemistry of paraffin-embedded sections (immunohistochemistry) for astrocyte staining, respectively (scale bar: 50 μm). (B) An autopsy case of an O111-infected patient (43-year-old woman). Scale bars: 5 cm. Areas were divided into three regions, P1, P2, and P3. From one to seven high-magnification images are shown for each white box in P1–P3. P1 includes the cerebrum tectorium. P2 and P3 include the basal ganglia, thalamus, hippocampus, and temporal lobe white matter. In each area, left and right specimen panels show H&E staining and GFAP immunohistochemistry, respectively (scale bars: 50 μm). (C) Orientation of human brain sagittal sections using MRI. The basal ganglia are shown in the blue box (O157 Tokyo patients), hippocampus in the green box, pons in the red box, medulla oblongata in the yellow box, and cerebellum in the gray box. Following the outbreak of STEC O157:H7 in 2016, autopsies were performed. In the basal ganglia, O157 patient 3 (90-year-old woman) was compared with control 2 (89-year-old woman). In the hippocampus, O157 patient 1 (82-year-old woman) was compared with control 1 (80-year-old woman). In the pons and medulla oblongata, O157 patient 2 (88-year-old woman) and O157 patient 3 (90-year-old woman) were compared with control 2 (89-year-old woman). In the cerebellum, O157 patient 3 (90-year-old woman) was compared with control 2 (89-year-old woman). Original magnification for H&E and GFAP: ×100, scale bars: 100 μm; original magnification for GFAP, ×400, scale bars: 25 μm.

Figures 4A and 4B show a case control (43-year-old man) and an autopsy case of a 43-year-old woman infected by O111, respectively. In the O111-infected brain, astrocytes detected by anti-GFAP were observed in the cerebrum with particularly strong reactivity around the vessels and in microvascular lesions (Figure 4B, P1, 1,2,3, GFAP). The reactive astrocytes became clasmatodendritic (Figure 4B, P2, 4, GFAP) with a complete loss of morphology and vacuolar degeneration in the thalamus (Figure 4B, P2, 4, H&E-stained sections). Clasmatodendritic was also observed in the cerebral white matter (Figure 4B, P3, 6, GFAP).

The brains of three autopsy cases from a STEC O157:H7 outbreak (82-, 88-, and 90-year-old females) were compared with those from normal age-matched control brains (80- and 89-year-old females). As shown in Figure 4C, more reactive astrocytes were detected in the basal ganglia, hippocampus, pons, medulla oblongata, and cerebellum of the infected samples compared with the normal tissues.

Similarly, reactive astrocytes were observed in the brain and upper spinal cord of O111- and E32511-infected NOD-SCID mice at 96 h after inoculation. This time point was selected because all of the inoculated mice died within 108 h (Figure S1C). Intense GFAP immunoreactivity, suggestive of reactive astrocytes, was identified in the medulla oblongata of O111-infected NOD-SCID mice and in the pons and spinal cord of E32511-infected mice (Figure S4).

### Engraftment of Intravenously Injected Muse Cells into O111-Infected NOD-SCID Mouse Brain Suppressed Reactive Astrogliosis

**Figure 5:**
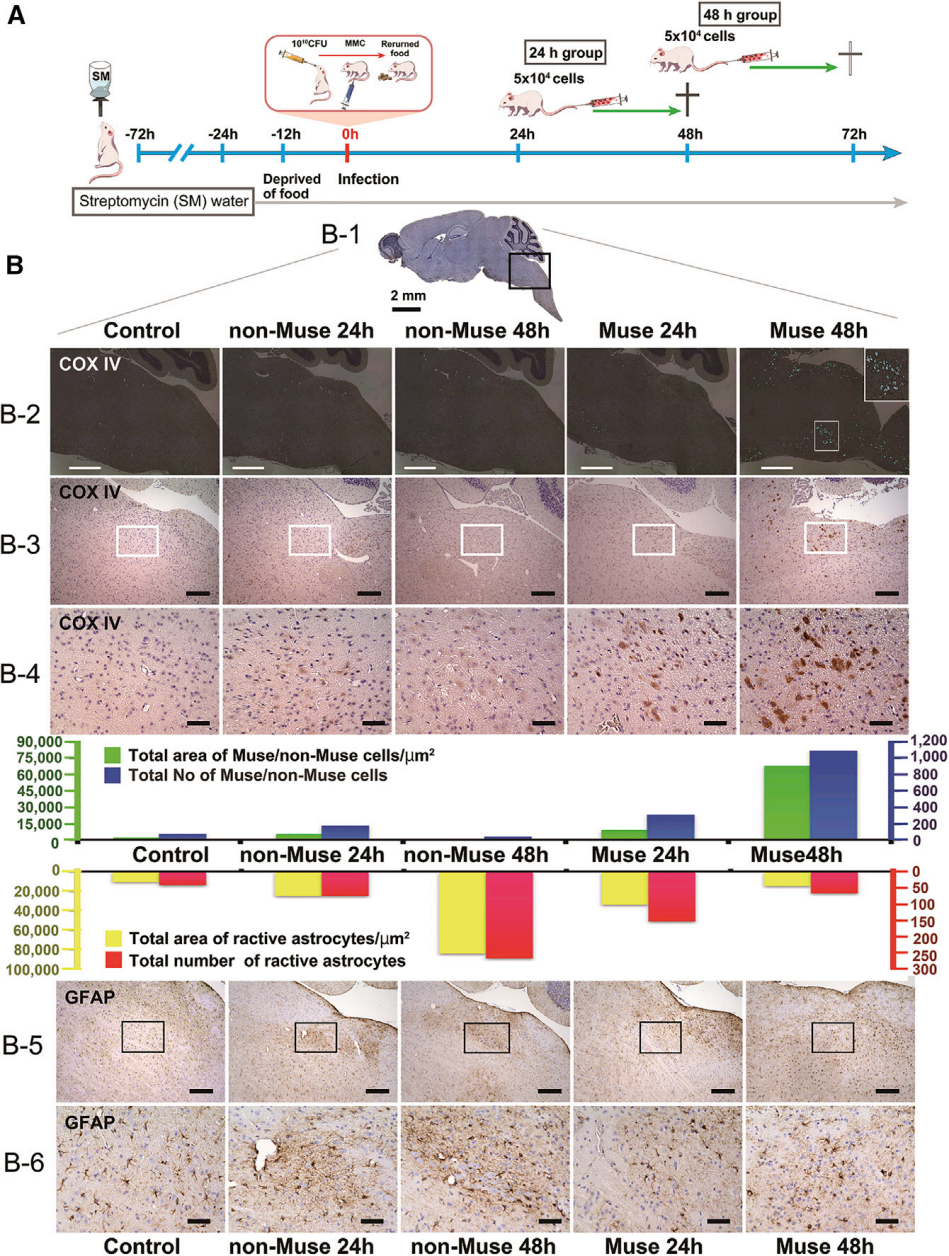
Engraftment of Intravenously Injected Muse Cells into O111-Infected NOD-SCID Mouse Brains and Rapid Suppression of Reactive Astrocytes (A) Experimental procedure. Mice were divided into two groups: 5 × 10^4^ Muse/non-Muse cells were intravenously injected into the mice at 24 (24-h group) and 48 h (48-h group) after inoculation with 1 × 10^10^ CFU of O111. Mice at 0 h after inoculation were used as the control. (B) Histologic analysis for detecting engrafted human Muse/non-Muse cells by human cytochrome *c* oxidase (COX IV) and reactive astrocytes by GFAP. Box shows the region analyzed, which corresponded to the reticular formation of the medulla oblongata. (B-2) Low-magnification images of the distribution of Muse and non-Muse cells in each group. Injected cells are indicated by the light blue signal detected by immunohistochemistry with tyramide signal amplification (mIHC) plus cyanine 3/fluorescein-second antibody conjugate with BZ analyzer. Inset in the Muse 48 h is a high-magnification image of the area indicated by the white box. (B-3) Low-magnification image of COX IV^+^ cells in the reticular formation of the medulla oblongata, and white boxes in B-3 are enlarged in B-4. (B-5) Low-magnification image of GFAP^+^ cells in the reticular formation of the medulla oblongata, and black boxes in (B-5) are enlarged in (B-6). Scale bars: 200 μm (B-2, B-3, and B-5); 50 μm (B-4 and B-6). Graphs of total area of Muse/non-Muse cells/μm^2^ (green bar), total number of Muse/non-Muse cells (blue bar), total area of GFAP^+^ reactive astrocyte/μm^2^ (red bar), and total number of GFAP^+^ reactive astrocytes (yellow bar) are shown.

We next assessed the effectiveness of intravenous injection of human Muse cells into NOD-SCID mice infected with O111. The schema of experiments is shown in Figures 5A and 5B. Mice were orally inoculated with O111 and intravenously injected with 5 × 10^4^ human Muse/non-Muse cells at either 24 or 48 h after inoculation. Mice were killed the day after the injection, and the whole brain was cut into sagittal sections for immunohistochemistry. Human COX IV^+^-Muse cells were identified in the reticular formation of the medulla oblongata in both the 24- and 48-h groups (Figure 5B, B-4). Total area (green bars) and number of engrafted cells (blue bars) were higher in mice injected with Muse cells than in those injected with non-Muse cells, at both 24 and 48 h (Figure 5B, green and blue colored graph). The total area (yellow bars) and number (red bars) of GFAP-positive cells did not differ between the Muse and non-Muse groups at 24 h, but at 48 h, the Muse group had fewer GFAP^+^ cells than the non-Muse group (Figure 5B, yellow and red colored graph). These findings suggested that Muse cells injected intravenously 48 h after O111 infection engrafted to the encephalitic brain and rapidly suppressed reactive astrocytes. A similar tendency was observed in coronal brain sections of mice inoculated with E32511 (Figure S5).

### Effects of Muse Cells on Stx2a/LPS-Treated Astrocytes *In Vitro*

**Figure 6:**
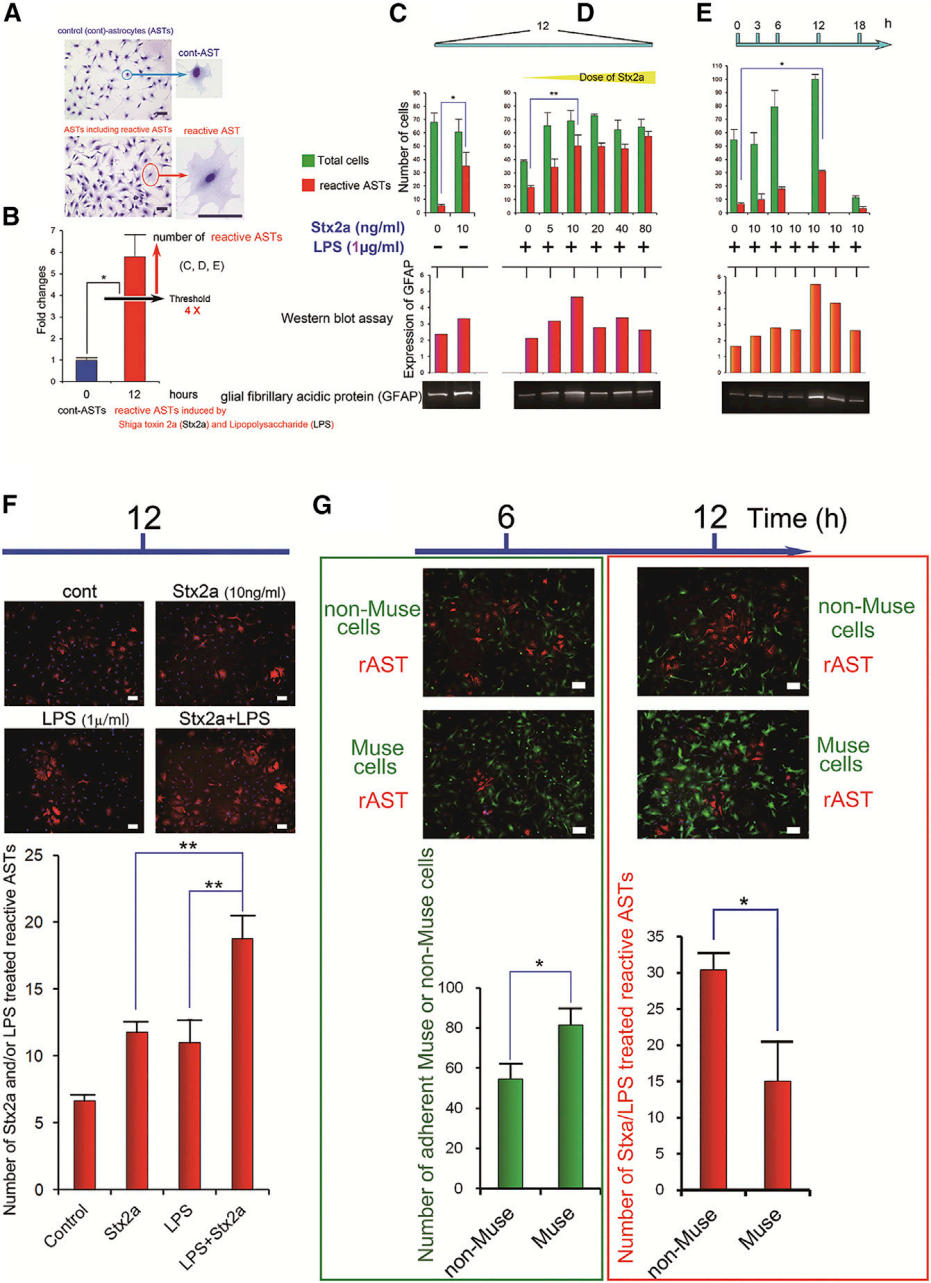
Effects of Muse Cells on Stx2a/LPS-Treated Reactive Astrocytes *In Vitro* (A) May-Grunwald-Giemsa staining of control and Stx2a/LPS-treated reactive astrocytes (rASTs) induced by Shiga toxin 2 (Stx2a) and lipopolysaccharides (LPSs). Scale bars: 50 μm. (B) Images of Stx2a/LPS-treated astrocytes in (A) were analyzed with ImageJ software to calculate the cell area. The threshold for Stx2a/LPS-treated reactive ASTs was set four times larger than that of control astrocytes. (C and D) Stx2a dose response in total astrocytes (green bar) and Stx2a/LPS-treated reactive ASTs (red bar) treated with (C) 0 or (D) 1 μg/ml LPS for 12 h. (E) Stx2a (10 ng/mL) increased the total number of astrocytes and Stx2a/LPS-treated reactive ASTs in a time-dependent manner. Similarly, Stx2a dose and time responses were observed (red bar) by western blot assay. (F) GFAP^+^ astrocytes treated with Stx2a (10 ng/mL) and/or LPS (1 μg/mL). The graph shows that Stx2a (10 ng/mL) plus LPS (1 μg/mL) induced significant upregulation of GFAP in astrocytes at 12 h after incubation compared with Stx2a or LPS alone. **p < 0.01. (G) Co-culture of GFP-labeled Muse or non-Muse cells (both green cells) and Stx2a/LPS-induced reactive ASTs (red cells; 6 h after addition of Stx2a and LPS). Significantly more Muse cells than non-Muse cells adhered to the culture dish after 6 h. *p < 0.05. Co-culture of GFP-labeled Muse cells and Stx2a/LPS-reactive astrocytes for 12 h induced a substantial decrease in reactive ASTs. *p < 0.05. Scale bars: 50 μm (inset).

May-Grunwald-Giemsa staining revealed that reactive astrocytes treated with Stx2a/LPS have a more diffuse cytoplasm than non-treated astrocytes (control), as shown in Figure 6A. Therefore, in this experiment, the criterion for Stx2a/LPS-treated reactive astrocytes was a 4-fold larger size than control astrocytes (Figure 6B). There was no difference in detected reactive astrocyte between the 4-fold larger sized astrocytes and GFAP immunostaining reactive astrocytes (Figure S6). In each chamber, the number of Stx2a/LPS-treated reactive astrocytes was counted in four fields under a 40× objective, and the mean of three samples was calculated. Combined treatment with 10 ng/mL Stx2a and 1 μg/mL LPS for 12 h significantly upregulated the number of GFAP^+^ reactive astrocytes, as confirmed by western blot (Figures 6C–6E). Immunohistochemical analysis revealed that combined treatment with 10 ng/mL Stx2a and 1 μg/mL LPS for 12 h significantly upregulated the number of GFAP^+^ astrocytes compared with treatment with either Stx2a or LPS alone (Figure 6F). In addition, treatment with either Stx2a or LPS alone increased the number of GFAP^+^ reactive astrocytes, but the number was not as high as when treated with the combination of Stx2a and LPS (Figure 6F). To assess the effect of Muse and non-Muse cells, astrocytes were cultured overnight in astrocyte basal medium, and then GFP-labeled Muse or non-Muse cells were added to astrocytes with 10 ng/mL Stx2a and 1 μg/mL LPS for co-culture. A greater number of Muse cells attached to the culture dish compared with non-Muse cells at 6 h (Figure 6G), and a smaller number of GFAP^+^ astrocytes was observed in the Muse cell co-culture compared with the non-Muse cell co-culture in the presence of Stx2a and LPS at 12h (Figure 6G). These findings suggested that reactive astrocytes were more effectively suppressed by Muse cells than by non-Muse cells.

### Microarray Analysis of Human Muse and Non-Muse Cells

**Figure 7:**
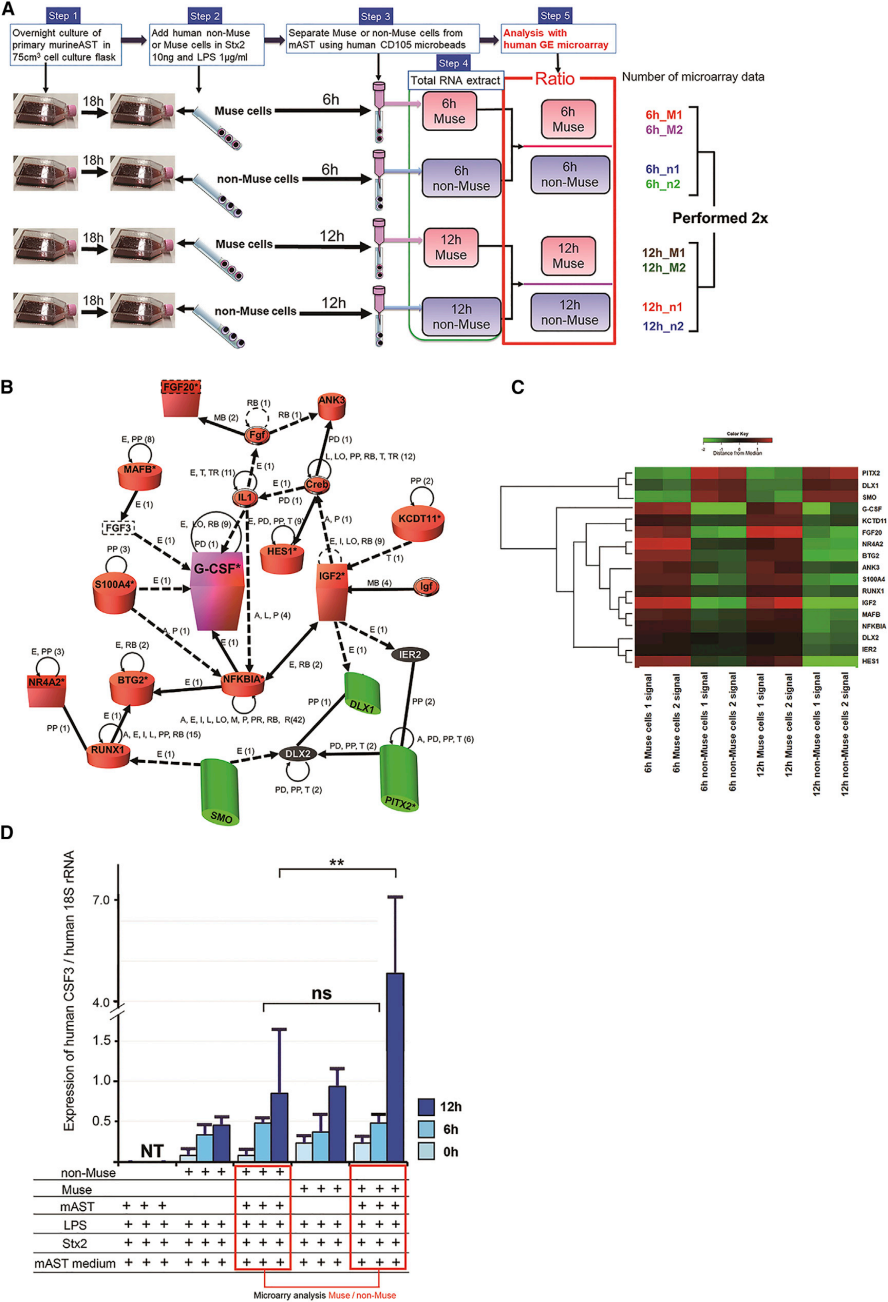
Microarray Analysis (A) Protocol for microarray analysis. (B) Pathway determination by ingenuity pathway analysis of microarray data in comparison 1 (6h_M1 to 6h_n1 in the colored rightmost number of the protocol). (C) The heatmap data of comparison 1. (D) Results of digital PCR analyses with TaqMan gene expression assays for G-CSF expression in reactive astrocytes either with or without co-culture with Muse and non-Muse cells. NT; no tested, ns; not significant. **p < 0.01.

We performed microarray analysis to assess the effect of Muse/non-Muse cells on Stx2a/LPS-treated reactive astrocytes (Figure 7A). The microarray analysis was repeated twice. We evaluated the expression of approximately 1,000 of 49,178 probes. To identify upregulated or downregulated genes between control and experimental samples, we calculated the *Z* score [*Z*] and ratio (non-log-scaled fold change) from the normalized signal intensities of each probe, and established criteria for regulated genes: *Z* score ≥ 2.0 and ratio ≥ 1.5-fold (upregulated genes), and *Z* score ≤ −2.0 and ratio ≤ 0.66-fold (downregulated genes) (the ratio of Muse and non-Muse cells at 6 h [Table S1] and 12 h [Table S2] after adding Stx2a and LPS). In the detailed microarray protocol (Figure 7A, the colored rightmost number), comparison 1, comparison 2, comparison 3, and comparison 4 indicated a ratio of 6h_M1 to 6h_n1, 6h_ M1 to 6h_n2, 6h_M2 to 6h_n1, and 6h_M2 to 6h_n2, respectively (Table S1). Comparison 5, comparison 6, comparison 7, and comparison 8 indicated a ratio of 12h_M1 to 12h_n1, 12h_M1 to 12h_n2, 12h_M2 to 12h_n1, and 12h_M2 to 12h_n2, respectively (Table S2). Genes focused on the keyword “neuronal differentiation” were predominantly identified through ingenuity pathway analysis (IPA). In particular, the granulocyte-colony-stimulating factor (G-CSF) gene was significantly upregulated more than 14.5-fold in response to Stx2a/LPS treatment (Figure 7B; comparison 1 in Table S1). Fibroblast growth factor-20, insulin-like growth factor-2, and other genes were reduced approximately 10-fold in the presence of reactive astrocytes (Figure 7B; comparison 1 in Table S1). The heatmap is shown in Figure 7C and in comparison 1 in Table S1. The microarray results were verified by digital PCR analyses with TaqMan gene expression assays using eukaryotic 18S rRNA as an endogenous control. Significantly increased G-CSF mRNA expression was detected in Muse cells co-cultured with Stx2a/LPS-treated reactive astrocytes for 12 h compared with non-Muse cells cultured in the same manner (p < 0.05; Figure 7D).

### Muse Cell Rescue Effects in O111-Infected NOD-SCID Mice Are Mediated in Part by G-CSF

**Figure 8:**
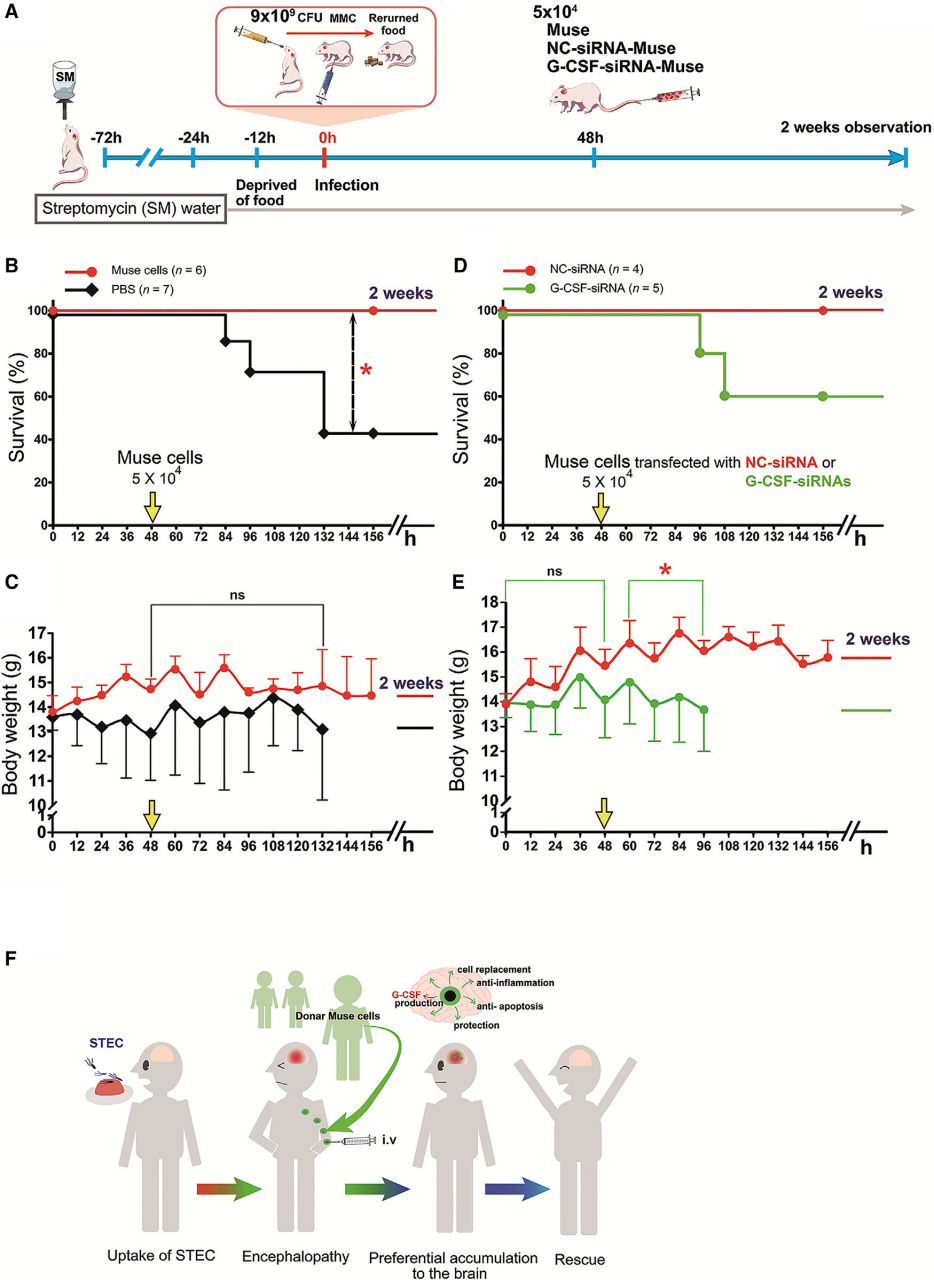
G-CSF siRNA Abolished the Increased Survival Effect of Muse Cells in O111-Infected NOD-SCID Mice (A) Experimental protocol. (B) The O111-infected NOD/SCID mice showed 100% survival (red line). The control group received an intravenous injection of PBS in the same volume as the Muse group (black line). (C) Change in body weight in the Muse and control groups after O111 infection. Statistical analysis was performed by the log rank test (Mantel-Cox) for survival curves or post hoc tests (Scheffe’s and Tukey’s) for the change in body weight. (D) Survival rate following intravenous injection of G-CSF-siRNA-introduced Muse cells (green line) and NC (Control)-siRNA-introduced Muse cells (red line). Statistical analysis was performed using the log rank test (Mantel-Cox). (E) Change in body weight after intravenous administration of G-CSF-siRNA-Muse cells (green line) and NC-siRNA-Muse cells (red line). Statistical analysis was performed by post hoc tests (Scheffe’s and Tukey’s). (F) Schematic of Muse cell treatment. Patients with STEC-associated encephalopathy were treated with donor-derived Muse cells by intravenous injection without HLA matching and long-term immunosuppressant use. Muse cells that homed into the brain exert pleiotropic effects including anti-inflammation, immunomodulation, anti-apoptosis, G-CSF production, protection effect, and cell replacement to rescue patients. *p < 0.05. ns, not significant.

To clarify the role of G-CSF in the Muse cell effects, we introduced G-CSF (identical to colony stimulation factor 3 [CSF3])-small interference RNA (siRNA) into Muse cells that were then intravenously injected into O111-infected NOD-SCID mice. We first developed a 100% surviving NOD-SCID mouse model by orally inoculating O111 at a dose of 9 × 10^9^ CFU, and then 5 × 10^4^ Muse cells were intravenously administered at 48 h after infection (Figure 8A). All NOD-SCID mice (100%) with intravenous administration of 5 × 10^4^ Muse cells survived for more than 2 weeks after O111 infection (Figure 8B). The survival rate in the control group injected with PBS was significantly reduced at 2 weeks (43%, p < 0.05; Figure 8B). Nonetheless, the body weights of mice in the control group were not significantly lower than those of mice in the Muse group from 48 to 132 h (Figure 8C).

Next, we used three kinds of siRNA to interfere with the G-CSF expression (Silencer Select Thermo Fisher Scientific Ink siRNA: s3606, s3607, and s3608). Compared with Muse cells transfected with non-targeting control-siRNA (NC-siRNA), siRNA s3607 reduced G-CSF transcript levels by 51% in Muse cells, which was the highest suppression rate among the three siRNAs (Figure S7A). Furthermore, 10 nM siRNA s3607 reduced the expression of G-CSF in Muse cells to 21% that of controls (Figure S7B). We then investigated the effect of G-CSF suppression on the survival rate and change in body weight. Notably, 40% of mice treated with G-CSF siRNA-containing Muse cells died and 60% survived. None of the mice treated with NC-siRNA Muse cells died within 2 weeks (Figure 8D). Body weight was significantly reduced from ∼60 to 96 h in mice treated with CSF3-siRNA-transfected Muse cells compared with mice treated with NC-siRNA-transfected Muse cells (Figure 8E). These findings suggested that G-CSF contributes to the survival effect of Muse cells in O111-infected NOD-SCID mice.

## Discussion

The beneficial effects of Muse cells on STEC-associated acute encephalopathy were demonstrated by several key findings. First, intravenous administration of only 5 × 10^4^ human Muse cells effectively prolonged survival of mice infected with a lethal dose of STEC (1 × 10^10^ CFU of O111 or E32511). Second, some mice in the Muse group survived for up to 3 months. Histologic analysis of these mice revealed the integration of intravenously administered human Muse cells having a neuron-like morphology in the brain. Third, Muse cell injection decreased the number of GFAP^+^ reactive astrocytes and suppressed caspase-3 activation in the brain. Muse cells have been applied to clinical trials for acute myocardial infarction, stroke, epidermolysis bullosa, and spinal cord injury by intravenous injection of donor-derived Muse cells without HLA-matching and immunosuppressant treatment.^25^ The same strategy is expected to be successful for emergency treatment of STEC-associated acute encephalopathy (Figure 8F).

Reports of human autopsy cases obtained during the outbreak of STEC O111 in Japan indicated a relationship between reactive astrocytes and brain edema and hernia induced by Stx2a-producing STEC O111:H8, which was believed to be the cause of death.^32^ In this study, we confirmed the presence of reactive astrocytes in autopsy cases of STEC-infected patients. Thus, controlling reactive astrocytes is a critical goal in treating STEC-associated acute encephalopathy. O111- and E32511-infected NOD-SCID mice also exhibited reactive astrocytes in the medulla oblongata, pons, and upper spinal cord. In a previous study, we demonstrated high-intensity areas in the reticular formation of the medulla oblongata in the O111-infected mouse model using *in situ* hybridization,^12^ consistent with another study reporting the same finding by MRI in a rabbit model of Stx2a toxicity, suggesting that STEC infection affects the medulla oblongata.^33^ In the present study, we detected Muse cells in this area, but very few non-Muse cells, and mice treated with Muse cells exhibited reduced caspase 3-positivity.

Steroid pulse therapy might have a protective effect against BBB impairment induced by Stx2a. Importantly, however, β-methasone sodium phosphate-steroid pulse therapy has limited protective effects against brain edema induced by intravenous injection of Stx1a.^34^ Glucocorticoid dexamethasone protects neurons and decreases astrogliosis in mice administered a sublethal dose of Stx2a or Stx2a plus LPS.^12^, ^35^ Stxs have *N*-glycosidase activity that depurates ribosomes and stops protein synthesis in the translation elongation phase.^8^ The resulting ribotoxic stress response leads to activation of the p38 mitogen-activated protein kinase pathway^36^ and the expression of cytokines such as interleukin-1β and interleukin-8 in macrophage-like cells.^37^ Boccoli et al.^15^ reported that intracerebroventricular administration of Stx2a increases astrogliosis in rats. In the brain, aquaporin-4 (AQP4) is localized to the perivascular endfeet of astrocytes, and an increase in GFAP immunoreactivity in peri-vasculature astrocytes is associated with downregulation of AQP4 expression in rats injected with culture supernatants of *E. coli*-expressing Stx2a.^38^ A sublethal dose of Stx2a causes BBB disruption and astrocytic edema in mice.^39^ BBB impairment accompanied by a downregulation of AQP4 expression is associated with reactive astrogliosis induced by Stx2a.^38^ In the present study, Muse cells decreased the number of reactive astrocytes *in vivo* and *in vitro*, thereby contributing to protect and maintain BBB integrity. A recent study showed that neuroinflammation and ischemia induce two different types of reactive astrocytes, A1 and A2. Liddelow et al.^40^ reported that activated microglia induce A1 astrocytes and kill axotomized neurons by becoming neurotoxic. On the other hand, reactive astrocytes with strong GFAP immunoreactivity were observed even without active microglia in the reticular formation of the medulla oblongata, and neurons around reactive astrocytes appeared to be degenerating with vacuolar changes.^12^ It is unclear whether or not the E32511-induced reactive astrocytes in our study were A1 astrocytes.

Neurologic impairment, the most life-threatening acute complication of STEC infection, develops in approximately 20%–25% of patients with STEC-induced HUS and may lead to sudden death or severe neurologic complications, including lethargy, apnea, coma, seizures, cortical blindness, and hemiparesis.^41^ We previously reported that sudden death associated with fatal encephalopathy after HUS is significantly associated with bloody diarrhea, a complete bedridden status, and a high white blood cell count.^42^ Even HUS-induced encephalopathy patients who escape sudden death, however, may still suffer the after-effects of infection, i.e., a low IQ, behavioral difficulties, low verbal abilities, and lower academic achievement, particularly in children.^43^ Following the German outbreak of STEC O104, severe neurologic complications, including delirium, myoclonus, aphasia, seizure, coma, and respiratory failure, were reported in adult patients with HUS, and many of them continue to suffer from hemiparesis, epilepsy, seizure, aphasia, and visual disturbances.^44^ A range of treatments have undergone clinical trials, including IgG immunoadsorption,^45^ plasma exchange, and the monoclonal antibody against complement 5 (C5) eculizumab. In the present study, validation of these treatments was not performed because NOD-SCID mice have no IgG or complement activity. We found that a single injection of as few as 5 × 10^4^ Muse cells at 48 h after O111 infection had beneficial effects and prolonged survival of mice with fatal encephalopathy without the associated neuronal damage, including spinal deformity and paralysis. Future studies are needed to optimize the Muse cell dosage.

Stxs-induced *N*-glycosidase activity inhibits protein synthesis,^8^ but it is unknown whether Stx-induced apoptosis or necrosis of cells occurs after binding to the surface of Gb3. Stx1-induced apoptosis in HeLa cells within 4 h requires the presence of caspase-6, -8, and -9, followed by activation of caspase-3, leading to nuclear DNA fragmentation.^46^ The initial cause of the apoptotic pathway induction, however, remains unknown. We focused on the ER stress response, a well-known trigger of apoptosis, and clarified that protein kinase R-like ER kinase, eukaryotic initiation factor-2α, and activated transcription factor-6 signaling pathways play an essential role in the expression of C/EBP homologous protein, a cellular stress sensor, during ER stress.^47^ Stx2a-induced apoptosis is mediated by ER stress involving C/EBP homologous protein in human brain microvascular endothelial cells, which leads to apoptosis mediated through intrinsic and extrinsic apoptosis pathways.^48^ Using microarray analysis, we identified G-CSF as an important factor in the effect of Muse cells on O111-associated mouse acute encephalopathy. G-CSF is a 19.6-kDa glycoprotein used for the treatment of neutropenia.^49^ It is a hematopoietic growth factor produced by monocytes, mesothelial cells, fibroblasts, and neurons that promotes the survival of mature neutrophils.^50^ G-CSF treatment is associated with significant improvement of the left ventricular ejection fraction in patients with acute myocardial infarction^51^ and effectively suppresses ER stress in a model of middle cerebral artery occlusion.^52^ Notably, knockdown of G-CSF in Muse cells attenuated their effects on survival in O111-infected mice. Body weight was also significantly reduced in mice treated with the G-CSF knockdown Muse cells. Furthermore, in the 100% surviving NOD-SCID mouse model established by oral inoculation of O111, intravenous administration of 5 × 10^4^ Muse cells at 48 h after infection led to 100% survival compared with 43% survival of the control group injected with PBS. The effect of Muse cells may be partially mediated by G-CSF production, which maintains AQP4 integrity and vascular endothelial growth factor induction, leading to improved neurologic functions by reducing brain edema, BBB permeability, neuronal death, and apoptosis.^53^ G-CSF also stimulates proliferation of neuronal/glial progenitor cells, improves neurocognitive function, and restores cerebral white matter damage from irradiation injury.^54^

This study has some limitations. The use of NOD-SCID mice allowed the use of human Muse cells to distinguish exogenous Muse cells from endogenous Muse cells, but limits this study to evaluating the ability of Muse cells to protect against STEC in immunodeficient mice. Future studies using mouse Muse cells in immunocompetent mice should be performed. In addition, the study examined a limited number of doses of Muse cells at only two time points. Further studies to determine the optimal dosing should be performed. Lastly, small numbers of mice were used for the survival analyses. Larger numbers would allow for analysis of the dose that kills 50% of the mice and more quantitative analysis.

Many STEC patients with severe neuronal complications develop epilepsy and intellectual disability. The anti-inflammatory and anti-apoptotic effects of Muse cells may synergistically increase the probability of a good outcome for patients with neurologic involvement associated with STEC infection. Further *in vitro* and *in vivo* studies are required to clarify the mechanisms underlying the survival effects of Muse cells in STEC infection.

## Materials and Methods

### Ethics Statement

Animal experiments were carried out in strict accordance with the recommendations in the Guidelines for the Proper Conduct of Animal Experiments formulated by the Science Council of Japan. The protocol was approved by the Committee on the Ethics of Animal Experiments of Kyushu University (Permit Number: A23-141-1) and Tottori University (Permit Number: 14-Y-17). All surgeries were performed under sevoflurane anesthesia, and all efforts were made to minimize suffering.

### Animals

Female ICR and NOD-SCID mice (∼14–18 g) were purchased from Charles River Laboratories Japan (Yokohama, Japan). NOD-SCID mice are characterized by an absence of functional T and B cells, lymphopenia, hypogammaglobulinemia, normal antigen-presenting cells, myeloid cells, and natural killer cells. The mice also have no detectable IgM, IgG1, IgG2a, IgG2b, or IgG3. Therefore, NOD-SCID mice (https://www.jax.org/strain/001303) could accept human Muse or non-Muse cells for cell transfer experiments in contrast with using nude mice. Mice were housed in cages with a 12-h light-dark cycle and were provided with free access to food and distilled water.

### Bacterial Strains and Streptomycin- and Mitomycin C-Resistant STEC

We used the clinically isolated strain of Stx2-producing STEC O111:H8 (STEC O111) from the Toyama prefecture outbreak. The Stx2c-producing *E. coli* O157:H- strain E32511/HSC (STEC O157) was a kind gift from Prof. Mohamed A. Karmali (McGill University, Québec, Canada). The Stx1- and Stx2-producing STEC O157 strain Tokyo-EH5487 (STEC O157 Tokyo) associated with the cucumber outbreak in Chiba and Tokyo in 2016 was obtained from the Department of Food Microbiology at the Tokyo Metropolitan Institute of Public Health. Stx1- and Stx2-producing STEC O157:H7 strain Sakai (STEC O157 Sakai) (RIMD 0509939) was purchased from the Pathogenic Microbes Repository Unit of Osaka University. To obtain bacteria resistant to streptomycin (SM) and mitomycin C (MMC), STEC were grown in nutrient agar supplemented with 100 μg/mL SM (Wako Pure Chemical Industries, Osaka, Japan) and 0.5 μg/mL MMC (Kyowa Hakko Kogyo, Tokyo, Japan).

### Stx2a Purification

Stx2a was immunoaffinity purified from a clinical isolate of STEC.^55^ The Stx2a was determined to be free of detectable LPS by the Toxicolor test, SDS-PAGE, and silver staining.

### Preparation of Muse and Non-Muse Cells

Muse and non-Muse cells were prepared from adult human BM-MSCs (Lonza, MD, USA) as described previously.^26^ Non-Muse cells and cells other than Muse cells in BM-MSCs are hereafter referred to as SSEA-3^−^ cells. SSEA-3^+^ (Muse cells) and SSEA-3^−^ (non-Muse cells) were separated according to previous reports.^26^ To prepare GFP-labeled Muse and non-Muse cells, BM-MSCs were infected with a lentivirus encoding GFP at an efficiency of ∼70% as described previously.^26^ GFP-expressing BM-MSCs were separated by positive expression of SSEA-3. GFP-Muse cells were double-positive for GFP and SSEA-3, whereas GFP-non-Muse cells were positive for GFP but negative for SSEA-3. The use of these cells was not in conflict with any regulations of our university.

### STEC Orally Infected Mouse Models and Muse or Non-Muse Cell Injections

A mouse model was established with STEC inoculated orally and MMC administered via intraperitoneal (i.p.) injection according to the previous report.^11^ In brief, all mice were provided with SM-containing drinking water (5 g/L) *ad libitum* to reduce normal intestinal flora. On day 3 of SM treatment, mice were deprived of food for 12 h until bacterial inoculation in order to avoid absorption of inoculated STECs by food or preventing disturbance of STEC colonization by food.^11^, ^56^ Each mouse was orogastrically inoculated with 0.5 mL of a bacterial suspension using a syringe attached to a sterile disposable feeding needle (diameter, 1.9 mm; length, 38 mm; Fuchigami, Nara, Japan) passed into the stomach. Simultaneously, they were injected i.p. with 2.5 μg/g MMC. In this study, bacterial suspensions of EHEC O111 and EHEC O157 (E32511) strains (hereafter referred to as E32511), the Stx1- and Stx2-producing STEC O157 strain Tokyo-EH5487 (hereafter referred to as STEC O157 Tokyo), and the Stx1- and Stx2-producing STEC O157:H7 strain Sakai (hereafter referred to as STEC O157 Sakai) were adjusted to the appropriate CFU in PBS. After treatment, mice were returned to their respective housing and given food and SM-containing drinking water (5 g/L) *ad libitum*. Mice were observed every 12 h for survival and signs of illness, and body weight measurements were performed. To avoid technical errors associated with oral STEC inoculation, mice were inoculated with *E. coli* K-12 (SMr and MMCr) and survived (100%) (data not shown). Therefore, in this study, technical errors were not described in the Results.

### Bioluminescence Imaging

Bioluminescence imaging with an IVIS was conducted as described previously.^57^ In brief, eight mice were infected with 1 × 10^10^ CFU STEC E32511 or O111 (four mice for each strain). After 1 or 2 days, mice were intravenously injected with nano-lantern-labeled Muse or non-Muse cells (1 × 10^5^/mouse).^25^ Mice were then intravenously injected with 100 μL substrate (80 μg/mL coelenterazine-h; Wako, Tokyo, Japan), anesthetized with isoflurane by inhalation, and placed in an acrylic plastic box. Bioluminescent images were acquired using an IVIS (Spectrum; PerkinElmer, Waltham, MA, USA) using the following settings: exposure time, 1 min; medium binning, F/Stop = 1. Data acquisition and analysis were performed using Living Image 4.4 software (PerkinElmer). Quantification was performed using a region of interest (ROI) defined manually (abdominal center or injection site), and the results are expressed as photons per second (P/s).

### Immunohistochemistry with Tyramide Signal Amplification

Immunohistochemistry with tyramide signal amplification (mIHC) was performed as described by the protocol from Cell Signaling Technology (https://en.cellsignal.jp/contents/resources-protocols/fluorescent-multiplex-immunohistochemistry-(mihc)-with-tyramide-signal-amplification-protocol/fluorescent-multiplex-ihc-w-tyramide). In brief, paraffin-embedded mouse brain tissues were deparaffinized with xylene and then rehydrated with 100% ethanol, 95% ethanol, and dH_2_O. For antigenic unmasking, tissue sections were boiled in sodium citrate buffer (pH 6.0) in a microwave. Sections were quenched by incubation in 3% hydrogen peroxide for 10 min. Anti-mouse CD31, anti-mouse β3-tubulin, and anti-COX IV rabbit monoclonal antibodies were purchased from Cell Signaling Technology (Danvers, MA, USA). A ready-to-use anti-GFAP rabbit polyclonal antibody (Dako) was also used as a primary antibody. Primary antibodies were diluted in SignalStain Antibody Diluent (Cell Signaling Technology), and sections were incubated under humidified conditions at room temperature. Subsequent incubations with SignalStain Boost IHC Detection Reagent (rabbit-horseradish peroxidase [HRP]; Cell Signaling Technology) were performed in a humidified chamber. Tyramide signal amplification (TSA) was performed using the TSA Plus Cyanine 3/Fluorescein System (PerkinElmer, Waltham, MA, USA), according to the manufacturer’s recommendations. Sections were incubated in tyramide-fluorophore conjugate and amplification reagent for 10 min at room temperature in a humidified chamber while protected from light. For double staining, sections were first stripped by boiling in sodium citrate buffer (pH 6.0) in a microwave and then stained using a different tyramide-fluorophore conjugate. Three rounds of double staining were performed using the TSA Plus Cyanine 3/Fluorescein System. Cells were stained with an anti-COX IV rabbit monoclonal antibody (1:1,000; Cell Signaling Technology) counterstained with cyanine 3 (Thermo Fisher Scientific, Invitrogen) along with one of the following antibodies (all counterstained with fluorescein): (1) anti-CD31 (1:800; Cell Signaling Technology), (2) diluted ready-to-use anti-GFAP (1:10; Dako), or (3) anti-β3-tubulin (1:2,000; Cell Signaling Technology). All sections were mounted with coverslips using ProLong Gold Antifade Reagent with DAPI (Cell Signaling Technology). Specimens were examined under the BZ-X700 HS All-in-One Fluorescence Microscope and a LSM780 confocal microscope (ZEISS Research, Japan). Using ZEN version 2.3 (blue edition) software of the confocal microscope, we made three dimensional (3D) images.

### Forensic Autopsies in the Outbreak of STEC O111 and O157 Infections in Japan

Human brain tissues were obtained from forensic autopsies of three individuals. Paraffin-embedded brain sections were obtained from a 43-year-old woman with brain edema and hernia, who subsequently died of STEC infection in the Toyama prefecture during an outbreak of Stx2-producing STEC O111 and O157 infections that occurred in Japan in 2011. The autopsy was performed at the Department of Legal Medicine, Graduate School of Medicine and Pharmaceutical Sciences, University of Toyama. Control paraffin-embedded brain sections from a 43-year-old male were provided by the Division of Neuropathology, Tottori University. In addition, paraffin-embedded brain sections without any brain lesions were provided for three females (82, 88, and 90 years of age), who died in the Tokyo outbreak of 2016 from STEC O157:H7 caused by cucumbers. Their autopsies were performed at the Department of Forensic Medicine, Jikei University School of Medicine. Age- and sex-adjusted control samples (80- and 89-year-old females) were provided by the Division of Legal Medicine, Department of Social Medicine, Faculty of Medicine, Tottori University. All human autopsy and control brain sections were stained with H&E and GFAP immunohistochemistry. All procedures were performed in accordance with the guidelines of the Japanese Society of Legal Medicine.

### Immunohistochemistry of Paraffin-Embedded Sections

Mice were anesthetized with sevoflurane (Maruishi Pharmaceutical, Osaka, Japan). Then, the mice were perfused using a Perista pump (SJ-1211II-H; ATTO, Japan) from the left ventricle with 10 mL PBS and 40 mL of 4% paraformaldehyde (PFA; Wako Laboratory Chemicals, Osaka, Japan) in PBS for fixation. The brain and spinal cord were harvested and fixed in 4% PFA in PBS. The brain was then processed for immunohistochemical examination. Formalin-fixed, paraffin-embedded tissue blocks were cut into 6-μm-thick sections. For immunohistochemistry (IHC), tissue sections were deparaffinized with xylene and rehydrated through an ethanol series and Tris-buffered saline (TBS). Antigen retrieval was performed by enzyme treatment with Proteinase K (Takara Bio, Shiga, Japan). Endogenous peroxidase activity was blocked with 0.3% H_2_O_2_ in methanol for 30 min, followed by incubation with Protein Block (Genostaff, Tokyo, Japan). Sections were incubated with undiluted rabbit polyclonal antibody against GFAP (1 μg/mL; Dako Denmark, Glostrup, Denmark) at 4°C overnight and then with EnVision+ Single Reagent (Dako) for 30 min at room temperature. Peroxidase activity was visualized by diaminobenzidine. Alternatively, sections were incubated with an anti-human COX IV rabbit monoclonal antibody (1:2,000; Cell Signaling Technology, Beverly, MA, USA) at 4°C overnight, followed by biotin-conjugated goat anti-rabbit Ig (1:600; Agilent Dako Technologies) for 30 min at room temperature. Sections were then incubated with peroxidase-conjugated streptavidin (Nichirei, Tokyo, Japan) for 5 min. All sections were counterstained with Mayer’s hematoxylin (MUTO Pure Chemicals, Tokyo, Japan), dehydrated, and then mounted with Malinol (MUTO Pure Chemicals). Specimens were examined using the BZ-X700 HS All-in-One Fluorescence Microscope. The numbers and areas of Muse/non-Muse cells or reactive astrocytes were quantified with a BZ-X Analyzer and the color extraction mode of the Hybrid Cell Count software (Keyence). mIHC was performed to human Muse or non-Muse cells in O111-infected mice as described above. In brief, paraffin-embedded mouse brain tissues were deparaffinized with xylene and then rehydrated with 100% ethanol, 95% ethanol, and dH_2_O. For antigenic unmasking, tissue sections were boiled in sodium citrate buffer (pH 6.0) in a microwave. Sections were quenched by incubation in 3% hydrogen peroxide for 10 min. Anti-COX IV rabbit monoclonal antibodies were purchased from Cell Signaling Technology (Danvers, MA, USA). Primary antibodies were diluted in SignalStain Antibody Diluent (Cell Signaling Technology), and sections were incubated under humidified conditions at room temperature. Subsequent incubations with SignalStain Boost IHC Detection Reagent (rabbit-HRP; Cell Signaling Technology) were performed in a humidified chamber. Tyramide signal amplification (TSA) was performed using the TSA Plus Cyanine 3/Fluorescein System (PerkinElmer, Waltham, MA, USA), according to the manufacturer’s recommendations. Sections were incubated in tyramide-fluorophore conjugate and amplification reagent for 10 min at room temperature in a humidified chamber while protected from light. For the staining of Muse or non-Muse cells, sections were first stripped by boiling in sodium citrate buffer (pH 6.0) in a microwave and then stained using a tyramide-fluorophore conjugate. The staining was performed using the TSA Plus Cyanine 3/Fluorescein System. Cells were stained with an anti-COX IV rabbit monoclonal antibody (1:1,000; Cell Signaling Technology) counterstained with cyanine 3 (Thermo Fisher Scientific, Invitrogen). The sections were mounted with coverslips. Specimens were examined under the BZ-X700 HS All-in-One Fluorescence Microscope to count the total area that contained Muse cells and the number of engrafted Muse cells with BZ-X analyzer.

### May-Grunwald-Giemsa Staining and Western Blot Assay

Mouse C57 mixed astrocytes (astrocytes) (M-AsM-330; Lonza, Walkersville, MD, USA) were maintained in ABM astrocyte basal medium (CC-3187; Lonza). For *in vitro* experiments, astrocytes were seeded in two-well Lab-Tek chamber slides with covers (Nalge Nunc International, Roskilde, Denmark) and incubated at 37°C with 5% CO_2_. Stx2a- and/or LPS-treated activated astrocytes (reactive astrocytes) were subjected to May-Grunwald-Giemsa staining using an automated hematology slide preparation machine (SP-10; Sysmex, Kobe, Japan). Images of normal astrocytes and reactive astrocytes were captured using the All-in-One Microscope (Keyence) under a 40× objective, and the area was calculated using ImageJ 1.52a (NIH, Bethesda, MD, USA). The number of reactive astrocytes was counted in four fields for each chamber slide, and the mean of three samples was calculated. The area of reactive astrocytes was more than 4-fold that of control astrocytes. To observe reactive astrocytes in a dose- and time-dependent manner, reactive astrocytes were lysed and subjected to western blot assay. Protein expression was quantified using the Lumino Image Analyzer LAS1000 plus (FUJIFILM, Tokyo, Japan). The hallmark of astrocyte activation is enhanced expression of the major intermediate filament protein GFAP that was detected by western blot assay using an anti-mouse GFAP rabbit antibody (Dako) and HRP-conjugated antibody (Sigma-Aldrich, St. Louis, MO, USA) as described previously by Bitko and Barik.^58^ Immunodetection was carried out using the ECL detection system (Amersham Pharmacia Biotech, UK).

### GFP Labeling of Muse and Non-Muse Cells

Human BM-mesenchymal stem cells (MSCs) (Lonza) were infected with a lentivirus encoding GFP, and Muse cells were collected as SSEA3^+^ cells. For immunofluorescence staining, mouse astrocytes and Muse/non-Muse cells (10,000 cells each) were co-cultured overnight in two-well chamber slides (Nalge Nunc International). The next day, the medium was discarded and several concentrations of Stx2 and 1 μg/mL purified LPS derived from *E. coli* O55:B5 (Sigma-Aldrich) were used to induce reactive astrocytes. After 6 or 12 h, cells were fixed with 4% PFA for 30 min and then washed twice with PBS. Cells were blocked with blocking buffer (5% normal goat serum/0.3% Triton X-100 in PBS) for 1 h at room temperature and immunostained overnight at 4°C with an anti-GFAP mouse monoclonal antibody (Alexa Fluor 594, #8152; Cell Signaling Technology) diluted by a factor of 100 with dilution buffer (1% BSA/0.3% Triton X-100 in PBS). Samples were stained with DAPI, washed twice with PBS, and mounted with SlowFade antifade solution (Invitrogen). Specimens were examined under the BZ-X700 HS All-in-One Fluorescence Microscope (Keyence, Osaka, Japan).

### Microarray Analysis

First, 100,000 primary astrocytes were placed into a T75 cell culture flask (75 cm^3^ for adherent cells; Nalge Nunc International) in ABM astrocyte basal medium and incubated at 37°C with 5% CO_2_. After 18 h, ABM was replaced with fresh ABM, along with 100,000 human non-Muse or Muse cells and 10 ng Stx2 and 1 μg/mL LPS. At 6 or 12 h after incubation, reactive astrocytes and Muse or non-Muse cells were detached with 0.25% trypsin (GIBCO), and the Muse or non-Muse cells were separated from the reactive astrocytes using human CD105 MicroBeads (Miltenyi Biotec) and a magnetic separator (MiniMACS Separator; Miltenyi Biotec). Total RNA was isolated from Muse and non-Muse cells individually using TRIzol Reagent (Life Technologies) and purified using the SV Total RNA Isolation System (Promega), in accordance with the manufacturers’ instructions. Also, the reactive astrocytes were collected in TRIzol Reagent and are being analyzed currently. RNA samples were quantified by an ND-1000 spectrophotometer (NanoDrop Technologies, Wilmington, DE, USA), and the quality was confirmed with a 2200 TapeStation (Agilent Technologies, Santa Clara, CA, USA). cRNA was amplified, labeled, and hybridized to a 60K Agilent 60-mer oligomicroarray (Agilent Trusted Answers, CA, USA) using the SurePrint G3 Human Gene Expression Microarray 8 × 60K v3 labeling kit (Low Input Quick Amp Labeling Kit; #5190-2305; Agilent Trusted Answers, CA, USA), in accordance with the manufacturer’s instructions. All hybridized microarray slides were scanned using an Agilent scanner. Relative hybridization intensities and background hybridization values were calculated using Agilent Feature Extraction Software (9.5.1.1). Raw signal intensities and Flags for each probe were calculated from hybridization intensities (gProcessedSignal) and spot information (gIsSaturated), according to the procedures recommended by Agilent. Flag criteria on GeneSpring Software included: Absent (A), “Feature is not positive and significant” and “Feature is not above background”; Marginal (M), “Feature is not Uniform,” “Feature is Saturated,” “Feature is a population outlier”; and Present (P), others. The raw signal intensities of two samples were log2-transformed and normalized by the quantile algorithm with the “preprocessCore” library package [P] of Bioconductor software [B]. We selected probes that called the “P” flag in at least one sample, excluding long intergenic non coding RNA (lincRNA) probes.

### Microarray and Network Analysis

IPA version 45868156 was used to identify upregulated functionally significant genes. To this end, we required the ratio of gene scores for Muse cells versus non-Muse cells, comparing the results at 6 and 12 h after incubation with reactive astrocytes, Stx2, and LPS. Using the IPA tool, networks were generated based on an algorithmically generated score. The *Z* score was used to rank networks according to their relevance to genes present within the dataset. Canonical pathways significant to the input dataset were identified from the IPA library of neuronal differentiation pathways based on two parameters, viz.: (1) the ratio of the number of genes within the dataset mapping to the pathway divided by the total number of genes mapping to the neuronal differentiation pathways; and (2) the p value (calculated based on Fischer’s exact test), which determined the probability that each bio-function assigned to that dataset and canonical pathway was not due to chance alone. Upstream regulators were defined as genes that affected the expression of numerous other genes, whereas canonical pathways were defined as the idealized or generalized pathways that represent common properties of a particular signaling module or pathway.

### RNA Quantification by Digital PCR

Ten thousand primary astrocytes were seeded in six-well culture plates (Nalge Nunc International) in ABM and incubated at 37°C with 5% CO_2_ for 18 h. Culture medium was replenished, along with the addition of 10,000 human non-Muse or Muse cells, and 10 ng Stx2 and 1 μg/mL LPS. After 6 or 12 h, the cells were detached with 0.25% trypsin (GIBCO), and the Muse and non-Muse cells were separated from reactive astrocytes using CD105 MicroBeads. Total RNA was extracted using the PureLink RNA Mini Kit (Thermo Fisher Scientific), according to the manufacturer’s instructions. The RNA concentration was measured using a NanoDrop Lite Spectrophotometer (Thermo Fisher Scientific). cDNA was synthesized using SuperScript IV VILOTM Master Mix (Thermo Fisher Scientific), according to the manufacturer’s instructions. Samples measured by the QuantStudio 3D Digital PCR System (Life Technologies) were loaded onto chips using the QuantStudio 3D Digital PCR Chip Loader in a mixture consisting of QuantStudio 3D digital PCR Master Mix v2 (Thermo Fisher Scientific), primers, and FAM dye-labeled MGB probes for digital PCR of CSF3 (assay ID: Hs00783432_g1; Thermo Fisher Scientific) or the eukaryotic 18S rRNA endogenous control (Thermo Fisher Scientific). Chips were sealed and loaded onto a GeneAMP PCR system 9700 (Applied Biosystems) and cycled according to the following parameters: 96°C for 10 min, followed by 39 cycles of 56°C for 2 min and 98°C for 30 s with a final extension of 56°C for 2 min. After cycling, the endpoint fluorescence of the partitions on the chips was measured by transferring the chips to the measurement unit (v3.0). Applied Biosystems QuantStudio 3D Analysis Suite Cloud Software was used to analyze and refine the data.

### RNAi of G-CSF (i.e., CSF3)

For CSF3 (accession number NM_000759.3) mRNA knockdown, three Silencer Select predesigned siRNAs (s3606, s3607, and s6308) and NC No. 1 were purchased from Invitrogen. Human Muse cells (3 × 10^4^) growing in six-well culture dishes were incubated for 48 h in 1 mL complete medium containing a mixture of 1 μL Lipofectamine RNAiMax (Invitrogen) and 5 or 10 nM stealth siRNA against human CSF3. CSF3 knockdown in human Muse cells was confirmed by the relative expression of CSF3 mRNA.^59^

### Statistical Analysis

Survival rates were compared using Kaplan-Meier tests followed by pairwise comparisons using the log rank (Mantel-Cox) test. Differences in weight changes were tested by general linear model repeated measures with Scheffe’s and Tukey’s post hoc tests. Statistical differences were considered significant at p values of less than 0.05 and 0.01. All statistical analyses were conducted using IBM SPSS Statistics v24.0.

## Author Contributions

J.F., N.O., M.I., N.N., Sari Matsumoto, K.I., N.K., S.K., E.Y., Sohkichi Matsumoto, and M.D. generated the hypotheses and conceptualized the study. R.O., T.T.,T.M., M.Y.A., M.H., and A.Y. carried out animal work and coordinated the project. S.W. provided Muse and non-Muse, GFP-labeled, and nano-Lantern-labeled Muse and non-Muse cells. R.O. performed histopathology studies with immunostainings and all-in-one microscopy. K.Y. and K.T. analyzed the microarray. Y.K. performed statistical analysis. J.F. and M.D. wrote the manuscript. All authors read and approved the final draft of the manuscript.

## Conflicts of Interest

The authors declare no competing interests.

## Supplemental Information

Document S1. Figures S1–S7, Supplemental Materials and Methods, and Tables S1 and S2

Document S2. Article plus Supplemental Information

## References

[1] Trachtman H., Austin C., Lewinski M., Stahl R.A. (2012). Renal and neurological involvement in typical Shiga toxin-associated HUS. Nat. Rev. Nephrol..

[2] Keir L.S., Saleem M.A. (2014). Current evidence for the role of complement in the pathogenesis of Shiga toxin haemolytic uraemic syndrome. Pediatr. Nephrol..

[3] O’Brien A.D., Holmes R.K. (1987). Shiga and Shiga-like toxins. Microbiol. Rev..

[4] Lingwood C.A., Law H., Richardson S., Petric M., Brunton J.L., De Grandis S., Karmali M. (1987). Glycolipid binding of purified and recombinant *Escherichia coli* produced verotoxin in vitro. J. Biol. Chem..

[5] Louise C.B., Obrig T.G. (1991). Shiga toxin-associated hemolytic-uremic syndrome: combined cytotoxic effects of Shiga toxin, interleukin-1 beta, and tumor necrosis factor alpha on human vascular endothelial cells in vitro. Infect. Immun..

[6] Louise C.B., Obrig T.G. (1992). Shiga toxin-associated hemolytic uremic syndrome: combined cytotoxic effects of shiga toxin and lipopolysaccharide (endotoxin) on human vascular endothelial cells in vitro. Infect. Immun..

[7] Mallard F., Antony C., Tenza D., Salamero J., Goud B., Johannes L. (1998). Direct pathway from early/recycling endosomes to the Golgi apparatus revealed through the study of shiga toxin B-fragment transport. J. Cell Biol..

[8] Endo Y., Tsurugi K., Yutsudo T., Takeda Y., Ogasawara T., Igarashi K. (1988). Site of action of a Vero toxin (VT2) from *Escherichia coli* O157:H7 and of Shiga toxin on eukaryotic ribosomes. RNA N-glycosidase activity of the toxins. Eur. J. Biochem..

[9] Obrig T.G., Moran T.P., Brown J.E. (1987). The mode of action of Shiga toxin on peptide elongation of eukaryotic protein synthesis. Biochem. J..

[10] Karpman D., Loos S., Tati R., Arvidsson I. (2017). Haemolytic uraemic syndrome. J. Intern. Med..

[11] Fujii J., Kita T., Yoshida S., Takeda T., Kobayashi H., Tanaka N., Ohsato K., Mizuguchi Y. (1994). Direct evidence of neuron impairment by oral infection with verotoxin-producing *Escherichia coli* O157:H- in mitomycin-treated mice. Infect. Immun..

[12] Amran M.Y., Fujii J., Suzuki S.O., Kolling G.L., Villanueva S.Y., Kainuma M., Kobayashi H., Kameyama H., Yoshida S. (2013). Investigation of encephalopathy caused by Shiga toxin 2c-producing *Escherichia coli* infection in mice. PLoS ONE.

[13] Fujii J., Kinoshita Y., Kita T., Higure A., Takeda T., Tanaka N., Yoshida S. (1996). Magnetic resonance imaging and histopathological study of brain lesions in rabbits given intravenous verotoxin 2. Infect. Immun..

[14] Hamano S., Nakanishi Y., Nara T., Seki T., Ohtani T., Oishi T., Joh K., Oikawa T., Muramatsu Y., Ogawa Y. (1993). Neurological manifestations of hemorrhagic colitis in the outbreak of *Escherichia coli* O157:H7 infection in Japan. Acta Paediatr..

[15] Boccoli J., Loidl C.F., Lopez-Costa J.J., Creydt V.P., Ibarra C., Goldstein J. (2008). Intracerebroventricular administration of Shiga toxin type 2 altered the expression levels of neuronal nitric oxide synthase and glial fibrillary acidic protein in rat brains. Brain Res..

[16] Landoni V.I., de Campos-Nebel M., Schierloh P., Calatayud C., Fernandez G.C., Ramos M.V., Rearte B., Palermo M.S., Isturiz M.A. (2010). Shiga toxin 1-induced inflammatory response in lipopolysaccharide-sensitized astrocytes is mediated by endogenous tumor necrosis factor alpha. Infect. Immun..

[17] Landoni V.I., Schierloh P., de Campos Nebel M., Fernández G.C., Calatayud C., Lapponi M.J., Isturiz M.A. (2012). Shiga toxin 1 induces on lipopolysaccharide-treated astrocytes the release of tumor necrosis factor-alpha that alter brain-like endothelium integrity. PLoS Pathog..

[18] Kuroda Y., Kitada M., Wakao S., Nishikawa K., Tanimura Y., Makinoshima H., Goda M., Akashi H., Inutsuka A., Niwa A. (2010). Unique multipotent cells in adult human mesenchymal cell populations. Proc. Natl. Acad. Sci. USA.

[19] Wakao S., Kuroda Y., Ogura F., Shigemoto T., Dezawa M. (2012). Regenerative Effects of Mesenchymal Stem Cells: Contribution of Muse Cells, a Novel Pluripotent Stem Cell Type that Resides in Mesenchymal Cells. Cells.

[20] Yamada Y., Wakao S., Kushida Y., Minatoguchi S., Mikami A., Higashi K., Baba S., Shigemoto T., Kuroda Y., Kanamori H. (2018). S1P-S1PR2 Axis Mediates Homing of Muse Cells Into Damaged Heart for Long-Lasting Tissue Repair and Functional Recovery After Acute Myocardial Infarction. Circ. Res..

[21] Kinoshita K., Kuno S., Ishimine H., Aoi N., Mineda K., Kato H., Doi K., Kanayama K., Feng J., Mashiko T. (2015). Therapeutic Potential of Adipose-Derived SSEA-3-Positive Muse Cells for Treating Diabetic Skin Ulcers. Stem Cells Transl. Med..

[22] Mineda K., Feng J., Ishimine H., Takada H., Doi K., Kuno S., Kinoshita K., Kanayama K., Kato H., Mashiko T. (2015). Therapeutic Potential of Human Adipose-Derived Stem/Stromal Cell Microspheroids Prepared by Three-Dimensional Culture in Non-Cross-Linked Hyaluronic Acid Gel. Stem Cells Transl. Med..

[23] Uchida N., Kushida Y., Kitada M., Wakao S., Kumagai N., Kuroda Y., Kondo Y., Hirohara Y., Kure S., Chazenbalk G., Dezawa M. (2017). Beneficial Effects of Systemically Administered Human Muse Cells in Adriamycin Nephropathy. J. Am. Soc. Nephrol..

[24] Hosoyama K., Saiki Y. (2018). Muse Cells and Aortic Aneurysm. Adv. Exp. Med. Biol..

[25] Dezawa M. (2018). Clinical Trials of Muse Cells. Adv. Exp. Med. Biol..

[26] Kuroda Y., Wakao S., Kitada M., Murakami T., Nojima M., Dezawa M. (2013). Isolation, culture and evaluation of multilineage-differentiating stress-enduring (Muse) cells. Nat. Protoc..

[27] Wakao S., Kushida Y., Dezawa M. (2018). Basic Characteristics of Muse Cells. Adv. Exp. Med. Biol..

[28] Alessio N., Özcan S., Tatsumi K., Murat A., Peluso G., Dezawa M., Galderisi U. (2017). The secretome of Muse cells contains factors that may play a role in regulation of stemness, apoptosis and immunomodulation. Cell Cycle.

[29] Alessio N., Squillaro T., Özcan S., Di Bernardo G., Venditti M., Melone M., Peluso G., Galderisi U. (2018). Stress and stem cells: adult Muse cells tolerate extensive genotoxic stimuli better than mesenchymal stromal cells. Oncotarget.

[30] Iseki M., Kushida Y., Wakao S., Akimoto T., Mizuma M., Motoi F., Asada R., Shimizu S., Unno M., Chazenbalk G., Dezawa M. (2017). Muse Cells, Nontumorigenic Pluripotent-Like Stem Cells, Have Liver Regeneration Capacity Through Specific Homing and Cell Replacement in a Mouse Model of Liver Fibrosis. Cell Transplant..

[31] Uchida H., Morita T., Niizuma K., Kushida Y., Kuroda Y., Wakao S., Sakata H., Matsuzaka Y., Mushiake H., Tominaga T. (2016). Transplantation of Unique Subpopulation of Fibroblasts, Muse Cells, Ameliorates Experimental Stroke Possibly via Robust Neuronal Differentiation. Stem Cells.

[32] Yahata Y., Misaki T., Ishida Y., Nagira M., Watahiki M., Isobe J., Terajima J., Iyoda S., Mitobe J., Ohnishi M., *E. coli* O111 Outbreak Investigation Team (2015). Epidemiological analysis of a large enterohaemorrhagic Escherichia coli O111 outbreak in Japan associated with haemolytic uraemic syndrome and acute encephalopathy. Epidemiol. Infect..

[33] Yamada Y., Fujii J., Murasato Y., Nakamura T., Hayashida Y., Kinoshita Y., Yutsudo T., Matsumoto T., Yoshida S. (1999). Brainstem mechanisms of autonomic dysfunction in encephalopathy-associated Shiga toxin 2 intoxication. Ann. Neurol..

[34] Fujii J., Kinoshita Y., Matsukawa A., Villanueva S.Y., Yutsudo T., Yoshida S. (2009). Successful steroid pulse therapy for brain lesion caused by Shiga toxin 2 in rabbits. Microb. Pathog..

[35] Pinto A., Jacobsen M., Geoghegan P.A., Cangelosi A., Cejudo M.L., Tironi-Farinati C., Goldstein J. (2013). Dexamethasone rescues neurovascular unit integrity from cell damage caused by systemic administration of shiga toxin 2 and lipopolysaccharide in mice motor cortex. PLoS ONE.

[36] Cameron P., Smith S.J., Giembycz M.A., Rotondo D., Plevin R. (2003). Verotoxin activates mitogen-activated protein kinase in human peripheral blood monocytes: role in apoptosis and proinflammatory cytokine release. Br. J. Pharmacol..

[37] Cherla R.P., Lee S.Y., Mees P.L., Tesh V.L. (2006). Shiga toxin 1-induced cytokine production is mediated by MAP kinase pathways and translation initiation factor eIF4E in the macrophage-like THP-1 cell line. J. Leukoc. Biol..

[38] Lucero M.S., Mirarchi F., Goldstein J., Silberstein C. (2012). Intraperitoneal administration of Shiga toxin 2 induced neuronal alterations and reduced the expression levels of aquaporin 1 and aquaporin 4 in rat brain. Microb. Pathog..

[39] Tironi-Farinati C., Geoghegan P.A., Cangelosi A., Pinto A., Loidl C.F., Goldstein J. (2013). A translational murine model of sub-lethal intoxication with Shiga toxin 2 reveals novel ultrastructural findings in the brain striatum. PLoS ONE.

[40] Liddelow S.A., Guttenplan K.A., Clarke L.E., Bennett F.C., Bohlen C.J., Schirmer L., Bennett M.L., Münch A.E., Chung W.S., Peterson T.C. (2017). Neurotoxic reactive astrocytes are induced by activated microglia. Nature.

[41] Siegler R.L. (1995). The hemolytic uremic syndrome. Pediatr. Clin. North Am..

[42] Fujii J., Mizoue T., Kita T., Kishimoto H., Joh K., Nakada Y., Ugajin S., Naya Y., Nakamura T., Tada Y. (2016). Risk of haemolytic uraemic syndrome caused by shiga-toxin-producing *Escherichia coli* infection in adult women in Japan. Epidemiol. Infect..

[43] Schlieper A., Orrbine E., Wells G.A., Clulow M., McLaine P.N., Rowe P.C. (1999). Neuropsychological sequelae of haemolytic uraemic syndrome. Investigators of the HUS Cognitive Study. Arch. Dis. Child..

[44] Schuppner R., Maehlmann J., Dirks M., Worthmann H., Tryc A.B., Sandorski K., Bahlmann E., Kielstein J.T., Giesemann A.M., Lanfermann H., Weissenborn K. (2016). Neurological Sequelae in Adults After *E*. *coli* O104: H4 Infection-Induced Hemolytic-Uremic Syndrome. Medicine (Baltimore).

[45] Greinacher A., Friesecke S., Abel P., Dressel A., Stracke S., Fiene M., Ernst F., Selleng K., Weissenborn K., Schmidt B.M. (2011). Treatment of severe neurological deficits with IgG depletion through immunoadsorption in patients with *Escherichia coli* O104:H4-associated haemolytic uraemic syndrome: a prospective trial. Lancet.

[46] Fujii J., Matsui T., Heatherly D.P., Schlegel K.H., Lobo P.I., Yutsudo T., Ciraolo G.M., Morris R.E., Obrig T. (2003). Rapid apoptosis induced by Shiga toxin in HeLa cells. Infect. Immun..

[47] Oyadomari S., Mori M. (2004). Roles of CHOP/GADD153 in endoplasmic reticulum stress. Cell Death Differ..

[48] Fujii J., Wood K., Matsuda F., Carneiro-Filho B.A., Schlegel K.H., Yutsudo T., Binnington-Boyd B., Lingwood C.A., Obata F., Kim K.S. (2008). Shiga toxin 2 causes apoptosis in human brain microvascular endothelial cells via C/EBP homologous protein. Infect. Immun..

[49] Cosler L.E., Eldar-Lissai A., Culakova E., Kuderer N.M., Dale D., Crawford J., Lyman G.H. (2007). Therapeutic use of granulocyte colony-stimulating factors for established febrile neutropenia: effect on costs from a hospital perspective. Pharmacoeconomics.

[50] Basu S., Hodgson G., Katz M., Dunn A.R. (2002). Evaluation of role of G-CSF in the production, survival, and release of neutrophils from bone marrow into circulation. Blood.

[51] Abdel-Latif A., Bolli R., Zuba-Surma E.K., Tleyjeh I.M., Hornung C.A., Dawn B. (2008). Granulocyte colony-stimulating factor therapy for cardiac repair after acute myocardial infarction: a systematic review and meta-analysis of randomized controlled trials. Am. Heart J..

[52] Menzie-Suderam J.M., Mohammad-Gharibani P., Modi J., Ma Z., Tao R., Prentice H., Wu J.Y. (2018). Granulocyte-colony stimulating factor protects against endoplasmic reticulum stress in an experimental model of stroke. Brain Res..

[53] Chu H., Tang Y., Dong Q. (2014). Protection of granulocyte-colony stimulating factor to hemorrhagic brain injuries and its involved mechanisms: effects of vascular endothelial growth factor and aquaporin-4. Neuroscience.

[54] Dietrich J., Baryawno N., Nayyar N., Valtis Y.K., Yang B., Ly I., Besnard A., Severe N., Gustafsson K.U., Andronesi O.C. (2018). Bone marrow drives central nervous system regeneration after radiation injury. J. Clin. Invest..

[55] Louise C.B., Obrig T.G. (1995). Specific interaction of *Escherichia coli* O157:H7-derived Shiga-like toxin II with human renal endothelial cells. J. Infect. Dis..

[56] Myhal M.L., Laux D.C., Cohen P.S. (1982). Relative colonizing abilities of human fecal and K 12 strains of *Escherichia coli* in the large intestines of streptomycin-treated mice. Eur. J. Clin. Microbiol..

[57] Ozuru R., Saito M., Kanemaru T., Miyahara S., Villanueva S.Y., Murray G.L., Adler B., Fujii J., Yoshida S.I. (2017). Adipose tissue is the first colonization site of *Leptospira interrogans* in subcutaneously infected hamsters. PLoS ONE.

[58] Bitko V., Barik S. (1998). Persistent activation of RelA by respiratory syncytial virus involves protein kinase C, underphosphorylated IκBβ, and sequestration of protein phosphatase 2A by the viral phosphoprotein. J. Virol..

[59] Yamada N., Kuranaga Y., Kumazaki M., Shinohara H., Taniguchi K., Akao Y. (2016). Colorectal cancer cell-derived extracellular vesicles induce phenotypic alteration of T cells into tumor-growth supporting cells with transforming growth factor-β1-mediated suppression. Oncotarget.

