# Rescue from Stx2-Producing *E. coli*-Associated Encephalopathy by Intravenous Injection of Muse Cells in NOD-SCID Mice

**DOI:** 10.1016/j.ymthe.2025.01.022

**Published:** 2025-01-28

**Authors:** Ryo Ozuru, Shohei Wakao, Takahiro Tsuji, Naoya Ohara, Takashi Matsuba, Muhammad Y. Amran, Junko Isobe, Morio Iino, Naoki Nishida, Sari Matsumoto, Kimiharu Iwadate, Noriko Konishi, Kaori Yasuda, Kosuke Tashiro, Misato Hida, Arisato Yadoiwa, Shinsuke Kato, Eijiro Yamashita, Sohkichi Matsumoto, Yoichi Kurozawa, Mari Dezawa, Jun Fujii

## Main text

(Molecular Therapy *28*, 100–118; January 2020)

In the originally published article, the spelling of one author’s name was given incorrectly. The authors apologize to the readers and Dr. Amran for this oversight. The author’s name has been corrected as follows: Muhammad Yunus Amran.

