# Supplementary material for: Rescue from Stx2-Producing E. coli-Associated Encephalopathy by Intravenous Injection of Muse Cells in NOD-SCID Mice
Source: Mol Ther. 2019 Oct 1;28(1):100–18. doi: 10.1016/j.ymthe.2019.09.023 (PMC6953779; doi:10.1016/j.ymthe.2019.09.023)
Supplement: Document S1. Figures S1–S7, Supplemental Materials and Methods, and Tables S1 and S2 [file mmc1.pdf]

YMTHE, Volume *28*

Supplemental Information

Rescue from Stx2-Producing *E. coli*-Associated Encephalopathy by Intravenous Injection of Muse Cells in NOD-SCID Mice

Ryo Ozuru, Shohei Wakao, Takahiro Tsuji, Naoya Ohara, Takashi Matsuba, Muhammad Y. Amuran, Junko Isobe, Morio Iino, Naoki Nishida, Sari Matsumoto, Kimiharu Iwadate, Noriko Konishi, Kaori Yasuda, Kosuke Tashiro, Misato Hida, Arisato Yadoiwa, Shinsuke Kato, Eijiro Yamashita, Sohkichi Matsumoto, Yoichi Kurozawa, Mari Dezawa, and Jun Fujii

Supplemental Information

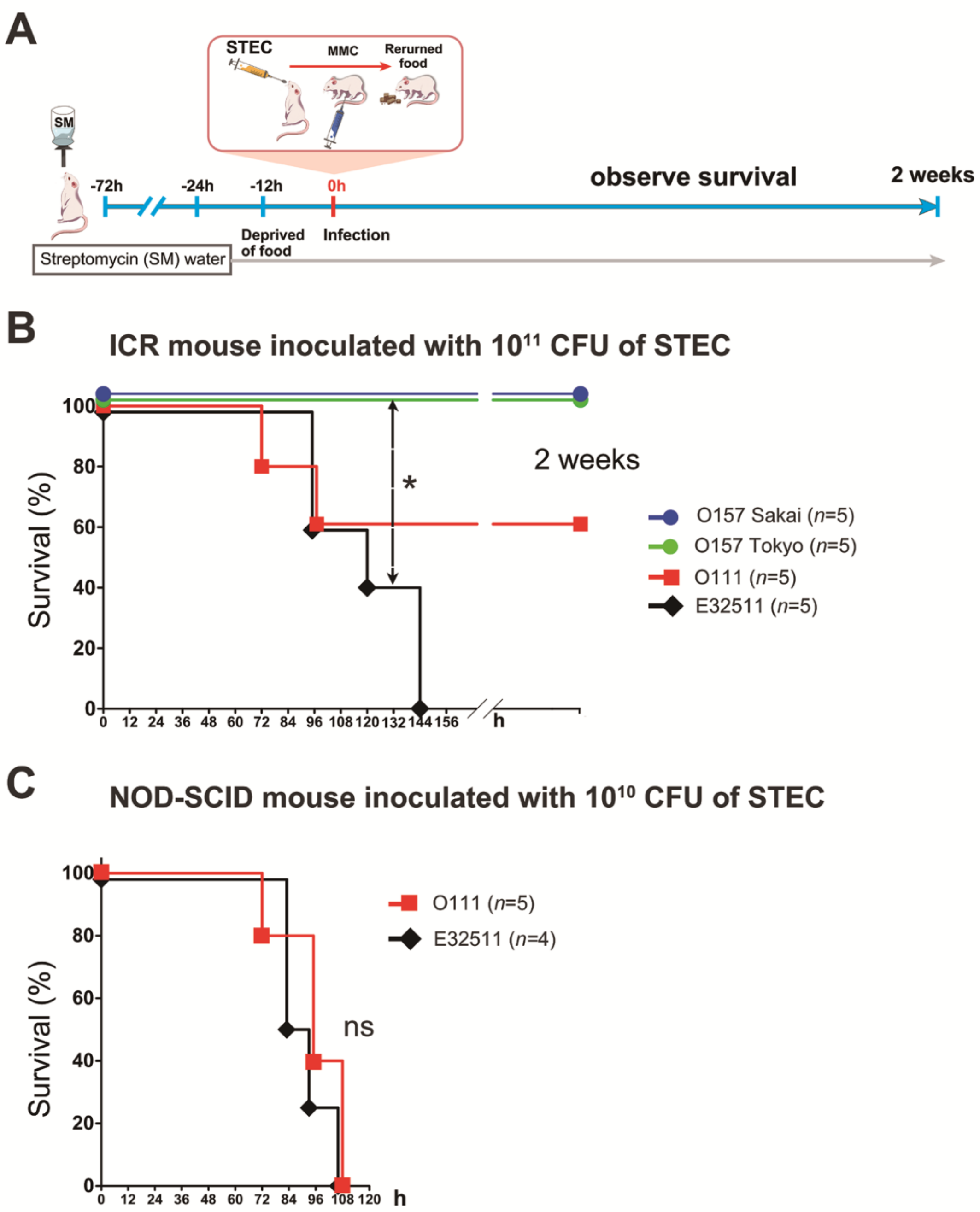

Figure S1. Selection of strains and dose of STEC.

(**A**) Experimental protocol for selecting strains and does of STEC. (B) Selection of STEC among four candidate strains is done by ICR mice. Blue, green, red, and black lines indicate STEC O157 Sakai (*n* = 5), STEC O157 Tokyo (*n* = 5), STEC O111 (*n* = 5), and E32511 (*n* = 5), respectively. * $P < 0.05$. (**B**) Since 1×10^9 CFU-O111 and-E32511 did not kill any of mice, 1×10^{10} CFU was tested in NOD-SCID mice. All mice were killed by 108 h after oral inoculation of 1×10^{10} CFU-O111 and-E32511. ns, not significant. (**C**) Survival curves of O111 and E32511 administered at 1×10^{10} CFU to NOD-SCID mice. For the survival curves of NOD/SCID mice, red and black lines indicate STEC O111 (n = 5) and E32511 (n = 4), respectively. ns, not significant.

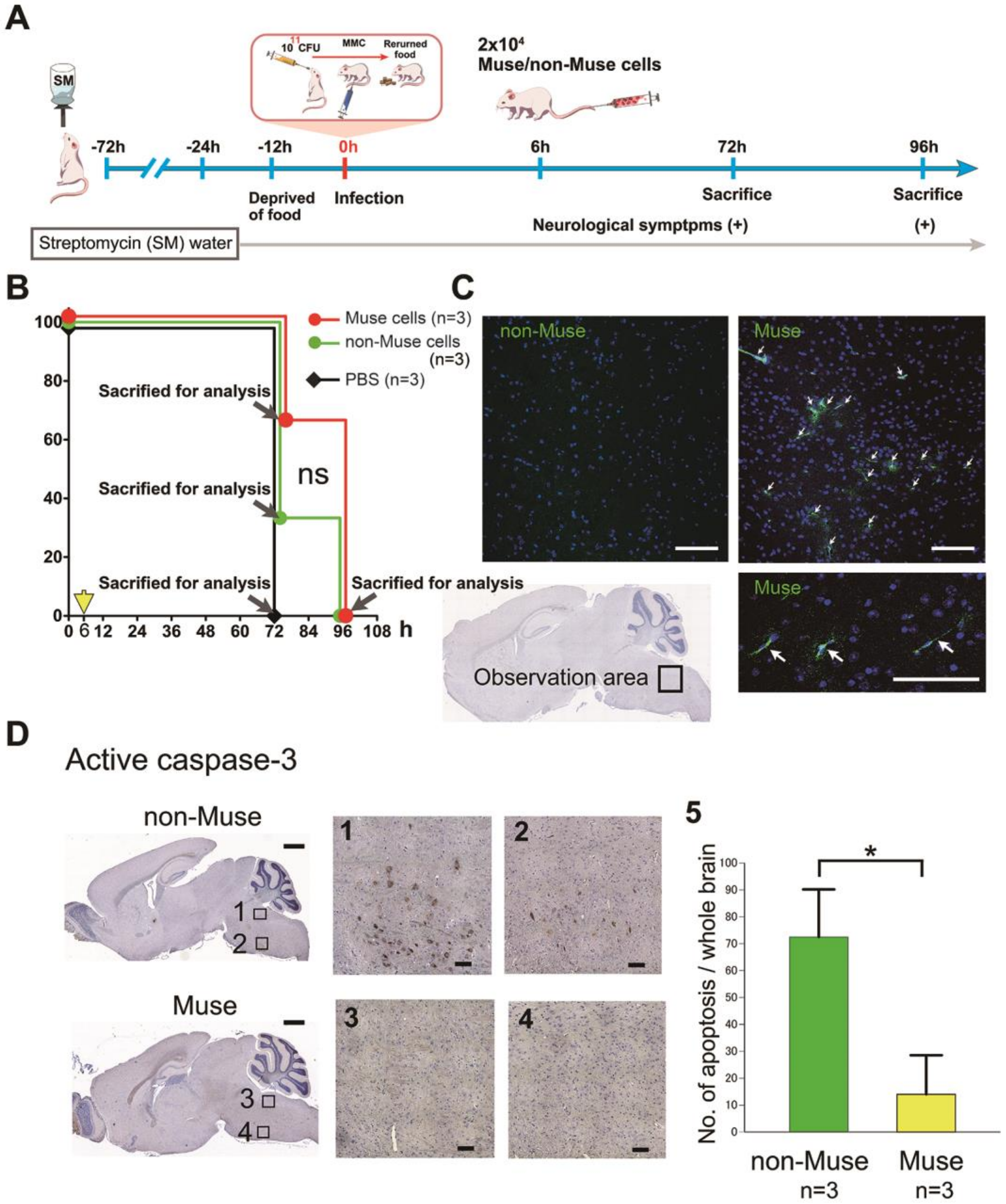

Figure S2. Migration of intravenously injected Muse/non-Muse cell to the brain and expression of caspase-3 in the brain of E32511-infected NOD-SCID. See Additional Material and Methods, References.

(A) Experimental protocol of the mouse model infected E32511. **(B)** Survival rate of $2x10^4$ Muse and non-Muse cell-injected NOD-SCID mice infected with E32511. Yellow arrow indicates the timing (6 h) of intravenous administrations after E32511 infection. Mice reached the endpoint of some symptoms including weakness, weight loss, marked loss of strength, loss of pain sense, flaccid paralysis, and backbone deformity at 72 h in the PBS control, and at 96 h in Muse and non-Muse cell treatment groups. Mice were then sacrificed for immunohistochemistry. **(C)** Engraftment of Muse/non-Muse cells. The

observation area is shown in the whole mouse brain. Alexa Fluor 488-non-Muse cells were scarcely detected, while a number of Alexa Fluor 488-Muse cells (white arrows) were observed. High magnification image is provided for the Muse group. Scale bar=50 µm. (**D**) Activated caspase-3 was detected in the reticular formation of the medulla oblongata (box **1**) and pons (box **2**) in the non-Muse cell-treated brain, but not in the Muse cell-treated brain (boxes **3** and **4**). Caspase 3+ cells, indicating apoptosis, were significantly decreased in the Muse cell group compared with the non-Muse cell group (**5, graph**). *$p < 0.05$. Scale bars; whole brains =1 mm, histological sections=100 µm (inset).

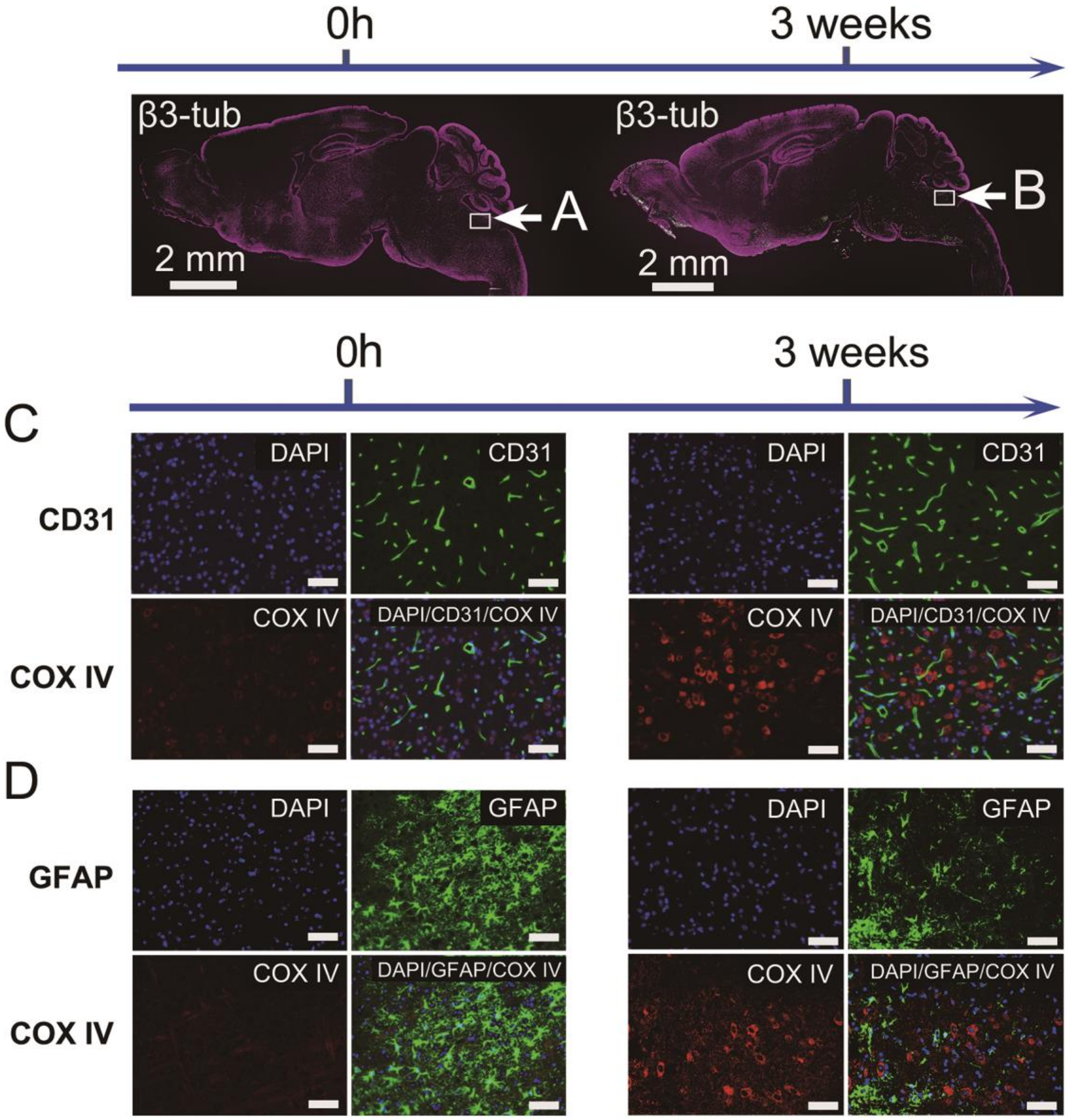

Figure S3. Immunohistochemical analysis for engrafted Muse cells in the O111-infected NOD-SCID mice

Control (0 h) and 3 weeks after intravenous injection of $5x10^4$ Muse cells. White boxes A and B is areas subjected to immunohistochemistry. No cytochrome c oxidase (COXIV) immunoreactivity (red) was observed in the reticular formation of the medulla oblongata before injection of Muse cells as the control. At 3 weeks after, COXIV immunoreactivity was observed in mice injected with Muse cells in the same area. **(C)** Double staining of COXIV (engrafted red cells) and CD31 (green cells) in control mice (scale bar: 50 µm, as described below). (**D**) Double staining of COXIV and glial fibrillary acidic protein (GFAP) (green cells).

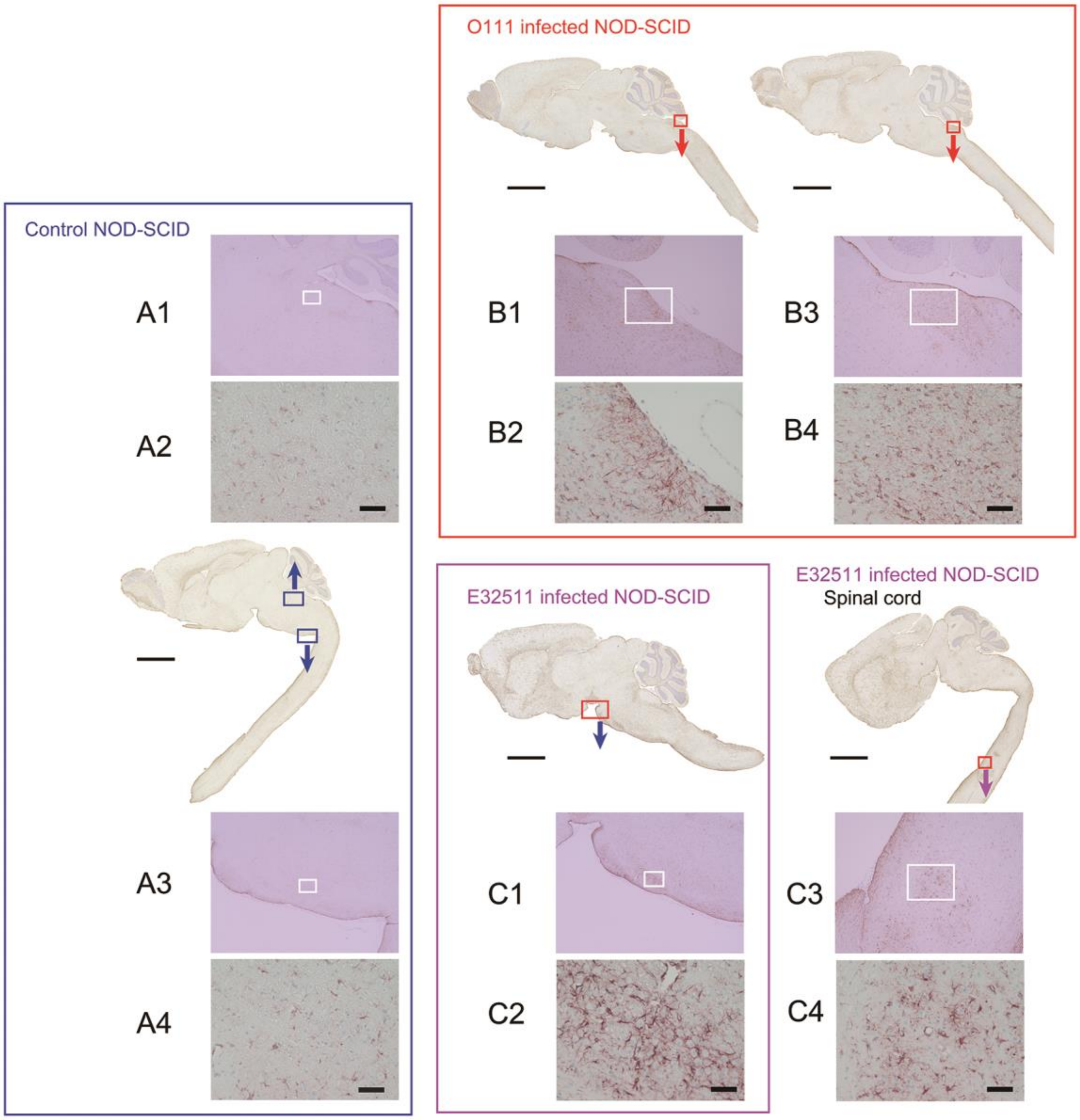

Figure S4. Immunohistochemistry for GFAP(+) reactive astrocytes in O111- and E32511-infected NOD-SCID mice

(**A1-A4)** The brain and spinal cord of control mouse without injection did not show GFAP+ reactive astrocytes. (**B1-B4**) O111 and (**C1-C4**) E32511 at 1×10^{10} colony forming units (CFU) infected mice at 96 h after inoculation showed strong GFAP(+) reactive astrocytes. Scale bars, low magnification=2 mm. High magnification=50 µm

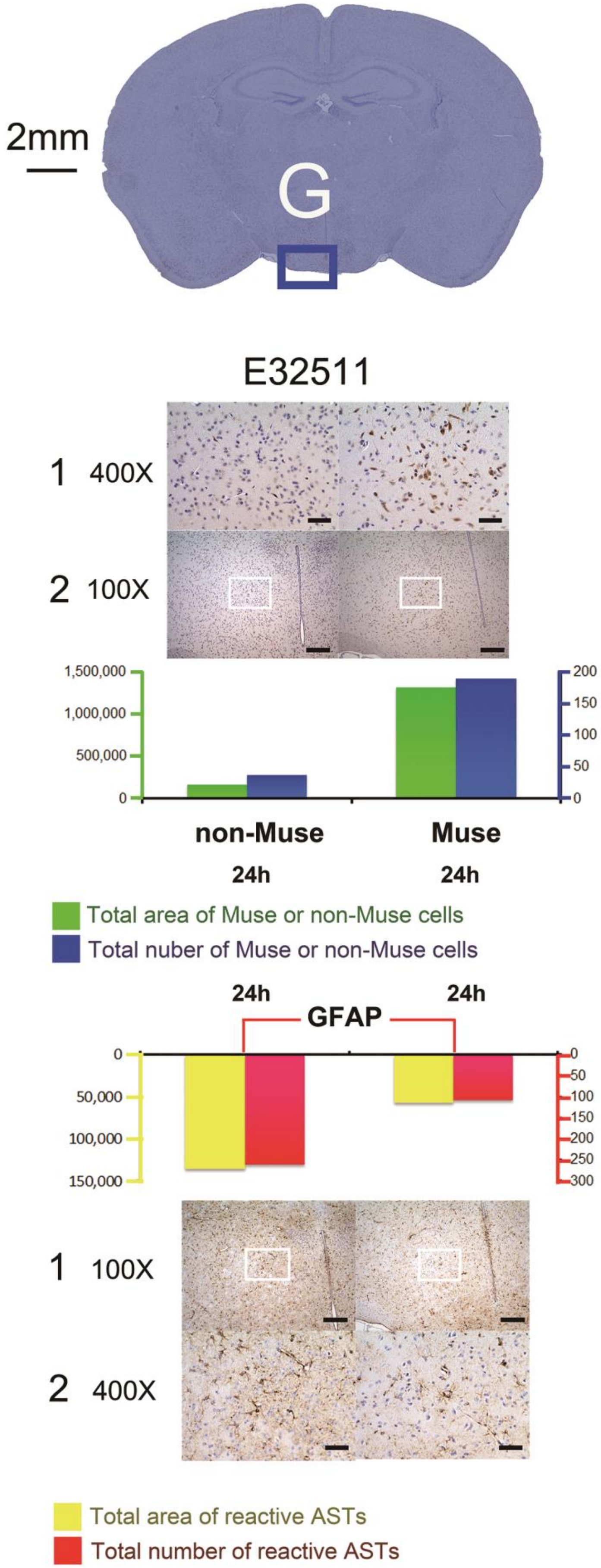
2mm
G
E32511
1 400X
2 100X
1,500,000
1,000,000
500,000
0
200
150
100
50
0
non-Muse
Muse
24h
24h
Total area of Muse or non-Muse cells
Total nuber of Muse or non-Muse cells
24h
GFAP
24h
0
50,000
100,000
150,000
0
50
100
150
200
250
300
1 100X
2 400X
Total area of reactive ASTs
Total number of reactive ASTs

Figure S5. Engraftment of intravenously injected Muse/non-Muse cells into NOD-SCID mouse brains and rapid suppression of reactive astrocytes following O157 infection at 24 h

Blue box in (G) shows the observation area in the right thalamic ventral nuclei of the coronal section. Immunohistochemistry and the area and number of COXIV- and GFAP-positive cells shown in the similar manner to Figure 5. Scale bars high magnification=50 μm and low magnification= 200 μm.

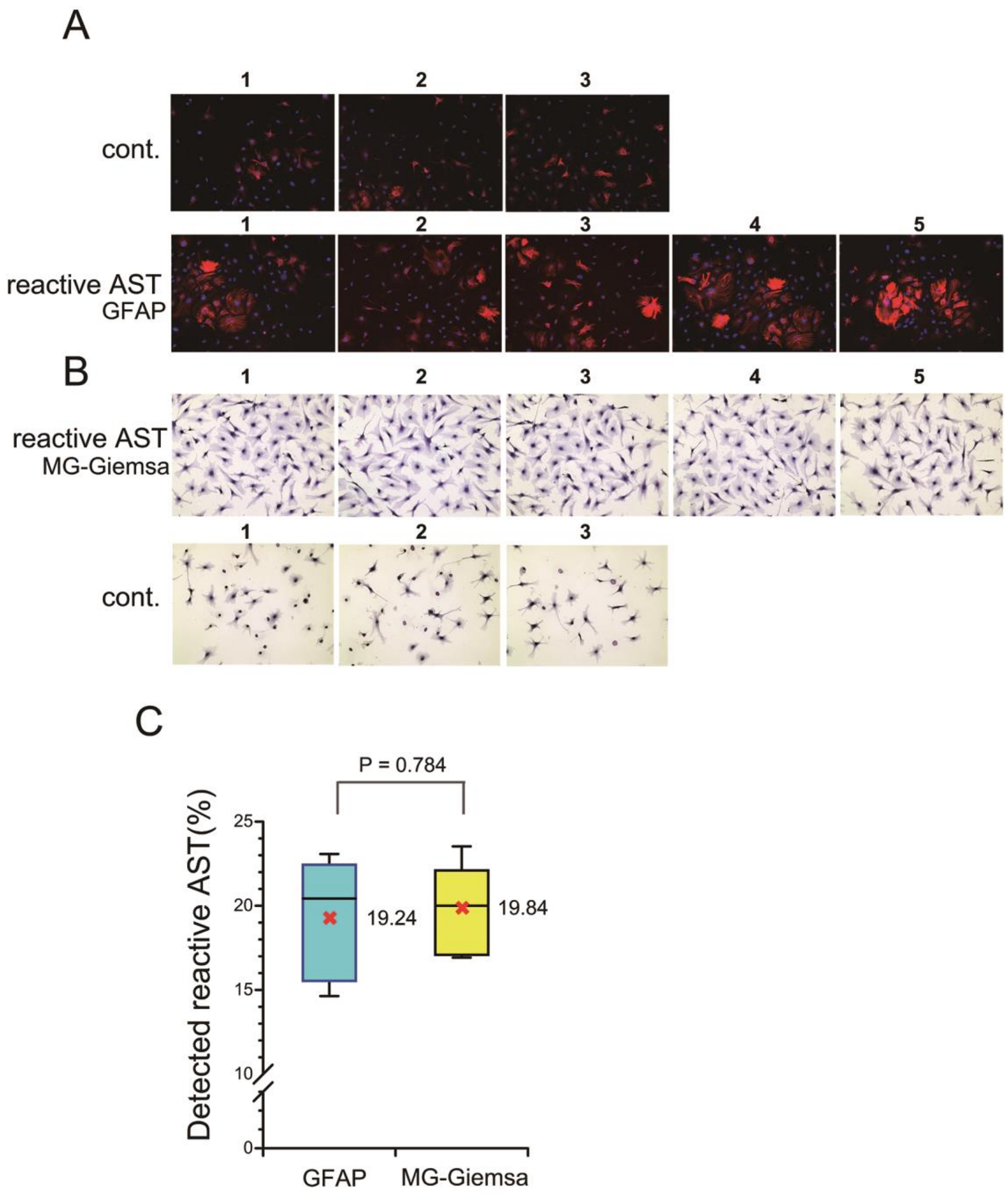

Figure S6. Comparison of May-Grunwald-Giemsa and immunohistochemistry of glial fibrillary acidic protein (GFAP) staining reactive astrocyte.

A. Three fields of GFAP stained control astrocytes (AST). The GFAP staining of five fields indicates reactive AST.

B. May-Grunwald-Giemsa (MG-Giemsa) staining of five fields indicates reactive AST. MG-Giemsa staining indicates control AST in three fields.

C. Reactive AST detected by GFAP and MG-Giemsa staining. GFAP staining reactive AST and total ASTs were counted. MG-Giemsa counts indicated four-fold higher MG-Giemsa staining for AST and total AST in (B). The mean percentages of GFAP and MG- Giemsa stained cells were 19.24 and 19.84, respectively. The box shows the 25th–75th percentiles, whereas the whiskers indicate the 10th and 90th percentiles. No significant difference was determined between GFAP and MG-Giemsa by independent t-test (P=0.784).

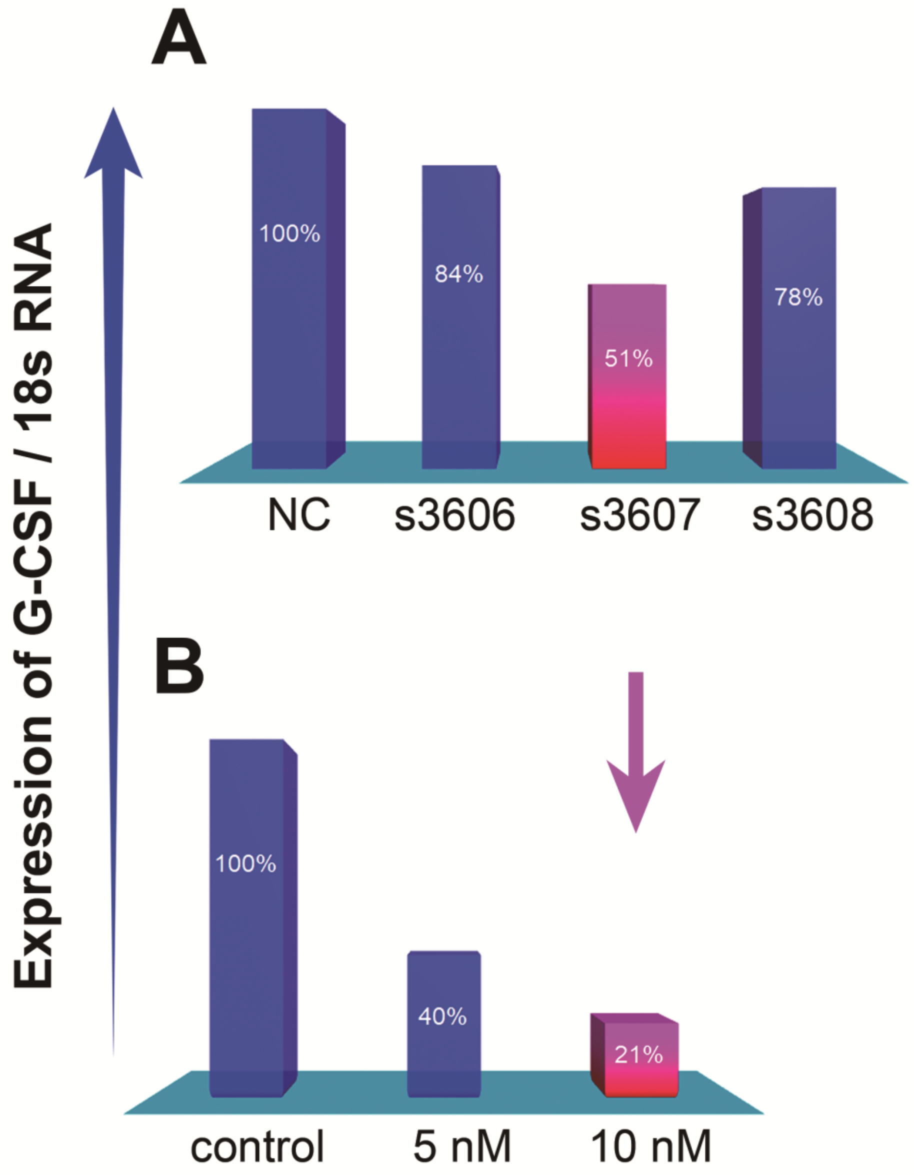

Figure S7. Preparation of siRNAs.

(**A**) Comparison of three commercial siRNAs (s3606, s3607, and s3608) and non-targeting control (NC) siRNA. The upper graph shows expression of G-CSF in Muse cells normalized to the 18S rRNA endogenous control. (**B**) Relative expression of G-CSF mRNA in cells treated with 5 and 10 nM siRNAs compared with NC as measured by digital PCR.

Additional Material and Methods, References

Ethics statement.

Animal experiments were carried out in strict accordance with the recommendations set forth in the Guidelines for Proper Conduct of Animal Experiments formulated by the Science Council of Japan. The protocol was approved by the Committee on the Ethics of Animal Experiments of Kyushu University, Japan (Permit Number: A23-141-1). All surgeries were performed under sevoflurane anesthesia, and all efforts were made to minimize suffering.

Bacterial strains

The Stx2c-producing *E. coli* O157:H− strain E32511/HSC was used in this study. Streptomycin (SM)- and mitomycin C (MMC)-resistant E32511/HSC was grown in nutrient agar supplemented with 100 μg/mL SM (Wako Pure Chemical Industries, Ltd., Osaka, Japan) and 0.5 μg/mL MMC (Kyowa Hakko Kogyo Co. Ltd., Tokyo, Japan).

Animals

Seven-week-old male NOD-SCID mice were purchased from Charles River laboratories Japan, Inc.

They were housed in cages with a 12-h light-dark cycle, and were given free access to laboratory food and distilled water.

Preparation of Muse and non-Muse cells

Muse and non-Muse cells were prepared from adult human bone marrow stromal cells, as described previously [1].

Muse cell transplantation

In this study, we used a mouse model with E32511/HSC given orally and MMC given i.p. [2]. Briefly, all mice were given SM-containing drinking water (5 g/L) *ad libitum* to reduce normal intestinal flora. On day 3 of SM treatment, all the mice were completely deprived of food for 12 h until bacterial inoculation. Each mouse was orogastrically inoculated with 0.5 mL of bacterial suspension using a syringe attached to a sterile disposable feeding needle (diameter, 1.9 mm; length, 38 mm, Fuchigami Co. Ltd., Nara, Japan) passed into the stomach. Simultaneously, they were injected i.p. with 2.5 μg/g MMC. In this study, the bacterial suspension of E32511 was adjusted to 1×10^{11} CFU in PBS and caused 100% death. The mice were transplanted with 2.0×10^{4} cells per mouse of Muse (n = 3) or non-Muse (n = 3) cells intravenously via the tail vein 6 h after

bacterial inoculation. After the completion of treatment, animals were returned to their respective housing and given food and SM-containing drinking water (5 g/L) *ad libitum*. The brain was harvested on at 72h and 96h after the E32511 inoculation.

Immunohistochemistry

E32511 oral infection was performed as described above. The mice were injected with 2×104 Muse (n=3) or non-Muse (n=3) cells intravenously per mouse via the tail vein at 6 h after E32511 inoculation. After completion of the treatments, mice were housed according to the treatment groups and provided with food and SM-containing drinking water (5 g/L) ad libitum. The NOD-SCID mice developed some symptoms including weakness, marked loss of strength, weight loss, backbone deformity, loss of pain sense, and flaccid paralysis of the extremities. The mice were then sacrificed for immunohistochemical analysis for detecting Muse cells in the mouse brain. Mice were anesthetized with sevoflurane (Maruishi Pharmaceutical Co., Ltd. Osaka, Japan). Subsequently, the mice were perfused with 10 ml PBS through the left cardiac ventricle, followed by 10 ml of 4% paraformaldehyde (Wako Laboratory Chemicals, Osaka, Japan)/PBS to fix their organ tissues. The brain and spinal cord were harvested and fixed in 4% PFA/PBS. Then, the brain and spinal cord were processed for immunohistochemical examination. At 68 h after injection, the animals were deeply anesthetized with 4% isoflurane in N_2O:O_2 (70:30) and transcardially perfused with 4% paraformaldehyde. The brain was removed and immersed in 4% paraformaldehyde for another 2 days, followed by preparation of 10 μm-thick cryosections. They were then incubated in blocking solution and reacted with a primary antibody against human mitochondria (mouse IgG, 1:100, Abcam) to detect human Muse or non-Muse cells. Samples were further incubated with anti-mouse IgG conjugated with Alexa Fluor 488 IgG (Invitrogen; green) as the secondary antibody to detect the human Muse or non-Muse cells and counterstained with DAPI (Invitrogen; blue; all nuclei) because NOD-SCID mouse has no mouse IgG. The samples were examined by a c1si confocal microscope system (Nikon, Tokyo, Japan).

For detecting active-caspase3, formalin-fixed, paraffin-embedded tissue blocks were cut into 3 μm-thick sections. The sections were deparaffinized in xylene, dehydrated in an ethanol gradient, and pretreated for heat-based antigen retrieval in 10 nmol/L citrate buffer solution (pH 6) for 20 min in an autoclave. The sections were incubated with 5% dry nonfat milk to eliminate nonspecific binding of the antibodies and then incubated overnight at 4°C with a polyclonal anti-cleaved caspase-3

antibody (1:400, Cell Signaling Technology, Inc, Beverly, MA, USA) diluted in PBS containing 5% BSA and 0.1% Tween 20. After endogenous peroxidase activity was blocked with methanol containing 1% H_2O_2, immunostaining of anti-cleaved caspase-3 was performed. Then, the sections were incubated with an HRP-labeled anti-rabbit secondary antibody, HistoFine (Dako, Glostrup, Denmark), for 30 min. Target proteins were visualized with a peroxidase stain DAB kit (Nacalai Tesque, Kyoto, Japan). Nuclei were counterstained with hematoxylin. Specimens were examined under a BZ-9000E HS All-in-one Fluorescence Microscope (Keyence, Osaka, Japan).

References

1. Kuroda, Y, Wakao, S, Kitada, M, Murakami, T, Nojima, M, and Dezawa, M (2013). Isolation, culture and evaluation of multilineage-differentiating stress-enduring (Muse) cells. *Nat Protoc* **8**: 1391-1415.

2. Fujii, J, Kita, T, Yoshida, S, Takeda, T, Kobayashi, H, Tanaka, N, *et al.* (1994). Direct evidence of neuron impairment by oral infection with verotoxin-producing Escherichia coli O157:H- in mitomycin-treated mice. *Infect Immun* **62**: 3447-3453.

Supplemental Table

Table S1 6h the ratio of Muse and non-Muse cells

Upregulated genes are shown in pink, and downregulated genes are shown in blue. G-CFF is shown as yellow.

ProbeID	GeneSymbol	PrimaryAccession	Comparison 1	Comparison 2	Comparison 3	Comparison 4
A_33_P3337272	NRARP	NM_001004354	30.46589723	26.36424822	21.37645776	18.49852753
A_23_P102611	WISP2	NM_003881	16.17048036	17.5602088	14.43979611	15.68078555
A_24_P250227	NR1D1	NM_021724	12.51485461	13.31318229	12.08569229	12.85664353
A_23_P54996	TEX14	NM_198393	11.95824614	13.26590357	11.90994827	13.21232423
A_23_P339818	ARRDC4	NM_183376	10.47889531	10.11861914	11.07924141	10.69832466
A_23_P137665	CHI3L1	NM_001276	10.81740072	10.80716507	10.37768263	10.36786305
A_32_P74409	C11orf96	NM_001145033	3.663213845	3.947149778	3.292583698	3.547792065
A_22_P00000694		ENST00000553791	27.74693879	31.95563701	17.32927518	19.95780621
A_23_P410717	CIART	NM_144697	11.41555478	10.59073471	12.11774059	11.24218475
A_21_P0011967	SFRP4	NM_003014	17.21555235	14.63867466	11.09395475	9.433376928
A_23_P150549	PGA3	NM_001079807	15.12210321	16.61263623	15.02227662	16.50297009
A_21_P0014143	LINC01511	NR_125810	9.784466652	11.23349701	13.80603702	15.85064174
A_22_P00014004		ENST00000568143	22.41668066	21.06721175	12.84937484	12.07585122
A_23_P150609	IGF2	NM_000612	7.769633638	8.203523011	6.427600455	6.78654499
A_23_P29257	H1F0	NM_005318	7.762627337	8.262167243	7.34770681	7.820545788
A_33_P6475164	LINC01447	NR_108090	9.75395827	9.713558076	7.942489446	7.909592226
A_23_P151975	RHCG	NM_016321	8.107189873	9.013048147	6.118102363	6.801709598
A_33_P3359973		ENST00000436500	14.14110018	13.18038914	11.92582317	11.11561252
A_23_P98350	BIRC3	NM_001165	7.547832847	7.251131122	7.043932137	6.76703851
A_33_P3236392	PVRL4	NM_030916	13.73394628	14.67204793	11.83584492	12.64429614
A_23_P139500	BHLHE41	NM_030762	7.831601604	7.18483546	8.106671674	7.437189102
A_21_P0008282	LINC01467	NR_110074	13.41652707	11.94151769	14.43289902	12.84614999
A_33_P3300591			19.87913214	20.84462577	10.50720692	11.01752303
A_33_P3718734	LOC729083	NR_122070	7.475782519	7.380004534	6.605831284	6.521198911
A_33_P3229241	HIST2H2BF	ENST00000369167	7.463970898	7.72687634	7.777343464	8.051286911
A_19_P00320881	LINC01539	NR_040025	8.713446519	8.563331211	6.813808455	6.696420122
A_24_P887857		ENST00000420566	8.531616116	9.866326835	7.557903105	8.740283342
A_23_P205046	ANKRD10	NM_017664	8.516832431	6.939612898	9.08123873	7.399497634

A_23_P368909	LINC00346	NR_027701	7.262879345	7.247493418	6.492554684	6.478800639
A_24_P260639	HIST1H1D	NM_005320	7.253043446	7.576643395	7.413690351	7.744457681
A_33_P3366127	RFPL4AL1	NM_001277397	12.25824309	13.74972905	17.37747918	19.49183326
A_33_P3345534	KRT14	NM_000526	8.28624896	9.814191898	7.823673008	9.266319249
A_22_P00015968		ENST00000522960	11.9395736	12.23919705	8.953884135	9.178581746
A_23_P15101	TMC5	NM_024780	11.90417959	11.68421943	9.671201707	9.49250153
A_32_P62963	KRT16P2	NR_029392	8.145855206	9.621816243	6.593706861	7.788431561
A_23_P93169	LGSN	NM_016571	16.82507163	14.64375669	33.13544769	28.8395464
A_23_P377839	LINC00161	NR_026552	7.919145744	7.361244903	7.366574569	6.847602161
A_23_P151059	FAM90A1	NM_018088	11.3608873	12.49334604	18.84234271	20.72055655
A_22_P00007695	lnc-HIST2H2AA3-1	lnc-HIST2H2AA3-1:1	6.772700645	6.716878524	6.292715948	6.240849969
A_21_P0005616	LINC01419	NR_122034	11.3080134	9.887546128	9.535287536	8.337502973
A_22_P00022297		ENST00000521681	16.39971644	16.2608407	12.25023197	12.1464948
A_23_P50368	OSCAR	NM_206818	6.70584885	6.527404223	6.242175471	6.076069331
A_32_P76627		ENST00000417510	7.743858227	8.714556579	7.803248827	8.781391835
A_33_P3229122	HIST1H2BF	NM_003522	7.623869023	9.617649731	5.991670399	7.558601417
A_23_P2705	LPAR6	NM_005767	7.593186039	6.341635135	5.063094781	4.228567507
A_23_P18672	GBA3	NM_020973	15.83446362	13.50960253	19.66953868	16.78160093
A_23_P131676	ACKR3	NM_020311	6.547005718	5.110378227	5.76118686	4.496993764
A_33_P3839897	RNU4ATAC	DW419002	7.523569464	7.2998347	6.934420373	6.728205634
A_33_P3795649		ENST00000447535	10.76028773	11.4938514	11.00852913	11.75901623
A_24_P623782	FRG2	NM_001005217	15.60187079	12.4667238	13.52123219	10.80418301
A_21_P0009266		ENST00000612725	15.39677392	16.00465268	17.71667557	18.41614617
A_23_P17103	TSGA10	NM_025244	10.48937431	10.59523141	10.06593941	10.16752327
A_32_P145867	OCM2	NM_006188	15.10358534	15.14932848	15.3012102	15.34755187
A_33_P3341722		CU692082	5.375470323	5.60082922	5.07011581	5.282673157
A_22_P00010915		ENST00000512831	14.88231578	15.6258096	13.59766745	14.27698254
A_32_P128209	LINC01554	NR_026936	7.228879236	7.281043607	7.712725545	7.768381404
A_21_P0009764		ENST00000592431	7.156075323	7.299805897	6.426748163	6.555830119
A_33_P3298159	PTGDS	NM_000954	7.124644969	7.526418084	5.685530322	6.006148857
A_33_P3352522	GAS6-AS1	AK092862	14.24914428	13.46595265	10.82349286	10.22858912
A_33_P3298216	MYO16	NM_001198950	9.85662034	9.578566617	11.61699784	11.28928414
A_33_P3375790	RFPL4AL1	NM_001277397	9.846755896	10.98427303	14.06262565	15.68716857
A_24_P55148	HIST1H2BJ	NM_021058	6.062468671	6.265208589	6.066415308	6.269287209

A_23_P89589	PER1	NM_002616	6.05454715	6.275082996	6.23835856	6.465589705
A_23_P381368	HOXD10	NM_002148	9.80357631	9.073289331	8.443951514	7.814945562
A_33_P3354151	BOLA1	NM_016074	6.037751912	6.712327403	6.897055291	7.667637542
A_23_P112554	COL15A1	NM_001855	9.706628106	9.22077459	7.425402478	7.053732948
A_22_P00014506		ENST00000420865	9.673324724	9.629430088	12.21005965	12.15465408
A_33_P3331831		ENST00000612156	9.650464546	10.20461304	7.971419394	8.429153841
A_23_P365738	ARC	NM_015193	5.636960299	5.995795204	4.736541664	5.038058153
A_19_P00803685	ANKRD33B	NM_001164440	5.927655862	5.708423948	6.161603542	5.933719169
A_23_P167997	HIST1H2BG	NM_003518	5.918917398	6.004694178	5.826861402	5.91130411
A_23_P42868	IGFBP1	NM_000596	5.904763582	5.984512459	5.496734088	5.570972178
A_21_P0011517	KRT14	NM_000526	6.760431206	8.312404159	7.079927136	8.70524586
A_33_P3215640	PI16	NM_153370	6.757035605	6.924074317	5.575104415	5.71292495
A_21_P0005727	LINC01111	NR_105006	6.748066977	6.154089317	6.101790489	5.564699312
A_23_P321501	DHRS2	NM_182908	5.565621779	5.883874675	5.270979892	5.572384601
A_23_P323685	HIST1H4H	NM_003543	4.968133633	4.743607577	4.02974687	3.847629552
A_21_P0000795	LINC00624	NR_038423	9.197693132	9.624150735	9.830786283	10.2865977
A_24_P10137	RGCC	NM_014059	6.562336047	6.262024052	5.673336897	5.413708144
A_24_P62783	FABP3	NM_004102	12.94269659	12.45816521	10.22011175	9.837504865
A_33_P3229301	DKFZp434J0226	NR_027003	12.89867598	14.26821608	8.486355455	9.387409494
A_23_P216225	EGR3	NM_004430	6.51718986	5.626565703	6.062441989	5.233962629
A_21_P0014444	LOC100507639	NR_121625	12.80839266	12.58009056	15.67981403	15.40033053
A_23_P145569	SLC22A1	NM_153187	6.478937629	7.265863671	5.94807027	6.670517632
A_21_P0007899	LOC100996671	NR_110036	9.016533995	9.775735077	6.571160798	7.124459038
A_24_P229234	MYO16	NM_015011	9.01101957	8.136747429	11.65563743	10.5247777
A_21_P0014172	HIST1H4H	BC010926	4.869541002	4.914148186	4.726038831	4.769331471
A_23_P96087	H1FX	NM_006026	5.6436035	5.989681108	5.41079563	5.742596972
A_23_P500353	KCNN2	NM_021614	12.66656075	10.26695844	9.65144056	7.82303429
A_33_P3362311	C6orf222	NM_001010903	8.916201869	9.600733045	6.569724631	7.074107708
A_22_P00012795	LINC01419	NR_122034	8.869082825	7.799573956	12.51407296	11.00502041
A_33_P3214105	ATF3	NM_001674	5.282965839	5.012177982	4.968909741	4.714219391
A_22_P00016430		ENST00000554254	5.27439377	5.560179764	5.775935049	6.088896389
A_33_P3265394	WDR74	ENST00000538098	8.752846683	11.59943103	7.676771511	10.17339671
A_23_P307310	ACAN	NM_013227	8.736307813	10.39239887	6.584677611	7.832896649
A_33_P3229246	HIST2H2BE	NM_003528	6.299498659	6.775629539	5.444614235	5.856130945
A_23_P46936	EGR2	NM_000399	4.76594555	4.665648534	4.699847527	4.600941512

A_23_P70448	HIST1H1A	NM_005325	5.492390807	6.126468819	5.203680241	5.804427591
A_23_P326760	MYRIP	NM_015460	12.0997394	11.65377774	19.70471563	18.97845638
A_22_P00015892	lnc-TCF19-1	lnc-TCF19-1:4	8.609321475	9.445178281	9.190641748	10.08293744
A_24_P33895	ATF3	NM_001040619	5.119195459	5.075617087	4.802473333	4.761591134
A_32_P33083	VCX2	NM_016378	11.7963996	12.38786188	24.90546726	26.15420797
A_23_P97112	SELE	NM_000450	5.36623606	5.720430167	5.408023901	5.76497618
A_23_P59045	HIST1H2AE	NM_021052	5.067011427	5.451303748	4.19672999	4.515018419
A_24_P273489	SH2D4B	NM_207372	11.61397866	10.80646431	13.71609834	12.76242462
A_23_P350005	TRIML2	NM_173553	5.931018178	5.455113678	6.368486893	5.857479933
A_23_P167017	POPDC2	NM_022135	5.927760485	5.248229082	6.02106781	5.330840081
A_24_P241183	CLEC2D	NM_013269	5.924373767	6.232094016	5.60931671	5.900672455
A_33_P3839820	LOC101928538	ENST00000589708	11.26704828	10.57206396	8.421849899	7.902365696
A_23_P216966	PTGS1	NM_000962	5.899915512	6.520558864	6.120098839	6.763904441
A_21_P0006028	TCERG1L-AS1	NR_120623	5.89844769	5.504935648	4.695506338	4.382247937
A_23_P97700	TXNIP	NM_006472	4.491833093	4.223152033	5.075937981	4.772318418
A_19_P00317789	LOC93622	NR_015433	5.137746375	5.589153115	4.98217236	5.419910236
A_33_P3344229	HIST1H4A	NM_003538	4.883517791	4.895486895	3.887242356	3.89676967
A_33_P3692979	LOC283485	AK093862	7.936959963	7.957247686	10.54105919	10.56800328
A_33_P3410836	HIST1H4D	NM_003539	4.809481179	4.569842246	4.050156107	3.848351576
A_22_P00000845	CRHBP	ENST00000514258	10.76888973	10.7651835	9.09041597	9.087287402
A_33_P3223592	APOE	NM_001302688	5.050400371	5.276272263	4.033263328	4.213645229
A_33_P3361707	MDM2	NM_002392	5.047189086	4.482753774	4.903743369	4.355349822
A_23_P17065	CCL20	NM_004591	4.367995018	4.297289567	4.203376522	4.135335778
A_23_P359540	HIST1H4F	NM_003540	4.766454402	4.729162978	3.14717899	3.12255633
A_24_P20873	HIST1H4I	NM_003495	4.735084185	4.637886493	3.295106189	3.227467114
A_22_P00006266		ENST00000449453	7.63477536	9.288097429	4.102275743	4.990629715
A_23_P25396	NR1H4	NM_005123	7.624211484	7.046791912	12.9988717	12.0144023
A_33_P3229239	HIST2H2BF	ENST00000369167	5.601881756	5.999842971	6.422855913	6.87913965
A_22_P00025133		ENST00000454307	10.44137684	9.627754777	15.01706229	13.84688968
A_33_P3262376	OTUD7A	NM_130901	5.588395041	5.484648937	4.550656727	4.46617578
A_33_P3881812	IRGM	ENST00000520549	5.577630054	5.779252724	4.677595314	4.846683125
A_23_P49643	GRAP	NM_006613	10.35721599	14.17586953	9.640647278	13.19510554
A_21_P0013007	LOC643201	ENST00000510155	7.53110917	7.350795909	5.996810175	5.853231803
A_33_P3287879	HIST1H3H	NM_003536	4.911505492	5.064361866	3.903176988	4.024652059
A_23_P106002	NFKBIA	NM_020529	2.466423309	2.450517917	2.356768547	2.341570293

A_24_P414658	HIST1H2AG	ENST00000359193	7.473757899	6.899198721	8.373712981	7.729968065
A_23_P355439	HIST1H2AA	NM_170745	5.516302958	5.613838755	4.847902269	4.933619826
A_33_P3299865	HIST1H4K	NM_003541	4.264481664	4.288640214	3.089990896	3.107495884
A_23_P215454	ELN	NM_001278939	7.458767977	7.603467884	5.701499787	5.812108736
A_22_P00006427	LOC101929181	NR_104624	7.419860259	7.985655014	7.924190689	8.528442707
A_23_P134237	RARRES2	NM_002889	10.10157917	11.92617259	11.4566651	13.52602033
A_24_P788878	C2CD4B	NM_001007595	7.377156725	7.75948941	7.524805479	7.914790293
A_33_P3335910	SYNE1	NM_033071	5.457186987	5.954534074	3.941714352	4.300947078
A_21_P0010596	RNF223	ENST00000453464	7.334206592	8.839725746	9.75366423	11.75583422
A_24_P882732		ENST00000300992	5.430835143	5.935404067	5.823038447	6.36404626
A_33_P3324974			7.323762887	7.520203493	5.940935644	6.100285559
A_23_P30799	HIST1H3F	NM_021018	4.569052734	5.005483272	4.208933505	4.610965878
A_33_P3347320	ADIRF-AS1	BC017676	7.242743504	7.286428407	6.447780873	6.486670927
A_21_P0006440		ENST00000438499	9.867776718	10.57730429	5.150763466	5.52112133
A_23_P149545	HIST2H2BE	NM_003528	5.371802914	5.761883826	6.3689057	6.831392612
A_22_P00021404		ENST00000558010	9.782866893	9.213568572	10.88466535	10.25124963
A_23_P7976	HIST1H1E	NM_005321	4.503182614	4.823186639	4.562753022	4.886990224
A_23_P9614	NDUFA4L2	NM_020142	7.139141145	7.665024233	6.760512385	7.258504939
A_24_P64167	PTGS1	NM_000962	9.675081066	9.906680251	10.43031757	10.67999538
A_24_P146211	HIST1H2BD	NM_021063	4.457268886	4.247454774	4.375443301	4.169480911
A_33_P3229083	HIST1H2BK	NM_080593	4.455414847	4.441046285	4.331663627	4.317694158
A_21_P0008216	lnc-ANKRD10-1	lnc-ANKRD10-1:1	9.569514118	10.29187892	6.200432739	6.668478903
A_23_P502750	MDM2	NM_002392	5.252214756	4.823768209	4.681843026	4.299924242
A_23_P167983	HIST1H2AC	ENST00000314088	4.447379127	4.125310017	4.88615091	4.532306943
A_33_P3397323	ZNF441	NM_152355	4.662197169	4.691130661	5.567224099	5.601774168
A_23_P62901	BTG2	NM_006763	4.446218079	4.549100141	4.636795658	4.744087539
A_21_P0012685	LOC100507639	NR_121625	9.553178932	9.272479909	13.54549547	13.14749106
A_21_P0013154		ENST00000417483	5.238339506	4.940185774	4.923765691	4.643516747
A_23_P51126	IL1RL1	NM_016232	5.234376778	5.011241136	4.499194396	4.307398758
A_23_P125686	INGX	NR_002226	9.519770876	10.48266991	6.972548046	7.67780239
A_23_P93180	HIST1H2BC	NM_003526	4.636871348	4.337913841	4.902625495	4.586533763
A_23_P421379	IGF2	NM_000612	9.441397127	9.858793291	8.556170775	8.934431832
A_23_P415411	HIST1H4E	NM_003545	4.393818588	4.705654949	3.648785403	3.907745563
A_21_P0012528		ENST00000601814	5.166594768	5.224080311	4.758810694	4.811759073
A_33_P3383955	DDB2	NM_001300734	5.163850218	5.10437434	4.862300103	4.8062974

A_33_P3225948	AJAP1	NM_001042478	5.158096756	5.072319225	6.186227421	6.0833524
A_23_P111054	HIST1H2BB	NM_021062	4.577953928	4.721062597	4.705944532	4.853054239
A_23_P151895	CILP	NM_003613	9.279650747	11.08987585	10.55887567	12.6186452
A_23_P61149	INPP5D	NM_001017915	6.853099673	6.612739903	7.477929877	7.215655347
A_23_P34345	VCAM1	NM_001078	4.546072134	4.216054306	4.490347425	4.16437488
A_24_P217834	HIST1H3D	NM_003530	4.310138019	4.792705576	4.073191973	4.529230803
A_21_P0004055	LOC102467226	NR_104999	9.113041656	9.299927695	10.77925801	11.00031404
A_23_P501754	CSF3	NM_000759	9.104871107	8.294037489	7.480998482	6.814778719
A_23_P436281	HIST2H4B	NM_001034077	4.301844701	4.189702198	2.955568392	2.87852125
A_24_P262127	RRAD	NM_004165	3.958442662	4.331647975	3.457673072	3.783665406
A_33_P3274397	CHM	NM_001145414	5.045598042	4.678601368	4.657485727	4.318718796
A_23_P395374	HIST1H4D	NM_003539	4.286673438	4.595301442	4.037065866	4.327722851
A_23_P316582	ZNF441	NM_152355	6.709256342	6.806136906	7.576387715	7.685789514
A_33_P3378880	HIST1H4C	ENST00000377803	4.484088708	5.659128755	3.651230379	4.60802276
A_32_P14721	DNAH12	NM_001291661	5.030169663	5.425542482	4.814017609	5.192400812
A_23_P96158	KRT17	NM_000422	5.024421366	6.127852243	4.618178958	5.632393508
A_33_P3697530	SEMA4D	NM_001142287	6.677145837	5.77833281	7.087000094	6.133016437
A_24_P39211	ING1	NM_198219	5.013718908	5.464869955	4.289070846	4.675015658
A_23_P102364	NGEF	NM_019850	4.462758236	4.69766663	3.418792987	3.59874967
A_24_P49183	EXD3	NM_017820	8.96045139	8.163848618	6.287092381	5.728156787
A_33_P3364038	IRGM	NM_001145805	6.647983351	7.181416775	4.618123267	4.988680949
A_23_P131208	NR4A2	NM_006186	4.982149418	4.889364193	5.049060019	4.955028682
A_23_P59069	HIST1H2BO	NM_003527	4.230053129	4.238857388	4.010159088	4.018505668
A_33_P3422802	ULBP1	NM_025218	4.426530302	4.677857796	4.202884578	4.441514017
A_23_P93258	HIST1H3B	NM_003537	4.228167117	4.354094806	4.032516743	4.152617369
A_21_P0010777	LINC00624	NR_038423	8.851480052	10.73473471	7.837209012	9.504665783
A_24_P3783	HIST1H2BM	NM_003521	4.217579143	4.097087608	4.461555159	4.334093501
A_32_P175301	DENND3	NM_014957	4.942653033	5.240276277	4.897560548	5.192468536
A_33_P3421053	SELE	NM_000450	6.550112401	10.78694977	5.89900689	9.71468688
A_33_P3400248	FGF20	NM_019851	6.534575778	5.953961278	7.601181245	6.925796003
A_32_P50223	PLK5	NM_001243079	8.718724829	7.343070481	7.159523269	6.029882237
A_21_P0009625	LOC100996324	NR_110763	6.479437311	6.788814209	5.82616204	6.10434668
A_23_P124108	ITGAM	NM_000632	6.451948155	5.89652833	6.418617223	5.866066711
A_24_P943472	NR1D2	NM_005126	4.871627484	4.822089968	5.002067528	4.951203622
A_32_P221799	HIST1H2AM	NM_003514	4.868118619	5.345244324	4.360997858	4.788420511

A_33_P3286273	EBLN1	NM_001199938	8.604532108	7.42781778	14.98420225	12.93503499
A_23_P8013	HIST1H2BL	NM_003519	4.145016231	4.207487832	4.220984427	4.284600983
A_23_P122443	HIST1H1C	NM_005319	3.827045261	3.827045261	4.427525343	4.427525343
A_21_P0013141		ENST00000448991	6.405843356	6.271103383	7.470191968	7.313064576
A_33_P3285639	TSGA10	NM_025244	8.536396773	8.245387964	8.260631534	7.97902366
A_22_P00020016		ENST00000538329	6.381673039	6.155433612	4.387836453	4.232281381
A_23_P111041	HIST1H2BI	NM_003525	4.115148737	4.24218906	4.263852685	4.395483704
A_32_P52153	UNC5B-AS1	NR_038453	4.798381186	4.568385401	5.046969679	4.805058561
A_33_P3404989	HIST1H3H	NM_003536	6.283442423	6.272711866	5.479128439	5.469771451
A_33_P7299171	AADAC	NM_001086	4.742677692	4.230590333	4.91084957	4.380603968
A_23_P420863	NOD2	NM_022162	8.31146198	8.032286024	7.404989032	7.156260843
A_33_P3293164	CHGA	NM_001275	6.244652911	7.66035545	3.655034985	4.483654666
A_21_P0011983		ENST00000432711	4.734960607	4.862927699	4.192974579	4.30629395
A_24_P256018		ENST00000415338	6.233838989	6.919275065	6.055738134	6.721591293
A_33_P3379391	H2AFJ	NM_177925	6.205314346	6.486407605	5.917292378	6.185338588
A_33_P3441031	LINC00944	BC038732	8.243014498	8.937767898	7.812866396	8.471365237
A_22_P00004211	LINC01511	NR_125810	6.192340915	6.286389993	9.594337579	9.740056076
A_23_P168828	KLF10	NM_005655	4.019427763	3.884418642	4.331762139	4.186261976
A_23_P94800	S100A4	NM_002961	4.689780541	4.827556025	3.979510311	4.09641961
A_22_P00011952		ENST00000521879	8.181290966	6.918056187	6.313741776	5.338866511
A_23_P39237	ZFP36	NM_003407	3.714299548	3.586849228	3.392275885	3.275875299
A_23_P70307	SMOC2	NM_022138	6.116845972	5.319957909	6.477262513	5.633420245
A_23_P23279	RCSD1	NM_052862	6.112126637	6.225625294	6.165573379	6.280064512
A_23_P366216	HIST1H2BH	NM_003524	3.989439886	4.091973182	3.991981891	4.09458052
A_22_P00015999	LOC101928106	ENST00000431401	8.029675622	7.800506781	7.036536746	6.835712324
A_23_P163278	PARP16	NM_017851	6.015074415	5.97239872	5.951111339	5.908889449
A_23_P80570	AADAC	NM_001086	4.590915149	4.121771675	4.272340014	3.835751585
A_21_P0014908		ENST00000586348	5.975974056	5.674010791	5.241302743	4.976462086
A_33_P6472133	AADACP1	NR_026915	5.963495824	5.623714633	3.84218216	3.623266734
A_21_P0006504	LOC102723911	XR_426531	5.95139243	7.298748582	9.741183801	11.94652382
A_23_P1492	AVPI1	NM_021732	4.531163447	4.854888576	4.353644029	4.664686434
A_32_P165933	IQCF2	NM_203424	7.812185174	7.852630812	4.009967394	4.030727998
A_33_P3364646	RASGEF1B	NM_152545	7.786362431	6.475255695	7.977933816	6.634569331
A_23_P429998	FOSB	NM_006732	3.600394824	3.529874132	3.322725269	3.257643272
A_21_P0014201		ENST00000447430	5.877278422	6.10033843	5.401672045	5.606681391

A_33_P3422233	UVSSA	NM_020894	4.027733517	3.861997538	3.710934312	3.558234207
A_23_P382775	BBC3	NM_014417	3.576168168	3.665565208	3.195611499	3.275495385
A_33_P3407469	MYH7B	NM_020884	7.67777243	7.336697784	5.944930469	5.680834981
A_23_P6596	HES1	NM_005524	4.01701272	4.053519808	4.349954743	4.389487648
A_23_P156788	STX11	NM_003764	5.819511961	5.256529485	6.216294686	5.614927253
A_33_P3376249	S100A2	NM_005978	4.005346188	4.112499469	3.539176415	3.633858459
A_21_P0011897	SEPT14	NM_207366	5.785339475	5.244015993	4.495742552	4.075084263
A_22_P00014868		ENST00000537961	5.779880039	6.549887354	5.473297271	6.202461009
A_23_P307502	CDKN2A-AS1	NR_024274	4.439034953	4.212846412	4.551601911	4.319677583
A_33_P3355252		ENST00000379526	4.423901082	4.549774383	4.538971887	4.668119298
A_23_P397293	LY6K	NM_017527	3.809383125	4.216610964	3.67761693	4.070758797
A_32_P54503	JHDM1D-AS1	NR_024451	5.742951524	5.921249097	5.077582944	5.235223263
A_24_P158946	FGD4	NM_139241	4.409789077	5.221994236	4.316326064	5.111316989
A_23_P67367	DHDH	NM_014475	4.402118962	4.198139718	3.641869677	3.473117803
A_23_P328074	SALL1	NM_002968	5.718803612	5.203843732	5.460212479	4.968537899
A_33_P3619171	PMAIP1	NM_021127	3.522863215	4.067684292	2.891045578	3.338154215
A_23_P46039	FCRLA	NM_032738	4.39491146	4.315016127	5.017374735	4.926163608
A_22_P00025235		ENST00000509666	7.48195264	7.488905805	6.476117963	6.482136381
A_33_P3404032	HIST3H2A	NM_033445	5.647422286	6.52571078	5.262370493	6.080775638
A_19_P00321383		ENST00000447430	5.64395786	5.169561236	5.093673989	4.665530866
A_33_P3270315		BC009492	4.344575214	5.533632662	3.468482575	4.417764113
A_24_P82419	H3F3C	NM_001013699	2.169369926	2.245240275	2.126892926	2.201277707
A_22_P00014914	SLCO5A1	ENST00000260126	4.341039189	4.258345107	4.543152262	4.456608052
A_32_P225355	CPEB2	NM_182485	4.324171199	3.83844086	3.888581945	3.451780963
A_33_P3229067	HIST1H2BN	NM_003520	4.317271764	4.204683252	3.694417624	3.598072292
A_21_P0010717	HIST2H2BA	NR_027337	7.296070083	7.827376992	6.533080824	7.008826115
A_22_P00019692	lnc-HIST4H4-1	lnc-HIST4H4-1:1	4.295547896	4.212322038	4.878865985	4.784338391
A_23_P69497	CLEC3B	NM_003278	4.291176854	4.688438888	3.8903366	4.250490256
A_21_P0004928	lnc-NT5DC1-4	lnc-NT5DC1-4:1	7.227450273	7.00476066	4.947321054	4.794885967
A_23_P54488	ACSBG1	NM_015162	7.213177796	7.826875496	5.254993792	5.702089052
A_33_P3367939	TMPRSS9	NM_182973	7.142696655	8.645541151	7.298643598	8.834299791
A_24_P68631	HIST2H2AB	NM_175065	3.412360866	3.641014006	2.721005988	2.903333293
A_23_P108501	EPHA4	NM_004438	4.234195809	4.535937722	3.229845044	3.46001381
A_23_P14184	THSD1	NM_018676	3.818407614	3.446268223	4.01348812	3.622336316
A_22_P00003942	lnc-CHAC1-4	lnc-CHAC1-4:1	7.090590059	9.889661282	4.619260081	6.442752605

A_23_P156708	TNXB	NM_032470	5.414914729	5.621427147	4.414681904	4.583047738
A_23_P161156	ZNF438	NM_182755	3.781578045	3.813261008	3.553249266	3.583019236
A_23_P74229	STK40	NM_032017	3.628463231	3.599717706	3.492049833	3.464385006
A_33_P3409746	C11orf91	NM_001166692	5.379207966	5.293487765	5.299576621	5.215125383
A_21_P0009009		ENST00000596416	5.344285834	5.670599638	4.978704629	5.282696612
A_22_P00014305	SMIM2-AS1	NR_104064	6.92838896	7.148357917	6.70899656	6.922000043
A_33_P3261298	SLC6A7	NM_014228	4.146334722	4.340020497	4.285893051	4.486097948
A_21_P0011898	SEPT14	NM_207366	4.144785401	4.398813701	3.575396544	3.794527769
A_23_P44674	CRIP1	NM_001311	3.74187091	3.818781271	3.490155077	3.561891675
A_23_P404494	IL7R	NM_002185	3.741075602	4.191579172	3.563629316	3.99276465
A_33_P3315959	LGSN	NM_016571	33.22806209	28.33766079	10.21328054	8.710122149
A_23_P312150	EDN2	NM_001956	5.312942456	5.940672374	6.625396175	7.408193924
A_23_P309381	HIST2H2AA4	NM_001040874	3.577111773	4.070593591	3.817649651	4.344314965
A_33_P3344127	HIST1H2AC	NM_003512	3.718125545	3.971843034	3.704861777	3.957674174
A_23_P392962	GRAP	NM_006613	6.836623493	9.433068864	5.554671168	7.664250592
A_22_P00009941	IKZF3	NM_001284514	6.832195915	7.717149056	8.277937353	9.350152897
A_33_P3252394	GADD45G	NM_006705	3.702957721	3.490278882	3.385953068	3.191481345
A_33_P8915099	LOC101928336	NR_110396	5.236011866	5.428695502	5.289305291	5.483950109
A_21_P0009159		ENST00000436469	5.230685181	5.201760404	5.408102309	5.378196445
A_23_P30813	HIST1H4K	NM_003541	3.544746856	3.834065177	3.418625271	3.697649687
A_23_P144476	SPRY1	NM_199327	6.764337168	5.540153304	6.339800034	5.192447277
A_23_P106194	FOS	NM_005252	3.289069364	3.10756351	2.87526261	2.716592501
A_33_P3323959	RELN	NM_005045	6.701985619	8.070689196	5.911312373	7.118541819
A_24_P12435	NCOA7	NM_181782	3.513885837	3.44262532	3.434808068	3.365151225
A_19_P00322523	LINC01021	ENST00000514844	6.67458525	7.393511985	9.537891625	10.56522816
A_33_P3304748			4.037912028	3.899679255	3.485144747	3.365835258
A_33_P3399571	VNN1	NM_004666	6.67244785	6.256769142	5.235587875	4.909422358
A_33_P3231252	NHLH2	NM_005599	5.168022467	5.106266634	5.379327263	5.315046421
A_23_P145024	ADRB2	NM_000024	4.03651266	3.897059519	3.10481399	2.997549106
A_23_P132057	LINC00158	NR_024027	6.666456598	7.075347637	3.805199947	4.038594126
A_19_P00318803	SEPT7-AS1	NR_120512	6.659610783	7.139585838	8.566708322	9.184132738
A_23_P214080	EGR1	NM_001964	2.084234616	2.055588883	1.976265051	1.949103252
A_33_P3288942	FAM107B	NM_001282695	3.630771065	4.50736123	3.725102424	4.624467351
A_23_P428184	HIST1H2AD	NM_021065	3.25583656	3.456272337	2.68881247	2.854341116
A_33_P3360216	HIST1H2AI	NM_003509	4.003493591	4.237381715	4.036857583	4.272694865

A_23_P164258	PIPOX	NM_016518	3.996206173	4.269353956	4.079655047	4.358506709
A_33_P3299066	NR4A2	NM_006186	5.0992598	5.448488075	4.881996614	5.216345387
A_21_P0011018	LOC101928620	XR_246466	5.09030937	5.011481639	3.833880186	3.77450932
A_21_P0011578		ENST00000577449	6.542276959	6.648426934	4.822942655	4.901196028
A_33_P3868357		ENST00000507936	3.60220032	3.717709428	3.569415039	3.683872846
A_33_P3423551	IER3	NM_003897	2.068224031	2.111862203	2.102338799	2.146696769
A_33_P3410194		ENST00000254810	3.594492412	4.180304847	3.902949699	4.539032963
A_22_P00022787	LOC101929181	NR_104624	3.961229273	3.793829669	3.755741576	3.597025781
A_33_P3384287	PALM	NM_002579	3.58983314	3.895159031	2.98783669	3.241961008
A_33_P3411744	EGOT	NR_004428	5.036929924	4.610242148	7.010175055	6.416329984
A_23_P431330	CRIPAK	NM_175918	3.950036832	4.452696129	3.867897784	4.360104531
A_22_P00008748	lnc-KIAA1967-2	lnc-KIAA1967-2:3	6.469618329	7.013132268	5.889903028	6.384714968
A_24_P350397	HSD3B1	NM_000862	29.55480743	32.43479854	15.48268446	16.99140665
A_23_P30567	CRHBP	NM_001882	5.022734126	4.616162422	4.381405829	4.026747272
A_23_P34915	ATF3	NM_001040619	2.060239181	2.138949341	1.910889187	1.983893523
A_33_P3358626	TAF4B	NM_001293725	3.937113358	3.906985295	4.020576074	3.989809327
A_22_P00024672	lnc-LINC00346-2	lnc-LINC00346-2:1	3.56714308	3.585525087	3.535305283	3.553523225
A_33_P3813128	SEMA3F	NM_004186	5.011164492	5.646912818	3.760555364	4.237643431
A_22_P00022660	STARD13-AS	NR_046693	5.006241894	5.12390648	5.010583343	5.128349968
A_23_P92161	ARL14	NM_025047	3.929232147	3.45210306	3.806073986	3.343900071
A_24_P329795	C10orf10	NM_007021	3.928260386	4.288523737	3.529064938	3.852717811
A_33_P3409124	NR1D2	NM_005126	4.991036451	4.837441413	5.509705437	5.340148788
A_33_P3532770	LOC283674	AK092120	6.402405906	6.167763344	9.458311495	9.111672673
A_32_P150030	PPM1D	NM_003620	3.541998633	3.089540834	3.380491921	2.948665121
A_23_P380318	EGR4	NM_001965	3.184882015	3.261364004	2.896905547	2.966472048
A_33_P3249897			4.957526353	4.639343866	5.396989392	5.050601418
A_33_P3406047	NLRP4	NM_134444	4.957401725	5.359060888	5.917345292	6.396781112
A_22_P00001177		ENST00000554926	6.343858119	6.038445144	9.090455914	8.652813216
A_23_P130149	ENO3	NM_001976	3.891018524	4.230090826	3.43163758	3.73067837
A_33_P3357530	SLC12A7	NM_006598	3.169640818	3.562834832	2.751394253	3.092704771
A_22_P00005132	LINC00944	NR_033878	3.877166358	3.990153536	4.748044005	4.886410029
A_19_P00316340	LOC100506860	NR_109780	3.516393691	3.558194644	3.426558992	3.467292039
A_32_P128701	USP53	NM_019050	3.86970416	3.061322468	4.044752359	3.199803076
A_24_P86389	HIST1H2AM	NM_003514	3.376109279	3.05267467	2.993227522	2.706473364
A_21_P0009110	ZFP90	ENST00000611381	6.278826942	7.921181987	6.623528781	8.356047612

A_33_P3273143		ENST00000406566	4.897896238	5.240174843	7.369836689	7.884861365
A_24_P9321	HIST1H3I	NM_003533	3.500802757	3.968754435	3.378917232	3.830576493
A_33_P3249534	NEFM	NM_005382	3.499407277	3.644596183	2.677982711	2.789091065
A_23_P104073	S100A3	NM_002960	3.853069067	4.064170122	4.012401568	4.232232095
A_23_P120243	HOXD1	NM_024501	6.254722905	5.703375913	6.754686911	6.159268639
A_33_P3256880	LOC642980	AK131413	6.246505142	6.451749635	4.766461664	4.923075641
A_21_P0005135	LOC102723649	NR_125867	4.871832826	5.635087299	5.371353311	6.212866062
A_23_P253321	PNOC	NM_006228	6.226659318	5.641747891	5.61194614	5.0847788
A_23_P163402	CYP1A1	NM_000499	3.83443705	3.690438969	5.661073843	5.448478419
A_21_P0014724	LOC100507336	XR_251947	3.83032159	4.036526844	4.063167034	4.281907515
A_23_P350574	FCRLB	NM_001002901	3.476023314	3.308599656	3.557861697	3.386496269
A_33_P3230269	GRHL1	NM_198182	4.8377678	4.19115469	6.419344976	5.56133922
A_23_P128215	SOCS2	NM_003877	3.469491595	3.395548262	3.405278913	3.332704109
A_33_P6474388	LOC101928053	NR_120540	3.817133886	3.916778844	3.417358596	3.506567558
A_21_P0000646	LINC00900	NR_034148	4.831048574	3.993746509	5.998005082	4.958449804
A_23_P57709	PCOLCE2	NM_013363	3.332135264	3.1756427	3.395016458	3.235570702
A_23_P59807	WNT2	NM_003391	6.151235638	6.340570923	6.357351448	6.553030987
A_23_P70670	CD83	NM_004233	3.109061083	3.331914152	3.134658798	3.359346675
A_23_P16523	GDF15	NM_004864	3.10784595	3.353366009	3.391438	3.659361851
A_33_P3382031	TMEM236	NM_001098844	6.112058052	5.528659705	6.414283282	5.802037417
A_22_P00007112	lnc-GLUD1-3	lnc-GLUD1-3:2	4.791308106	5.172012129	4.081815705	4.406145434
A_23_P41470	DDX60	NM_017631	3.439099184	3.036779404	3.192733168	2.819234284
A_23_P345118	PIM1	NM_002648	3.312189105	3.608787441	3.21563333	3.503585335
A_23_P119143	ICAM5	NM_003259	3.781557746	3.975741285	3.876772106	4.075844917
A_33_P3392525	ARL4D	NM_001661	3.437144962	3.341610526	3.951171777	3.841350116
A_22_P00021181	LSAMP-AS1	NR_109998	4.764708881	4.549919965	4.352571756	4.156361622
A_23_P42198	HIST1H3G	NM_003534	3.295011598	3.462899837	3.105231944	3.263450484
A_21_P0014894			4.749636317	5.192977294	4.931477052	5.391791424
A_19_P00323692	XIST	NR_001564	3.418706549	3.25784611	3.03371748	2.890971936
A_21_P0007481	LINC00944	NR_033878	3.748139904	3.730365708	3.819571454	3.801458521
A_33_P3220470	SMAD6	NM_005585	3.744765682	3.614073723	3.561012178	3.436733199
A_22_P00003637	lnc-CD59-1	lnc-CD59-1:1	4.727735639	4.608307748	5.24514797	5.112649664
A_23_P383422	NFKBID	NM_139239	3.737125032	4.206874796	3.72400337	4.192103765
A_24_P41801	LPA	NM_005577	5.999835089	6.791580568	3.989232125	4.515656011
A_33_P3367361	FAM107B	NM_001282695	3.398694842	3.269838033	3.404596528	3.275515964

A_23_P69310	CCRL2	NM_003965	5.990083309	6.464569028	4.492366729	4.848215513
A_24_P166407	HIST1H4B	NM_003544	3.064257439	3.222203788	2.845262466	2.991920777
A_24_P291231	PER3	NM_016831	5.975858555	6.071135861	4.576773599	4.649744312
A_24_P186608	DPY19L2P2	NR_003561	5.954354963	5.397480429	7.271381295	6.591333314
A_23_P72989	CCR4	NM_005508	5.951811693	5.514224894	9.153231371	8.480271032
A_32_P141238	ANO2	NM_001278597	5.949003159	5.863744251	4.776899208	4.708438459
A_23_P363878	RFTN2	NM_144629	4.681319485	4.529684356	5.035546223	4.872437147
A_24_P169048	RFPL3S	NR_001450	5.948383715	6.878592351	4.834953084	5.591043364
A_23_P312920	POU2AF1	NM_006235	5.945957678	5.640741502	6.844910023	6.493549086
A_33_P3422010	DNAH12	NM_001291661	5.944872077	7.27431153	4.648625962	5.688188577
A_23_P428129	CDKN1C	NM_000076	3.705413085	3.247774046	3.787615194	3.319823739
A_33_P3213468	ANKRD33B	NM_001164440	5.91277131	5.126954714	5.492478458	4.762519442
A_23_P23611	AMY1C	NM_001008219	4.653708998	4.361915169	5.009313443	4.695222736
A_32_P95960	CLEC2D	NM_001004419	5.897565475	6.314551845	4.988780967	5.341511883
A_33_P3316223	SNAPC1	NM_003082	3.354884202	3.441930675	3.436496672	3.525660678
A_24_P230948	PER3	NM_016831	5.860350888	5.475863653	13.95551411	13.03991756
A_33_P3393766	C17orf96	NM_001130677	3.672572662	3.620469202	3.689602877	3.637257806
A_21_P0003932	LOC101927115	NR_109876	4.620707603	4.110466459	4.142185134	3.684784782
A_24_P303454	TIAM2	NM_012454	3.345352985	3.446146104	3.201479782	3.297938103
A_23_P6293	UBASH3A	NM_018961	24.77815575	24.90305822	27.972066	28.11306842
A_23_P58266	S100P	NM_005980	4.617206854	4.919289505	3.931079904	4.188272418
A_23_P353667	MIR7-3HG	NR_027148	4.607459433	4.394016939	5.363559459	5.115090314
A_24_P137522	USP53	NM_019050	3.660877742	3.134275011	3.826705931	3.276249473
A_22_P00015676		ENST00000563268	3.336661657	3.177521452	3.290423724	3.133488812
A_23_P112159	AGO2	NM_012154	3.33545037	3.29117921	2.824246922	2.786760925
A_23_P81158	ADH1C	NM_000669	4.59997561	4.520721823	6.157985214	6.051888206
A_23_P145238	HIST1H2BK	NM_080593	3.211659119	3.431113595	3.357480456	3.586898985
A_21_P0014736	LOC100506538	XR_426345	5.813746004	5.240469321	5.305986209	4.782778252
A_22_P00011396	lnc-PABPN1L-1	lnc-PABPN1L-1:1	5.812037082	7.783122206	4.829983373	6.468016345
A_33_P3228450	FXYD3	NM_001136007	24.40979214	22.68124293	24.38716967	22.66022243
A_23_P19619	HIVEP1	NM_002114	3.324595328	2.950123092	3.483100365	3.090774607
A_21_P0008607	LOC100507480	ENST00000560056	4.566601846	4.467752413	3.399080851	3.325503774
A_23_P94103	SCARA5	NM_173833	3.632731254	3.898679054	2.914423552	3.127784925
A_23_P17345	MAFB	NM_005461	3.311649757	3.131400833	2.995086405	2.832067626
A_22_P00009529	LOC101927501	NR_110387	5.767965435	8.319978608	10.07086677	14.52668138

A_24_P268676	BHLHE40	NM_003670	3.297863108	2.927897437	3.898167278	3.460857413
A_23_P52610	DDB2	NM_000107	3.178602026	3.230937622	3.388110784	3.443895936
A_24_P277673	HIST1H4G	NM_003547	5.716465544	7.013122004	4.155084824	5.097575869
A_23_P36825	GPRC5A	NM_003979	3.597122522	4.140546925	3.054883646	3.516390951
A_23_P329340		ENST00000261383	5.689589698	5.204613004	7.246136095	6.628480462
A_23_P70480	HIST1H4L	NM_003546	2.967121026	3.158199281	2.708149206	2.882550054
A_33_P3269636	SBSN	NM_001166034	3.160172807	3.278863907	2.862914906	2.970441468
A_21_P0006734		ENST00000419836	5.675058763	5.91560159	8.234187324	8.583201279
A_33_P3363355	ICAM4	NM_022377	3.2731084	3.800817097	3.717062681	4.316348149
A_33_P3251703	CRIP1	NM_001311	3.156745552	3.407963522	2.807644767	3.031080836
A_23_P363174	HIST1H2AL	NM_003511	4.486243962	4.769530295	4.183133512	4.44727977
A_33_P3324909	JUND	NM_001286968	3.581225355	3.662956286	3.385795586	3.463066408
A_24_P179183	ANKRD12	NM_015208	3.579409996	3.32071159	3.709143252	3.441068499
A_33_P3316278	ABCC6P1	NR_003569	5.635446279	5.490731223	15.55163522	15.15227807
A_23_P390068	MISP	NM_173481	4.455461851	4.214670804	5.012371719	4.741483027
A_23_P354288	FAM71A	NM_153606	5.601942225	6.273443858	7.439075171	8.330792888
A_23_P32707	ESPL1	NM_012291	3.244326421	3.317438715	2.622864792	2.68197218
A_23_P160689	LRIF1	NM_018372	3.549005421	3.087133011	3.508649788	3.052029315
A_23_P145074	PNRC1	NM_006813	3.1242568	3.386369977	3.120523764	3.382323754
A_22_P00001271	lnc-ANKRD54-1	lnc-ANKRD54-1:1	5.575338887	7.296323927	5.762532081	7.541299562
A_21_P0014288	ANKRD10-IT1	AK124233	4.421659716	3.836464569	4.023231767	3.490767522
A_24_P174613	FBXW7	NM_033632	3.231166047	2.985874015	3.290660762	3.040852225
A_21_P0014801	KLHDC4	ENST00000567298	3.531342623	3.107106719	3.357621791	2.954255743
A_23_P122439	BTN2A2	NM_181531	3.52960862	3.528078365	3.362221164	3.360763479
A_33_P3569068	MAD1L1	NM_003550	3.225424583	3.653786349	3.251193139	3.682977172
A_22_P00008339	LOC101928554	XR_242926	5.509479219	6.076309235	4.936638102	5.444532685
A_23_P133438	FAM105A	NM_019018	5.495235394	5.906757989	23.42888268	25.18340526
A_23_P32233	KLF4	NM_004235	3.501885443	3.132548026	3.208403666	2.870019232
A_33_P3294524	ANKRD12	NM_001204056	3.501430406	3.575413563	3.995135749	4.079550609
A_23_P350551	C12orf57	NM_138425	2.90322953	3.180256548	3.088409443	3.383106383
A_23_P376557	MMP25	NM_022468	4.361843379	4.762994933	3.778935181	4.126477629
A_21_P0013116	LPAL2	NR_028093	5.480064129	6.415037719	8.245443396	9.652228358
A_23_P320261	DMKN	NM_001035516	4.351098403	4.466724896	4.114614335	4.223956479
A_33_P3289123	DCLK3	NM_033403	5.454219982	5.121207653	4.821391178	4.527016784
A_23_P149975	FAM107B	NM_031453	3.186880002	2.729486242	3.19190256	2.733787943

A_23_P132644	NCEH1	NM_020792	3.0741043	3.053483745	3.173145204	3.1518603
A_33_P3404588	FGD4	NM_139241	4.335344043	3.968178116	4.063201715	3.719083876
A_33_P3264895	RHEBL1	NM_001303126	3.465746125	3.411438011	3.397727443	3.344485179
A_33_P3247332		THC2686537	21.5181305	21.22046101	19.81567921	19.54156045
A_33_P7574262	CFLAR-AS1	NR_040030	3.462098749	3.3906101	3.627253451	3.552354533
A_21_P0013364	ZNF890P	NR_034163	21.49526475	27.18187412	21.87686611	27.66442877
A_23_P87560	BTG1	NM_001731	3.059048272	2.652177354	3.2071635	2.78059241
A_23_P326204	SGMS2	NM_152621	3.4590003	3.201363592	3.020039069	2.795097509
A_21_P0009360	LINC00673	NR_036488	4.284954399	3.977488187	4.032341213	3.74300122
A_33_P3212394	FRG2C	NM_001124759	21.23755864	15.56580962	13.52567482	9.913478425
A_22_P00016125	lnc-TIGD2-1	lnc-TIGD2-1:1	21.17187501	18.88335566	21.47402836	19.15284853
A_21_P0000879	LOC339166	NR_040000	5.35334917	5.357102521	5.684283495	5.688268872
A_32_P135348	TANC1	NM_033394	3.144071141	3.187774487	3.319963054	3.36611134
A_19_P00319646	LINC00673	NR_036488	3.033186699	2.699809735	2.947658031	2.623681507
A_23_P4773	LILRB5	NM_006840	5.327204705	6.121485162	8.977731187	10.31630119
A_23_P207319	MAP3K14	NM_003954	3.134569641	3.29866249	2.803212407	2.949958903
A_19_P00316705	lnc-MMRN1-2	lnc-MMRN1-2:6	20.89097096	18.93278363	21.24837036	19.25668267
A_22_P00009283		ENST00000449713	5.312063566	5.464442974	4.003901842	4.118755925
A_33_P3322730			5.311741924	4.621907849	4.842893661	4.213948748
A_23_P304450	GATA6	NM_005257	3.023407257	2.854814226	3.781580038	3.570709326
A_32_P100439	MTURN	NM_152793	3.403685198	3.159975635	3.527309152	3.274747907
A_24_P202567	ITPKC	NM_025194	3.007127708	2.984292451	2.667797385	2.647538905
A_19_P00318559	LINC01021	ENST00000512067	4.212733378	4.001194868	4.104136058	3.898050661
A_23_P130974	KIAA1683	NM_025249	3.387650304	3.247150928	3.013018808	2.888056896
A_24_P309095	RELN	NM_005045	5.226460514	6.077203517	4.379440363	5.092308706
A_33_P3242543	MAOA	NM_001270458	4.1842892	4.011077581	5.028091675	4.819950253
A_22_P00021182	lnc-LRRC40-2	lnc-LRRC40-2:2	4.182614533	4.337369883	4.209399486	4.36514587
A_33_P3252286	CRLF1	NM_004750	2.982983351	2.88582771	2.627230154	2.54166138
A_33_P3229678			5.198257051	4.95941205	4.259984444	4.064250378
A_33_P3381338	TNXB	NM_019105	4.166223114	4.1248229	4.037389225	3.997269248
A_33_P3354374	C1QTNF1-AS1	NR_040018	4.163247376	4.312555712	4.054340725	4.199743295
A_23_P103486	CYP2J2	NM_000775	5.183719408	4.784528702	5.407768156	4.991323781
A_33_P3728698		ENST00000517910	3.357533899	3.241583058	3.661380113	3.534936086
A_33_P3322499	ZNF833P	NR_028594	5.181831369	5.372529625	4.745899051	4.920554421
A_21_P0000502	RNU11	NR_004407	3.346812687	3.323553421	3.271365722	3.248630788

A_22_P00012285	TCERG1L-AS1	NR_120623	3.346708844	3.416046904	3.520632167	3.593573619
A_33_P3220837	MAFB	NM_005461	2.965118097	3.044632478	2.577047177	2.646154816
A_23_P51936	TNFRSF9	NM_001561	3.330050297	4.015618891	3.278826776	3.953849811
A_23_P121082	GBE1	NM_000158	2.945538084	2.777461591	2.859303403	2.696147581
A_22_P00013725	LOC643201	NR_036494	4.095592591	4.018369962	3.413323688	3.348965278
A_23_P500741	CBFA2T3	NM_005187	4.09304223	4.762091221	3.984086065	4.635325074
A_33_P3293798	KRTAP10-1	NM_198691	3.310525811	4.03387708	2.762838896	3.366520346
A_23_P12199	FAM46B	NM_052943	4.085685844	4.753274904	3.78853588	4.407571509
A_21_P0005987	LOC440896	NR_015361	3.036006495	3.435057925	3.138739357	3.551293952
A_22_P00005863	ERN1	ENST00000433197	2.934806975	2.812839294	2.870348645	2.751059789
A_22_P00022417	lnc-SFI1-1	lnc-SFI1-1:2	4.078462985	4.165692552	3.87823779	3.961184969
A_24_P29686	ZMIZ2	NM_031449	3.032814956	3.188233678	2.960193142	3.111890308
A_23_P82651	NPTX2	NM_002523	4.072646453	3.923253431	3.493969536	3.36580357
A_33_P3741059		ENST00000456414	5.036887975	5.523541136	4.133747182	4.533140844
A_32_P133072	SPON1	NM_006108	5.035733144	5.448752123	4.31474302	4.668628086
A_24_P287473	SAV1	NM_021818	3.020024603	2.903258555	3.428439767	3.29588278
A_33_P3269728	ANKRD10	NM_001286721	4.050918844	3.922278081	3.755130685	3.635882955
A_19_P00803850	LINC01539	NR_040025	4.049482026	3.955152767	3.438846651	3.358741626
A_22_P00007581	HDAC9	ENST00000406451	3.274570162	3.308823508	3.168327481	3.201469485
A_19_P00322407	STXBP5-AS1	NR_034115	3.273788138	3.134321061	3.08312476	2.951780159
A_33_P3212630	FAM90A1	NM_018088	4.997277995	5.09120008	5.898789681	6.009655362
A_23_P356616	ABTB2	NM_145804	3.00142812	3.155081242	2.944076094	3.094793174
A_23_P57784	CLDN1	NM_021101	4.017149916	4.386060671	4.537229322	4.953901025
A_22_P00017841	MATN1-AS1	NR_034182	4.979952875	4.408087388	5.741677936	5.082340884
A_32_P98072	TCHH	NM_007113	4.973216261	4.102761037	15.57905822	12.85227702
A_22_P00006508	LOC102723716	XR_426296	4.002703725	3.858856801	3.556098812	3.428301725
A_21_P0014188	LOC401317	BC087859	3.251166201	3.382453382	3.176833775	3.305119297
A_24_P170763	KHNYN	NM_015299	2.988700064	3.104743484	2.965501973	3.080644671
A_24_P241815	JUNB	NM_002229	2.986458084	3.011723285	2.845267631	2.869338372
A_19_P00318954		ENST00000580948	3.993147583	4.320270544	3.290306404	3.559851857
A_33_P3228460	FXYD3	NM_001136007	4.943358579	4.610968163	4.978938685	4.644155871
A_21_P0012930	LOC643201	NR_036494	4.9344948	5.206054345	4.375941192	4.616761914
A_33_P3301709	GNG4	NM_001098722	4.927704753	5.30448236	4.397636519	4.733884538
A_21_P0011750		ENST00000455211	4.926577622	4.34787846	4.853324211	4.283229742
A_33_P3243449	CD70	NM_001252	3.23486638	3.065983733	3.572477331	3.385969031

A_21_P0014248	VIM-AS1	NR_108061	2.971801	2.878225174	2.692284081	2.60750966
A_33_P3381483	ZNF331	NM_001253798	3.225897939	3.123156546	3.704962746	3.586963652
A_23_P398566	NR4A3	NM_173200	2.965579174	2.779779283	3.139492133	2.942796223
A_23_P430201	CEP128	NM_152446	3.954377755	3.371095221	3.954894486	3.371535733
A_23_P93282	HIST1H3J	NM_003535	2.960043511	3.249330437	2.832982522	3.109851697
A_33_P3280905		ENST00000461229	4.881577023	5.125660881	4.292986935	4.507640684
A_33_P3253214		ENST00000607355	2.695394497	2.660917879	2.908384518	2.871183559
A_21_P0013854		ENST00000440604	3.928479745	3.40990085	4.053833477	3.518707266
A_33_P3396891	AVPI1	NM_021732	3.198947067	3.570348356	3.276376163	3.656767055
A_33_P3695899	FLJ31104	NR_102755	3.194634173	3.154496683	3.294378779	3.252988094
A_24_P85200	ELMSAN1	NM_194278	2.938641061	2.85054515	3.035832523	2.944822962
A_33_P3290089		ENST00000595770	4.835320061	5.051241497	4.856507522	5.073375085
A_24_P782308	NEDD4L	NM_001144967	3.9110739	3.848303461	3.390773164	3.336353247
A_33_P3270429	NR1D2	NR_110524	4.81871873	5.405720985	6.466406098	7.254124821
A_22_P00002982		ENST00000562471	3.897962774	4.678411416	3.533587993	4.241081653
A_23_P202269	ANK3	NM_020987	3.174911439	2.850929097	3.71677858	3.337501662
A_33_P3279831	PNRC1	NM_006813	3.172886233	3.033696235	3.509701051	3.355735466
A_23_P144337	CCRN4L	NM_012118	2.921322109	2.812759839	2.745650645	2.643616684
A_33_P3270514	HAND2-AS1	NR_003679	4.793129707	4.466639466	5.865920793	5.466355998
A_22_P00021268	lnc-UBE2Z-1	lnc-UBE2Z-1:1	3.87043437	3.977550761	3.410330946	3.504713722
A_22_P00017797		ENST00000569134	3.862576812	4.114724606	3.820584013	4.06999053
A_33_P3286953	ADAMTS6	NM_197941	2.902902292	3.12014021	2.857487855	3.071327195
A_22_P00001368	lnc-AP002478.1-2	lnc-AP002478.1-2:2	4.745042582	4.47241223	4.582671721	4.319370521
A_21_P0005617		ENST00000517910	3.147935658	3.38640295	3.328779453	3.580946304
A_23_P18490	MAEA	NM_001017405	3.142565901	3.223932511	3.072911043	3.152474165
A_33_P3251776	HSD3B7	NM_001142777	2.892676136	2.875435444	2.709480347	2.693331523
A_33_P3299285	FCRLB	NM_001002901	3.83525206	3.355835725	3.866561472	3.38323138
A_33_P3305655	NBEAP1	NR_027992	4.715175803	4.248896979	5.759794001	5.190213973
A_22_P00019571	SNAPC1	NM_003082	2.886233286	3.344362771	3.130332265	3.627207384
A_23_P301247	HIST2H2AC	NM_003517	2.885118152	3.283257611	2.761272328	3.142321357
A_24_P22976	ARRDC2	NM_001025604	3.126697339	3.490856158	2.994315409	3.343056027
A_23_P47322	BTG4	NM_017589	4.700863337	4.354778096	4.594028462	4.255808579
A_23_P166248	RCAN1	NM_004414	2.791747316	2.703029665	2.778008172	2.68972713
A_23_P44569	ABCC2	NM_000392	3.820529345	3.381720239	3.811381943	3.373623467
A_23_P46470	ERRFI1	NM_018948	2.790236196	2.677151645	2.682233804	2.573526445

A_24_P151582	TEF	NM_003216	4.692471659	5.750982284	3.704332244	4.539941988
A_23_P16817	CLK1	NM_004071	2.871886937	2.691717371	3.264867426	3.060043991
A_23_P28582		ENST00000442128	3.80273545	4.259911762	4.53127925	5.076043292
A_24_P13001	SORCS3	NM_014978	16.57868421	16.80640643	17.36661213	17.60515721
A_33_P3348494	PCID2	NM_001258213	2.779204077	2.61745255	2.752609819	2.592406096
A_23_P125435	GABRB1	NM_000812	4.663659193	4.988372651	6.342288169	6.783878397
A_33_P3302957	PLEKHG4	NM_001129728	3.105095156	3.052925842	3.279935238	3.224828401
A_23_P212792	CEP135	NM_025009	2.857338105	2.690973147	2.719720344	2.561368009
A_23_P372860	HIST1H2AC	NM_003512	3.773922303	3.766005008	3.6944223	3.686671788
A_32_P79190	ANKRD20A12P	NR_046228	3.773701077	3.676110119	4.142825908	4.035689084
A_22_P00013430	BTG1	NM_001731	2.610271495	2.855916197	2.461757909	2.69342645
A_33_P3265359	HES6	NM_018645	3.079422261	3.074532297	3.211552347	3.206452568
A_22_P00011458		ENST00000416507	4.600350549	4.926450982	4.120401956	4.412480755
A_33_P3335915	SYNE1	AL713682	3.739622867	3.810587107	3.38929031	3.453606531
A_21_P0010748	SEPT14	NM_207366	3.738439932	4.033811243	3.440034373	3.711828887
A_23_P345460	PLEKHG4	NM_001129728	3.062531639	2.981687778	2.900445397	2.823880244
A_24_P157926	TNFAIP3	NM_006290	2.594428895	2.77871367	2.318396751	2.483074698
A_23_P58983	TBC1D22B	NM_017772	3.059201882	2.80447731	3.486992575	3.196647993
A_22_P00022564		ENST00000520603	3.724574352	3.78815589	3.53590839	3.596269246
A_22_P00017934	lnc-ZFP90-2	lnc-ZFP90-2:1	15.90482118	22.72491794	13.85702941	19.79901897
A_19_P00810806	SMIM2-AS1	NR_104064	3.722490812	3.54322984	4.256824032	4.051831608
A_23_P1602	CDC42EP2	NM_006779	2.734414074	2.805283625	2.995660315	3.073300751
A_23_P156667	PPP1R10	NM_002714	2.818927033	2.864808835	2.712286408	2.756432493
A_22_P00006097		ENST00000565297	2.818201793	2.885440807	2.5582731	2.619310518
A_21_P0008064		ENST00000461527	15.78948259	18.2407732	5.139265472	5.937127789
A_23_P94472	FBP2	NM_003837	3.708875017	5.149113634	4.023278479	5.58560695
A_33_P3316539	SLC7A2	NM_001008539	3.047102685	3.192385366	2.928504484	3.068132526
A_23_P422933	ARHGAP20	NM_020809	4.532604687	4.302213922	7.014769827	6.658211447
A_22_P00001217	lnc-ANKRD11-1	lnc-ANKRD11-1:2	3.702654588	3.759439188	3.787742541	3.845832066
A_21_P0002605	LOC102724744	XR_425445	15.71872046	18.38704534	15.67711822	18.33838094
A_22_P00021240	lnc-PSMG4-1	lnc-PSMG4-1:1	3.689896733	4.524117009	4.6256305	5.671403602
A_23_P147805	UPP1	NM_001287428	2.803002381	2.975475171	2.733993751	2.90222034
A_23_P124742	CHKA	NM_001277	2.801503926	2.730881672	2.937917304	2.863856247
A_33_P3293918	SH2D3C	NM_170600	4.484011928	7.140181326	4.492399901	7.153538036
A_23_P41765	IRF1	NM_002198	2.790106809	3.129884699	2.75523004	3.090760655

A_24_P250922	PTGS2	NM_000963	2.702415969	2.757718512	2.682068646	2.736954799
A_33_P3227899	LOC440896	NR_015361	2.781704903	3.500546688	2.713033596	3.414129501
A_32_P186731	ISM1	NM_080826	3.65099456	3.579257433	3.428787285	3.361416231
A_23_P153251	ZNF274	NM_133502	3.002534869	2.919577442	2.891091799	2.811213447
A_22_P00020093		ENST00000429352	4.445150938	5.304980143	5.443779481	6.496774226
A_23_P81898	UBD	NM_006398	3.639556491	4.78092041	6.157993788	8.089138954
A_22_P00008182		ENST00000527770	15.1332133	15.5215018	16.38293436	16.80328825
A_24_P89891	TRAF1	NM_005658	2.770405035	2.781340015	2.616001931	2.626327471
A_24_P80204	MALL	NM_005434	2.768826119	2.803621002	2.541375301	2.573311887
A_22_P00006798	LOC101927115	NR_109876	4.427010226	4.408371491	5.001824934	4.980766096
A_23_P500861	SYNE1	NM_182961	2.682775487	2.617912865	2.638144882	2.574361313
A_23_P58770	HAND1	NM_004821	3.619043706	4.812435243	4.064960877	5.405395065
A_23_P408094	MXD1	NM_002357	2.98310744	3.180731511	3.109065929	3.315034463
A_23_P32253	NFIL3	NM_005384	2.675614792	2.636855494	2.857907944	2.816507923
A_33_P3352213		THC2700874	4.390738886	4.721661557	4.648315595	4.998651393
A_33_P3356816	LCE1B	NM_178349	14.77905035	15.29231123	15.1579351	15.68435425
A_23_P145761	ARL4A	NM_005738	2.746820172	2.817052492	2.923760891	2.998517336
A_23_P161624	FOSL1	NM_005438	2.662090842	2.898250566	2.581515934	2.810527687
A_33_P3222424	G-CSF (CSF3)	NM_000759	14.66015985	15.47215558	14.98282468	15.81269215
A_33_P3613000	ZBTB10	NM_001105539	3.573937985	4.220152537	4.404623773	5.201037139
A_33_P3247838	ZNF844	NM_001136501	2.951370686	3.10100974	3.32663867	3.49530439
A_33_P3318668	CYSRT1	NM_199001	3.56737831	3.481425561	3.627057513	3.539666848
A_33_P3261408	TMIE	NM_147196	4.314356152	4.103194461	5.975569902	5.683101826
A_33_P3514859	LOC101929709	NR_125822	2.935012786	3.149262192	2.78297807	2.986129279
A_33_P3211804	RUNX1	NM_001001890	2.638080625	2.712803112	2.695767185	2.772123619
A_23_P142574	MOGAT1	NM_058165	3.5447296	3.547760124	3.381069484	3.383960088
A_33_P3404052	TNFAIP8	NM_001286813	2.927736956	3.554366221	3.144878757	3.817983307
A_33_P3336282	TAF4B	NM_001293725	4.260340487	5.760960782	4.283389952	5.792128963
A_23_P52531	FAM24B	NM_152644	2.904396025	2.94584768	2.984367833	3.026960849
A_33_P3420224	ENTPD8	NM_001033113	13.93457475	15.54096861	4.113486155	4.587693588
A_23_P213832	SPINK7	NM_032566	2.890324625	2.971928701	2.993552701	3.07807127
A_23_P103110	MAFF	NM_012323	2.599107961	2.563404198	2.5339586	2.499149789
A_32_P226205	ZFHX2	NM_033400	2.881999767	3.2242758	3.215453386	3.597331498
A_33_P3329098	WTAP	NM_004906	2.670460461	2.751494011	2.530116453	2.606891347
A_23_P426153	CCDC37	NM_182628	4.191838868	4.879895622	11.00506965	12.81146363

A_23_P115375	HIST2H3D	NM_001123375	2.458204125	2.627332731	2.546933983	2.722167353
A_23_P303803	C19orf18	NM_152474	3.447802775	3.979597111	4.788142871	5.526673298
A_23_P50646	PINLYP	NM_001193621	2.858093498	3.081811348	2.926443132	3.155511064
A_21_P0004421	LUCAT1	NR_103548	3.428403006	3.38525901	3.606707763	3.56131993
A_23_P116653	BCL2L14	NM_138722	4.137859031	4.732491195	4.585803558	5.244807713
A_23_P19754	CPA4	NM_016352	3.421348744	3.486840119	3.302703249	3.365923515
A_33_P3387045	CYB5D1	NM_144607	2.640701411	2.698539681	2.748800116	2.80900603
A_23_P354027	KCTD11	NM_001002914	2.639856899	2.784721788	2.570279797	2.71132657
A_23_P72096	IL1A	NM_000575	2.638424361	2.658458551	3.252966608	3.277667165
A_24_P337058	CCNL1	NM_020307	2.631660694	2.974218284	2.950877497	3.33498685
A_33_P3397506			4.105391543	4.616095599	4.4508664	5.004546969
A_22_P00017055	LOC102723474	ENST00000439406	3.393873117	3.411090664	4.218451123	4.239851857
A_23_P56938	REL	NM_002908	2.827309956	3.266772115	3.426043841	3.95857004
A_23_P90172	PPP1R15A	NM_014330	2.417973595	2.671737161	2.438513602	2.694432819
A_33_P3391005	NEDD4L	NM_001144967	2.813781624	3.019314342	2.884942964	3.095673663
A_21_P0013256		ENST00000439784	3.37201056	3.592817217	3.612760325	3.849331806
A_21_P0014715		BC013229	3.370539235	3.500982532	3.929287234	4.081354647
A_33_P3240078			2.608292633	2.757994576	2.546333888	2.692479733
A_23_P14124	RASL11A	NM_206827	3.361740583	3.500717798	2.782985135	2.898036107
A_33_P3375299		ENST00000404289	4.043502664	4.220176635	5.936723125	6.196118143
A_22_P00008458		ENST00000522715	3.324516098	3.407611651	3.653829921	3.745156601
A_33_P3379482	LINC00910	ENST00000586231	3.995479876	6.116451359	4.749381893	7.270556787
A_33_P3260455			0.299435905	0.284084349	0.303260052	0.287712438
A_21_P0000235	SNORA6	NR_002325	0.2993962	0.248003736	0.281186297	0.23291963
A_24_P205994	EPGN	NM_001270989	0.086632136	0.083559243	0.073296541	0.07069667
A_23_P131935	FERMT1	NM_017671	0.356046308	0.31102971	0.351800757	0.307320945
A_21_P0014312	SNHG19	ENST00000563192	0.382873596	0.363518921	0.388557644	0.368915634
A_23_P18372	B3GNT5	NM_032047	0.235980975	0.188605858	0.23856116	0.19066805
A_23_P65442	IRF9	NM_006084	0.350884474	0.373352203	0.344313794	0.366360791
A_19_P00808348	LOC101927151	ENST00000586784	0.288270457	0.273049884	0.260692443	0.246927979
A_23_P111000	PSMB9	NM_002800	0.378336659	0.379340484	0.363685951	0.364650903
A_23_P114839	FHL3	NM_004468	0.286334447	0.240423928	0.287251259	0.241193739
A_23_P35456	SH3PXD2A	NM_014631	0.347136596	0.325822991	0.341502115	0.320534457
A_33_P3289128	ZBTB42	NM_001137601	0.228448899	0.225450749	0.211811237	0.209031439
A_23_P72568	SNX4	NM_003794	0.281250738	0.222494419	0.287036274	0.227071294

A_33_P3685216	A1BG	NM_130786	0.280745347	0.279240463	0.301202386	0.299587845
A_23_P24444	DHCR7	NM_001360	0.370230925	0.387800712	0.353928144	0.370724262
A_21_P0000403	SNORD114-6	NR_003198	0.278210222	0.200713186	0.294967933	0.212802941
A_23_P208293	PVRL2	NM_001042724	0.368131356	0.338368922	0.365026976	0.335515522
A_32_P34444	FHOD3	NM_025135	0.337596138	0.284906366	0.339202545	0.286262055
A_23_P113613	CDCP1	NM_022842	0.335410046	0.327582697	0.286043661	0.279368359
A_23_P96965	SYNC	NM_030786	0.363879052	0.359777828	0.385710782	0.381363496
A_24_P317762	LY6E	NM_002346	0.358489904	0.378732875	0.37276532	0.393814386
A_24_P197964	TRIM14	NM_014788	0.215721426	0.043140975	0.174093173	0.034815963
A_21_P0003325		ENST00000514265	0.21358064	0.161938445	0.252265081	0.191269278
A_23_P7282	ELMOD2	NM_153702	0.326063906	0.306311166	0.354321029	0.33285649
A_33_P3358740	OSBPL7	NM_145798	0.32566134	0.302282094	0.305004849	0.283108533
A_21_P0011280	FLJ22447	ENST00000229465	0.264344319	0.234801089	0.251902342	0.223749632
A_23_P134113	SLC18B1	NM_052831	0.263938988	0.211304494	0.262287938	0.209982695
A_33_P3263232	LRRC3	ENST00000291592	0.067755918	0.041695967	0.081632307	0.050235287
A_23_P370682	BATF2	NM_138456	0.262392479	0.264042599	0.186466545	0.187639185
A_24_P721699	A1BG-AS1	AK027222	0.209138592	0.177624712	0.247413619	0.210132297
A_33_P3248439	MVB12B	NM_033446	0.319336824	0.294978254	0.300178256	0.277281075
A_21_P0000408	SNORD114-11	NR_003204	0.258442848	0.275434742	0.254598641	0.271337789
A_33_P3235611	SLC46A1	NM_080669	0.205527993	0.217933796	0.220613238	0.233929595
A_32_P86739	CASC10	NM_001010911	0.20463518	0.190845769	0.227093904	0.211791104
A_33_P3645465	PDCD4-AS1	NR_026932	0.255493795	0.218652992	0.235563104	0.201596196
A_23_P69810	AGPAT9	NM_032717	0.254890879	0.236595847	0.236580729	0.219599926
A_23_P209731	ARMC9	NM_025139	0.315153979	0.287022426	0.351754083	0.320355499
A_22_P00001617	ZBED1	NM_001171135	0.254161721	0.227826506	0.297787145	0.266931639
A_32_P142818	DLX1	NM_178120	0.253856409	0.217074698	0.274539519	0.234760996
A_24_P406714	MLLT10	NM_004641	0.200438844	0.133937554	0.209707774	0.140131252
A_23_P48198	GLT8D2	NM_031302	0.311758159	0.303869451	0.326101762	0.317850104
A_33_P3278941	REC8	NM_001048205	0.251441617	0.247692938	0.225895313	0.222527497
A_23_P98844	ARHGEF25	NM_182947	0.198942091	0.172901498	0.229468607	0.199432236
A_24_P322474	PDE4A	NM_006202	0.310424639	0.309342982	0.345298978	0.344095804
A_23_P168909	ZFPM2	NM_012082	0.060360452	0.060870191	0.071023231	0.071623016
A_19_P00320067	LOC100130417	NR_026874	0.248945879	0.226746602	0.246193708	0.22423985
A_24_P406754	LOXL4	NM_032211	0.248843897	0.248893957	0.285263424	0.285320811
A_24_P44462	TPM1	NM_000366	0.334530478	0.305496097	0.370066177	0.337947602

A_23_P85209	IL13RA2	NM_000640	0.246143923	0.258882609	0.230598393	0.242532551
A_23_P143713	APOBEC3G	NM_021822	0.245960946	0.27586665	0.225245799	0.252632806
A_23_P69109	PLSCR1	NM_021105	0.305661702	0.275930158	0.286780499	0.258885519
A_33_P3253234	IQSEC2	NM_001243197	0.2455404	0.263271413	0.245800789	0.263550606
A_33_P3335966	TPM1	NM_001018005	0.343635573	0.33878777	0.368637421	0.363436907
A_21_P0014256	STK32C	ENST00000456004	0.242984221	0.253760498	0.263430864	0.275113943
A_21_P0000287	SNORD47	NR_002746	0.241962781	0.225121916	0.265876598	0.247371306
A_24_P6083	CHCHD10	NM_213720	0.300830586	0.27728747	0.331244787	0.305321443
A_23_P62642	CFAP45	NM_012337	0.240281781	0.257395538	0.196548593	0.21054751
A_23_P38482	PHF23	NM_024297	0.299564114	0.294087317	0.297357491	0.291921036
A_23_P371765	SPATC1L	NM_032261	0.055828425	0.055657881	0.053488793	0.053325396
A_23_P154488	PNPT1	NM_033109	0.299118	0.269704929	0.271613975	0.244905448
A_24_P303091	CXCL10	NM_001565	0.238844143	0.192319434	0.232130653	0.186913672
A_33_P3311653	PDS5A	ENST00000507766	0.187312167	0.17383894	0.200348985	0.185938029
A_33_P3511265	POSTN	NM_006475	0.323830612	0.319849509	0.395491942	0.39062985
A_23_P398491	C15orf57	NM_001289132	0.234319812	0.216951131	0.25721528	0.238149499
A_23_P20743	TMEM246	NM_032342	0.233654323	0.19359458	0.224772217	0.1862353
A_23_P145874	SAMD9L	ENST00000411955	0.320227552	0.294959461	0.298877948	0.275294484
A_23_P417942	FNBP1L	NM_001024948	0.232480949	0.203909518	0.24071158	0.211128622
A_23_P35725	ANO3	NM_031418	0.182338136	0.081546106	0.165422433	0.073980987
A_23_P206022	ITGA11	NM_001004439	0.319343452	0.328861569	0.384518164	0.395978829
A_19_P00804072	RNF213	NM_001256071	0.182136016	0.200648447	0.207137051	0.228190604
A_23_P80839	MAP6D1	NM_024871	0.290378613	0.303815628	0.341286698	0.357079439
A_33_P3343175	CXCL10	NM_001565	0.231099849	0.245324029	0.212896555	0.226000324
A_33_P3402020	CCDC8	NM_032040	0.180101482	0.184174832	0.188844896	0.193115995
A_23_P337934	FBLIM1	NM_017556	0.288773932	0.29482094	0.30392253	0.310286754
A_23_P44264	EMX2	NM_004098	0.229211607	0.194878251	0.244801845	0.208133244
A_23_P397238	FKBP1A	NM_054014	0.2281555	0.247440577	0.236148709	0.256109421
A_33_P3308686	CC2D2A	NM_020785	0.227319179	0.206602685	0.27859429	0.253204893
A_33_P3253249	FTH1	NM_002032	0.508588635	0.489256655	0.514812546	0.495243989
A_23_P364792	TXLNGY	NR_045129	0.049938608	0.050259302	0.052792697	0.053131719
A_33_P3209229	RAB26	NM_014353	0.177645127	0.225389113	0.155247096	0.196971377
A_33_P3226810	TNFSF10	NM_003810	0.17699758	0.189759667	0.130776807	0.14020623
A_23_P206018	TPM1	NM_001018004	0.32495397	0.323885122	0.337187137	0.336078051
A_33_P3398448	PARP10	NM_032789	0.312182567	0.326778503	0.29664219	0.310511544

A_21_P0000191	PSG6	NM_002782	0.176055363	0.158521526	0.202377791	0.182222431
A_33_P3341499	WNT5A	NM_003392	0.308635671	0.256276902	0.320918111	0.266475677
A_32_P228167	C12orf50	NM_152589	0.278815392	0.263679039	0.326780238	0.309039966
A_33_P3372212	PSG10P	L14723	0.171277985	0.139966744	0.196445314	0.160533246
A_23_P349566	CCDC85A	NM_001080433	0.170914688	0.06874389	0.214693558	0.086352265
A_23_P137248	PRKY	NR_028062	0.170028545	0.157817016	0.178111545	0.165319492
A_32_P129968	ZNF284	ENST00000421176	0.21815524	0.253536291	0.255202566	0.296592059
A_23_P47148	NOX4	NM_016931	0.045872926	0.041431252	0.040486479	0.036566351
A_33_P3305472		AK123993	0.217354363	0.204541939	0.223802072	0.210609574
A_23_P121441	NLGN4Y	NM_014893	0.044938177	0.045047341	0.044651293	0.04475976
A_23_P115683	HPS6	NM_024747	0.301614615	0.304877868	0.33101553	0.33459688
A_21_P0000417	SNORD114-20	NR_003213	0.27293565	0.274646525	0.309061593	0.31099892
A_24_P68908	LOC344887	NR_033752	0.214311577	0.216243413	0.28827412	0.290872666
A_23_P432947	GREM1	NM_013372	0.271373795	0.23895348	0.328185032	0.288977628
A_33_P3270485	CBX6	ENST00000407418	0.298634921	0.27438674	0.295806955	0.271788395
A_24_P230540	RNF212	NM_194439	0.164192633	0.156690762	0.183776082	0.175379453
A_33_P3846177	B4GALNT1	NM_001478	0.211565637	0.198621852	0.228555625	0.214572377
A_23_P64879	KCNJ8	NM_004982	0.163566259	0.161416579	0.148769932	0.146814714
A_22_P00001349	LINC00667	NR_015389	0.043042697	0.04103266	0.056961687	0.054301652
A_32_P56249	USP30-AS1	NR_038996	0.162907263	0.134322669	0.140021585	0.115452637
A_33_P3254380	SLC9A7	NM_001257291	0.2106552	0.23083599	0.240428329	0.263461388
A_21_P0000402	SNORD114-5	NR_003197	0.210119532	0.221173969	0.213359385	0.224584272
A_23_P132159	USP18	NM_017414	0.266807262	0.277237722	0.239449155	0.248810088
A_33_P3381771	GID4	NM_024052	0.266374725	0.220512754	0.307428047	0.254497889
A_33_P3535523	LOC286068	BC015720	0.161177464	0.179240513	0.176222323	0.19597144
A_23_P34233	QPRT	NM_014298	0.161022376	0.08197121	0.160039921	0.081471074
A_23_P47691	TRIM21	NM_003141	0.20860762	0.186566003	0.206281076	0.184485283
A_33_P3258923	MKRN3	NM_005664	0.041823779	0.041798756	0.046757138	0.046729164
A_24_P66932	LOC729080	NR_033244	0.265945578	0.301568368	0.233679636	0.264980478
A_21_P0000607	ST3GAL4-AS1	NR_033839	0.158547044	0.234209346	0.161417161	0.238449149
A_23_P140748	NDRG4	NM_022910	0.205302326	0.181595631	0.224422045	0.198507556
A_33_P3340342	CMTM3	NM_144601	0.261678355	0.281751667	0.276147906	0.297331175
A_23_P97064	FBXO6	NM_018438	0.155696728	0.214570535	0.125616968	0.173116677
A_32_P76035	PNPT1	NM_033109	0.200950574	0.254206225	0.215682454	0.272842328
A_33_P3399064	RNA5-8S5	NR_003285	0.283160437	0.367902861	0.228160839	0.29644334

A_33_P3311267	KRTAP19-2	NM_181608	0.151718229	0.153519518	0.15968617	0.161582059
A_33_P3399755	EPB42	ENST00000570199	0.197959893	0.237071746	0.205491612	0.246091542
A_24_P85775	THEMIS2	NM_001039477	0.151349884	0.144214766	0.159421624	0.151905978
A_21_P0014131		THC2707919	0.151267991	0.169639187	0.195878431	0.219667476
A_21_P0000409	SNORD114-12	NR_003205	0.254058355	0.251596109	0.281640362	0.278910801
A_23_P137097	SLC16A2	NM_006517	0.253145284	0.251429621	0.269773716	0.267945356
A_21_P0000406	SNORD114-9	NR_003201	0.25176417	0.242672891	0.263435866	0.253923119
A_23_P1962	RARRES3	NM_004585	0.250870767	0.264239305	0.232084358	0.244451796
A_24_P498854	LINC01114	NR_038231	0.148342453	0.104815034	0.206545961	0.145940164
A_33_P3221408	NTNG1	NM_001113226	0.14764627	0.098243096	0.177125423	0.117858379
A_24_P237511	EIF1AY	NM_004681	0.035436166	0.035362851	0.036807816	0.036731663
A_33_P3422712			0.190740054	0.19416566	0.196124029	0.199646328
A_24_P278299	ASB13	NM_024701	0.190360226	0.198006803	0.188674432	0.196253292
A_33_P3373446	LOC647264	ENST00000453638	0.144604705	0.123305881	0.151393717	0.129094939
A_33_P3319870	GREM1	NM_001191323	0.271476844	0.263579407	0.327581662	0.318052099
A_24_P145009	ZNRF2P1	NR_003502	0.142922878	0.158388641	0.157709238	0.174775042
A_33_P3327041	PLCE1-AS1	NR_033969	0.03339859	0.030078932	0.076530612	0.068923841
A_33_P3250750		CU677518	0.186661361	0.170348493	0.167305979	0.152684632
A_22_P00006598	LOC101927675	XR_427815	0.186487263	0.195717923	0.198486875	0.208311487
A_23_P19987	IGF2BP3	NM_006547	0.186002434	0.185024431	0.246183269	0.244888835
A_24_P810290	PPAPDC1A	NM_001030059	0.241105615	0.249355955	0.263852179	0.272880879
A_23_P127911	PAMR1	NM_015430	0.238516852	0.29418213	0.248801615	0.306867159
A_33_P3271276	PSG5	NM_001130014	0.138295435	0.149735116	0.20279445	0.219569435
A_33_P3388061	KRTAP21-2	NM_181617	0.137517136	0.07622659	0.163962375	0.090885348
A_23_P164958	CCDC8	NM_032040	0.181825984	0.199702134	0.196001404	0.215271205
A_23_P75741	UBE2L6	NM_198183	0.236380068	0.220292559	0.233727367	0.217820395
A_23_P166779	LINC00312	NR_024065	0.235214444	0.226109557	0.244624738	0.235155589
A_23_P82775	SOX17	NM_022454	0.030526425	0.053678144	0.039050658	0.068667287
A_33_P3240328	PITX1	NM_002653	0.232165228	0.273551061	0.233521732	0.275149375
A_24_P227069	GPAM	NM_020918	0.176245016	0.17473332	0.226163701	0.224223841
A_21_P0000422	SNORD114-26	NR_003219	0.229366228	0.207833909	0.232185813	0.210388798
A_21_P0000412	SNORD114-15	NR_003208	0.228058078	0.196732613	0.245547358	0.21181961
A_33_P3261328	FLG	NM_002016	0.128958459	0.141352632	0.174011249	0.190735437
A_33_P3315779	HERC6	NM_001165136	0.1287441	0.149101993	0.108637105	0.125815544
A_23_P121253	TNFSF10	NM_003810	0.128707656	0.162574843	0.112231992	0.141763894

A_33_P3391316		ENST00000594169	0.171195106	0.155021275	0.243956701	0.220908645
A_21_P0008404	LOC102723354	NR_110546	0.127078744	0.146883298	0.125857813	0.145472091
A_23_P36882	NTS	NM_006183	0.12674529	0.150074947	0.116919901	0.138441025
A_33_P3332175		ENST00000451170	0.223079699	0.250087735	0.23675728	0.265421247
A_21_P0000420	SNORD114-23	NR_003216	0.222209542	0.221312586	0.238152501	0.237191191
A_23_P327140	RNF213	NM_001256071	0.125586146	0.173792459	0.117512473	0.162619703
A_23_P50775	LRFN3	NM_024509	0.221506075	0.192323039	0.207020481	0.179745897
A_23_P142750	EIF2AK2	NM_002759	0.246139226	0.264330703	0.247960524	0.266286608
A_22_P00002230	TMEM26-AS1	NR_120643	0.164481281	0.17442835	0.168873052	0.179085716
A_23_P51487	GBP3	NM_018284	0.217107504	0.230614212	0.224888406	0.23887918
A_23_P120002	SP110	NM_004510	0.216453218	0.227022284	0.203486134	0.213422038
A_33_P3272189	MFSD9	NM_032718	0.120513418	0.083145543	0.146793249	0.101276725
A_21_P0000425	SNORD114-28	NR_003221	0.215208324	0.231699593	0.232828823	0.250670339
A_33_P3262181	APOBEC3F	NM_001006666	0.212270201	0.203194475	0.198209483	0.189734931
A_21_P0000418	SNORD114-21	NR_003214	0.211825931	0.204504519	0.221532892	0.213875976
A_24_P941912	DTX3L	NM_138287	0.158809711	0.166427871	0.144171339	0.151087291
A_33_P3276997	MAP6	NM_207577	0.117031814	0.082073943	0.149304847	0.104706892
A_21_P0013996	TXLNGY	ENST00000593000	0.022770578	0.022873284	0.027294778	0.027417891
A_21_P0000419	SNORD114-22	NR_003215	0.206574517	0.198273555	0.214979067	0.206340378
A_23_P47728	MAP6	NM_033063	0.113690192	0.060239996	0.142760477	0.075643205
A_33_P3219591	RNF213	NM_020954	0.154537038	0.161961424	0.121114433	0.126933105
A_23_P6263	MX2	NM_002463	0.231181298	0.205253802	0.19908579	0.176757876
A_23_P70818	SMO	NM_005631	0.153672295	0.158947493	0.184705418	0.191045908
A_23_P250358	HERC6	NM_017912	0.11269401	0.160263907	0.097343194	0.138433272
A_21_P0001111		ENST00000440492	0.112264746	0.03841969	0.111310027	0.038092962
A_23_P159952	BEX1	NM_018476	0.230114437	0.187632421	0.249575026	0.20350034
A_24_P348861	TTTY15	NR_001545	0.022077924	0.0221166	0.023077048	0.023117474
A_23_P73848	TTTY14	NR_125733	0.021541853	0.022005066	0.029587866	0.030224093
A_24_P392110	PSG8	NM_182707	0.150980206	0.185162789	0.156664487	0.192134016
A_33_P3618429	PRKXP1	NR_073405	0.150631007	0.162653132	0.15550753	0.16791886
A_23_P108662	MOGS	NM_006302	0.225355849	0.217470609	0.250221442	0.241466151
A_21_P0000216	SNORD36C	NR_000016	0.148528749	0.166051015	0.157930337	0.176561729
A_33_P3333982	PDE4DIP	NM_022359	0.223416298	0.233660754	0.283190448	0.296175768
A_24_P942743	ZFY	NM_003411	0.020240204	0.020339867	0.02547067	0.025596088
A_23_P204087	OAS2	NM_016817	0.144737721	0.157948549	0.161683966	0.17644155

A_33_P3255404	CLDN11	NM_005602	0.194471111	0.183265097	0.223090014	0.210234892
A_33_P3216232	ITGB1BP1	NM_004763	0.194022735	0.187605503	0.194811356	0.18836804
A_24_P48204	SECTM1	NM_003004	0.140254572	0.150988085	0.128681398	0.13852923
A_24_P51322	FLG	NM_002016	0.099177477	0.086242487	0.135275238	0.117632283
A_33_P3268487	ABAT	NM_000663	0.137374703	0.174268966	0.136081065	0.1726279
A_33_P3321372		ENST00000276974	0.137193823	0.09299156	0.140847097	0.095467792
A_33_P3417695	ODF3B	NM_001014440	0.13315965	0.177177958	0.129702005	0.172577326
A_23_P161218	ANKRD1	NM_014391	0.128818812	0.110268373	0.149630681	0.128083247
A_33_P3309491	PTPRU	NM_005704	0.175755602	0.18233369	0.181412697	0.188202517
A_23_P14174	TNFSF13B	NM_006573	0.1266482	0.08067972	0.111607955	0.071098513
A_23_P69383	PARP9	NM_031458	0.124853108	0.120003683	0.116047643	0.11154023
A_33_P3340639	GPAM	ENST00000369425	0.088592016	0.085381281	0.117493484	0.113235307
A_23_P152782	IFI35	NM_005533	0.170210529	0.174095789	0.179079492	0.183167198
A_24_P170983	ESPNL	NM_194312	0.12152871	0.13805999	0.131286113	0.14914467
A_33_P3225512	OAS2	NM_002535	0.119949184	0.068715697	0.117481534	0.067302046
A_24_P28722	RSAD2	NM_080657	0.161984722	0.154118636	0.113655422	0.108136239
A_32_P164246	FOXQ1	NM_033260	0.114761158	0.161948418	0.199792213	0.28194237
A_23_P374082	ADAM19	NM_033274	0.177949196	0.172056095	0.199279951	0.192680444
A_33_P3423941	IFITM1	NM_003641	0.187782529	0.191663952	0.177729393	0.18140302
A_23_P384044	CNIH3	NM_152495	0.075645313	0.076072261	0.09467703	0.095211394
A_23_P137238	KDM5D	NM_004653	0.075608939	0.080132057	0.096802159	0.102593109
A_23_P207911	TRPV2	NM_016113	0.175054125	0.1795901	0.180050719	0.184716164
A_23_P62081	SCG5	NM_003020	0.106944059	0.104087552	0.133271848	0.129712118
A_23_P72737	IFITM1	NM_003641	0.182805981	0.193483891	0.171551829	0.181572371
A_23_P141624	KRTAP1-1	NM_030967	0.148978679	0.153591357	0.162922345	0.167966747
A_23_P33759	DHRS3	NM_004753	0.16764939	0.174399609	0.176336686	0.183436689
A_33_P3400374	HELZ2	NM_001037335	0.167078864	0.165988588	0.149246526	0.148272616
A_23_P386268	C4orf26	NM_178497	0.068240641	0.066964513	0.059859426	0.058740029
A_24_P274270	STAT1	NM_139266	0.141632397	0.140945769	0.150110633	0.149382903
A_22_P00002869		ENST00000568686	0.13860808	0.105525687	0.11179741	0.085114075
A_21_P0013565		ENST00000479351	0.137593718	0.143223978	0.145801607	0.15176773
A_23_P167367	PITX2	NM_153426	0.135109236	0.123588855	0.15339772	0.140317932
A_21_P0011609	LOC100130520	XM_005275830	0.062334113	0.089584925	0.073047502	0.104981921
A_23_P75786	SLC15A3	NM_016582	0.090627339	0.090345906	0.084119627	0.083858404
A_24_P296808	PNMAL1	NM_018215	0.088376184	0.078454932	0.110611468	0.098194047

A_23_P7313	SPP1	NM_001040058	0.118544854	0.111568807	0.148827887	0.140069764
A_23_P372478	SERPINA9	NM_175739	0.07880475	0.082028398	0.083535882	0.086953065
A_33_P3241661	LOC388780	NM_001287682	0.050214255	0.110576789	0.050381611	0.110945323
A_33_P3225522	OAS2	NM_001032731	0.109983193	0.101765664	0.106527204	0.098567893
A_23_P155755	CXCL6	NM_002993	0.072720546	0.045326677	0.085021281	0.052993719
A_23_P216655	TRIM14	NM_014788	0.046846721	0.040947184	0.042861252	0.037463616
A_21_P0003931	MIR143HG	ENST00000505254	0.07157637	0.061437517	0.08145217	0.069914403
A_23_P162171	MCAM	NM_006500	0.07038357	0.083867459	0.081695337	0.097346303
A_32_P387648	FLG	NM_002016	0.105379772	0.114552687	0.133732956	0.14537391
A_21_P0000996	LOC100505940	ENST00000418242	0.0451362	0.025822197	0.039320737	0.0224952
A_33_P3276062			0.043826364	0.06010026	0.046915787	0.064336868
A_19_P00322941	MIR143HG	ENST00000518014	0.06569079	0.042022593	0.07939995	0.050792383
A_33_P3258346	XAF1	NM_017523	0.065640137	0.053643772	0.068676922	0.056125555
A_23_P27306	COLEC12	NM_130386	0.06512148	0.057071941	0.078585609	0.068871795
A_23_P64828	OAS1	NM_002534	0.055236217	0.055099223	0.046153146	0.046038679
A_23_P112026	IDO1	NM_002164	0.031192934	0.021639897	0.023162889	0.016069105
A_19_P00324470	MIR143HG	NR_105059	0.050195102	0.046889499	0.058483625	0.05463218
A_24_P557479	XAF1	NM_017523	0.0465257	0.053807279	0.047157403	0.054537847
A_33_P3372910	DDX58	NM_014314	0.067113501	0.063424911	0.061650605	0.058262258
A_24_P295543	BLOC1S2	NM_001001342	0.079982377	0.074265751	0.085415919	0.079310938
A_23_P166797	RTP4	NM_022147	0.064730088	0.075874353	0.063416838	0.074335008
A_23_P30243	ERAP2	NM_022350	0.063390918	0.067525162	0.079023737	0.084177525
A_23_P46781	ITGA8	NM_003638	0.022376464	0.021836906	0.032164825	0.031389243
A_23_P39465	BST2	NM_004335	0.045896995	0.045785465	0.047675588	0.047559736
A_23_P17663	MX1	NM_002462	0.056255479	0.061787133	0.048380271	0.053137548
A_33_P3217700	USP9Y	NM_004654	0.013585616	0.013343755	0.015896961	0.015613952
A_33_P3220911	BST2	NM_004335	0.040844984	0.042863894	0.038869409	0.040790669
A_21_P0006594	TTTY15	NR_001545	0.011403283	0.011336441	0.012856293	0.012780934
A_23_P45871	IFI44L	NM_006820	0.027027868	0.020149855	0.025279116	0.018846123
A_33_P3401826	CMPK2	NM_207315	0.013502287	0.012889525	0.011525623	0.011002566
A_23_P201459	IFI6	NM_022873	0.028869084	0.029004079	0.028192659	0.028324491
A_33_P3224331	DDX3Y	NM_004660	0.001949524	0.001961927	0.002258727	0.002273098
A_24_P270460	IFI27	NM_005532	0.007984055	0.009015099	0.008135623	0.00918624
A_23_P324384	RPS4Y2	NM_001039567	0.000848908	0.000835762	0.001036575	0.001020522
A_23_P259314	RPS4Y1	NM_001008	0.000718386	0.000742254	0.000873112	0.000902121

Table S2 12h the ratio of Muse and non-Muse cells

Upregulated genes are shown in pink, and downregulated genes are shown in blue. G-CFF is shown as yellow.

ProbeID	PrimaryAccession	GeneSymbol	Comparison 5	Comparison 6	Comparison 7	Comparison 8
A_21_P0005616	NR_122034	LINC01419	64.93799917	49.23317609	22.3009926	16.9076459
A_33_P3337272	NM_001004354	NRARP	22.33670575	35.3448378	24.2028038	38.29768741
A_23_P102611	NM_003881	WISP2	20.31678951	19.77977762	16.87568561	16.42962872
A_23_P54996	NM_198393	TEX14	25.56940557	20.35075638	16.16257706	12.86383711
A_24_P250227	NM_021724	NR1D1	19.43937886	17.58724686	14.05370378	12.71470448
A_22_P00012795	NR_122034	LINC01419	50.74655457	43.97060702	13.09939771	11.35029706
A_23_P339818	NM_183376	ARRDC4	17.28717645	12.43293251	14.4248219	10.37432791
A_33_P3312676	ENST00000621996		37.41471182	22.26733556	50.15572832	29.85014125
A_33_P3359973	ENST00000436500		34.48524133	35.40206605	16.89627276	17.34547712
A_33_P3236392	NM_030916	PVRL4	24.60838565	21.02970277	20.77762066	17.75602808
A_24_P273489	NM_207372	SH2D4B	32.59846316	25.21974915	14.83304587	11.47556233
A_33_P6475164	NR_108090	LINC01447	16.96479342	12.02530058	9.731090723	6.89777282
A_23_P139500	NM_030762	BHLHE41	13.78821458	10.65070059	11.58774278	8.950947064
A_21_P0009266	ENST00000612725		32.12797586	22.27821612	13.92792905	9.657919777
A_33_P3229241	ENST00000369167	HIST2H2BF	13.26104543	8.577488965	10.73539858	6.943853965
A_23_P150549	NM_001079807	PGA3	22.45432623	15.30056824	17.43778173	11.88225229
A_21_P0014143	NR_125810	LINC01511	15.95780521	14.96146839	14.4103519	13.51063142
A_23_P89589	NM_002616	PER1	12.9882199	11.55034723	9.696881145	8.623379118
A_33_P3229122	NM_003522	HIST1H2BF	14.86269951	8.016619668	10.78247515	5.815834624
A_23_P137665	NM_001276	CHI3L1	11.38083051	10.98201319	11.75655618	11.34457234
A_22_P00010915	ENST00000512831		24.15410739	24.93776832	15.13830778	15.62945822
A_33_P3362311	NM_001010903	C6orf222	13.24543177	12.75255564	9.456499438	9.104613374
A_23_P321501	NM_182908	DHRS2	10.52525244	9.803764759	7.668175711	7.142535654
A_23_P410717	NM_144697	CIART	12.95212078	10.91727728	15.6364092	13.17985044
A_21_P0008282	NR_110074	LINC01467	17.47199905	18.55344649	17.23341307	18.30009298
A_22_P00015892	lnc-TCF19-1:4	lnc-TCF19-1	17.28128625	12.07225963	18.55864869	12.96459199
A_32_P128209	NR_026936	LINC01554	12.6338443	11.14183887	11.37892757	10.03512268

A_23_P96087	NM_006026	H1FX	10.51937221	9.455877001	7.538001787	6.775919353
A_24_P350397	NM_000862	HSD3B1	22.36850163	16.96820383	34.88666652	26.46418067
A_33_P3298216	NM_001198950	MYO16	16.0534972	10.40237608	12.82213025	8.308508697
A_33_P3229239	ENST00000369167	HIST2H2BF	11.56810289	8.066245904	9.466181448	6.600611012
A_33_P3795649	ENST00000447535		15.21586622	11.69616125	14.79144139	11.36991356
A_22_P00014914	ENST00000260126	SLCO5A1	11.02445703	8.731964706	8.246219256	6.53145051
A_23_P29257	NM_005318	H1F0	8.895139135	8.580903035	8.458080096	8.159283859
A_24_P256018	ENST00000415338		10.93847745	7.339916811	7.890464087	5.294644551
A_22_P00000694	ENST00000553791		18.89870903	16.7483877	13.18116002	11.68138935
A_33_P3344229	NM_003538	HIST1H4A	8.828160623	6.899907523	6.973256281	5.450152702
A_22_P00013727	NR_036494	LOC643201	18.57244745	14.2149637	6.347770027	4.858450711
A_24_P262127	NM_004165	RRAD	7.858576387	7.146043671	5.822510435	5.294586677
A_33_P3367939	NM_182973	TMPRSS9	18.26025703	16.10133888	12.71539681	11.21204991
A_33_P3375790	NM_001277397	RFPL4AL1	10.54000469	8.168725828	13.36627081	10.35914164
A_23_P15101	NM_024780	TMC5	13.93422594	11.82870065	15.79845102	13.41123279
A_23_P98350	NM_001165	BIRC3	8.478355012	7.831109055	8.562807336	7.909114205
A_23_P415411	NM_003545	HIST1H4E	8.468164503	5.676423267	6.317107852	4.234515989
A_22_P00014506	ENST00000420865		13.69391548	10.75180454	14.82680292	11.64129333
A_33_P3214105	NM_001674	ATF3	8.419228842	8.793286995	5.576231919	5.823978482
A_21_P0009764	ENST00000592431		10.24041494	9.545294003	8.634755924	8.048627368
A_33_P3839820	ENST00000589708	LOC101928538	17.37191936	10.22433124	19.33020126	11.37688799
A_22_P00003889	NM_020747	ZNF608	17.26348187	10.10135186	9.41442183	5.508644675
A_22_P00021404	ENST00000558010		17.21273165	21.04897	8.58711679	10.50094589
A_33_P3718734	NR_122070	LOC729083	8.557626401	8.527308531	6.898949952	6.874508423
A_23_P106002	NM_020529	NFKBIA	3.823506827	3.770956526	3.045732864	3.003872293
A_32_P98072	NM_007113	TCHH	16.8157646	18.892432	18.61410186	20.91285539
A_21_P0005727	NR_105006	LINC01111	8.402441788	6.10663304	7.73390599	5.62076204
A_33_P3839897	DW419002	RNU4ATAC	9.846787294	10.43953868	8.198402815	8.691925675
A_24_P260639	NM_005320	HIST1H1D	8.26621162	6.009169233	9.026202294	6.561648747
A_22_P00007695	lnc-HIST2H2AA3-1:1	lnc-HIST2H2AA3-1	8.249074654	8.858758887	6.575270429	7.061244782
A_23_P151975	NM_016321	RHCG	8.229688677	6.450543861	7.924481621	6.211318348
A_23_P500353	NM_021614	KCNN2	16.27495688	19.35621064	12.09466079	14.38448062
A_33_P3354151	NM_016074	BOLA1	8.156407278	6.019518004	8.434890447	6.225041636
A_33_P3229246	NM_003528	HIST2H2BE	9.572633952	8.907823512	7.112675613	6.61870697

A_23_P380318	NM_001965	EGR4	7.828087114	7.558867227	4.916651412	4.747560251
A_23_P145569	NM_153187	SLC22A1	9.502850133	7.866243293	7.294561895	6.038272495
A_33_P3281273	NM_003775	S1PR4	15.86251496	8.667785792	8.397612012	4.588723936
A_33_P3341722	CU692082		7.103588152	6.110698798	6.641211106	5.712949548
A_21_P0013007	ENST00000510155	LOC643201	12.21941939	8.493359926	6.42534767	4.466070658
A_23_P150609	NM_000612	IGF2	7.67699516	9.182852651	8.217935766	9.829899806
A_23_P50368	NM_206818	OSCAR	7.920619971	6.854710104	8.452816568	7.315286852
A_24_P151582	NM_003216	TEF	12.06783034	7.991921517	11.13262493	7.372581664
A_19_P00320881	NR_040025	LINC01539	9.248440263	7.602211602	9.134630782	7.508660286
A_33_P3692979	AK093862	LOC283485	9.244657325	9.126487832	9.702942741	9.578915232
A_21_P0013141	ENST00000448991		11.99475416	11.8816727	7.085454951	7.018656284
A_23_P18672	NM_020973	GBA3	11.8227543	12.77744508	14.49490855	15.66537653
A_23_P167997	NM_003518	HIST1H2BG	7.451987757	5.928451706	6.680371033	5.314589656
A_23_P312150	NM_001956	EDN2	11.62335577	8.325273176	9.013839914	6.45619743
A_23_P397293	NM_017527	LY6K	7.351118077	5.166756106	5.896289942	4.144225646
A_24_P788878	NM_001007595	C2CD4B	11.2823601	8.515036953	8.988526226	6.783831778
A_21_P0000795	NR_038423	LINC00624	11.19500318	11.93740893	15.85997806	16.91174541
A_23_P365738	NM_015193	ARC	7.415374322	8.405535786	6.580318616	7.458976609
A_23_P421379	NM_000612	IGF2	14.15294193	17.42186481	12.69856149	15.63156428
A_23_P46936	NM_000399	EGR2	7.113210915	7.614217055	4.977314897	5.327883066
A_21_P0010327	lnc-ICOSLG-5:2	lnc-ICOSLG-5	14.05079655	12.44500239	6.927182114	6.135509659
A_22_P00004211	NR_125810	LINC01511	11.06455166	9.205807404	9.692463075	8.06421725
A_23_P6596	NM_005524	HES1	8.558783593	10.74797504	6.104222061	7.665578368
A_33_P3270315	BC009492		8.551255771	7.504165845	5.594149798	4.909153576
A_22_P00006427	NR_104624	LOC101929181	11.02935831	10.06294215	9.473788827	8.643675016
A_19_P00803685	NM_001164440	ANKRD33B	7.308234471	7.349127015	6.520256412	6.556739899
A_24_P229234	NM_015011	MYO16	10.98605678	8.315910314	8.965733825	6.786624164
A_21_P0011967	NM_003014	SFRP4	10.98258761	10.78607157	10.32772035	10.14292212
A_33_P3366127	NM_001277397	RFPL4AL1	10.98066347	8.726354042	12.59493138	10.00921581
A_22_P00015968	ENST00000522960		10.93943748	9.455886052	9.958834346	8.60826738
A_24_P33895	NM_001040619	ATF3	7.023255311	8.078373664	5.666496849	6.517786537
A_24_P55148	NM_021058	HIST1H2BJ	7.218785617	6.081577849	7.40987632	6.242565174
A_33_P3410836	NM_003539	HIST1H4D	6.968103388	5.020375897	6.58935876	4.747498143
A_23_P58770	NM_004821	HAND1	10.7846025	6.287251503	8.072088207	4.705898871
A_32_P50223	NM_001243079	PLK5	13.62237705	9.736837348	6.442744831	4.605066962

A_23_P359540	NM_003540	HIST1H4F	6.86815584	5.51637622	6.156876186	4.945089508
A_23_P368909	NR_027701	LINC00346	7.074507855	7.202659537	8.851673539	9.012017818
A_33_P3299865	NM_003541	HIST1H4K	6.276999634	4.883066792	5.746191658	4.470135304
A_33_P3243449	NM_001252	CD70	7.053688863	4.59988378	5.238810806	3.416357216
A_24_P20873	NM_003495	HIST1H4I	6.814612339	5.240830862	5.978950753	4.59815879
A_33_P3300591			13.27835575	12.01028902	20.73339635	18.75338237
A_22_P00009461	lnc-MAEA-2:1	lnc-MAEA-2	13.26975566	7.549586678	9.914063292	5.640426401
A_21_P0009625	NR_110763	LOC100996324	8.143023728	6.710578279	9.262758199	7.633339414
A_21_P0011517	NM_000526	KRT14	8.117181562	8.428166631	9.031522644	9.377537905
A_23_P436281	NM_001034077	HIST2H4B	6.212452369	4.593549045	5.779274582	4.273253082
A_23_P93169	NM_016571	LGSN	13.05552641	12.16860075	14.04110047	13.08721994
A_21_P0010777	NR_038423	LINC00624	13.05008077	13.03136606	14.20250328	14.18213592
A_23_P431933	NM_032294	CAMKK1	8.003022571	4.77038175	7.884108336	4.699500244
A_23_P307310	NM_013227	ACAN	10.25224531	9.436997376	8.176804774	7.526593719
A_23_P323685	NM_003543	HIST1H4H	6.143301583	5.443478526	6.008009884	5.323598776
A_33_P3212394	NM_001124759	FRG2C	12.82576903	11.44056284	19.4602667	17.3585228
A_33_P3345534	NM_000526	KRT14	7.94613561	8.514099474	8.561773691	9.173741358
A_22_P00019692	lnc-HIST4H4-1:1	lnc-HIST4H4-1	7.892025528	6.481389877	5.974667837	4.906744346
A_24_P291231	NM_016831	PER3	10.09442527	8.190956366	8.560050309	6.945912885
A_32_P33083	NM_016378	VCX2	12.34048634	11.35264471	5.579631818	5.132988755
A_32_P62963	NR_029392	KRT16P2	7.646087606	8.481134369	8.780216067	9.7391236
A_33_P3357530	NM_006598	SLC12A7	5.903119159	5.28963384	3.920751486	3.513284956
A_33_P3532770	AK092120	LOC283674	9.631079452	6.236883509	9.790739342	6.34027588
A_24_P887857	ENST00000420566		7.48936059	7.824984429	8.629305147	9.016013797
A_33_P3422233	NM_020894	UVSSA	6.474253613	5.345128714	4.843521403	3.998799997
A_33_P3315959	NM_016571	LGSN	11.84638311	11.64043892	17.61502817	17.30879861
A_23_P30799	NM_021018	HIST1H3F	6.235712227	4.401053016	6.201344877	4.376797161
A_23_P151059	NM_018088	FAM90A1	9.398374151	8.82108091	7.95282305	7.464322494
A_22_P00016430	ENST00000554254		6.210043559	4.726690193	5.301311837	4.035021403
A_23_P70307	NM_022138	SMOC2	9.359163038	11.43189626	8.47039147	10.34629231
A_23_P97112	NM_000450	SELE	6.385359004	5.5606455	6.180016917	5.381824771
A_24_P49183	NM_017820	EXD3	11.6290184	8.158894221	38.15645344	26.77048542
A_33_P3411744	NR_004428	EGOT	7.325012506	5.271423361	7.145018073	5.141890905
A_33_P3404032	NM_033445	HIST3H2A	9.229025287	6.670055646	7.364187725	5.322289234
A_33_P3265539			11.45365822	10.81271207	24.37633719	23.01223857

A_24_P217834	NM_003530	HIST1H3D	5.653233157	4.223660116	5.42035512	4.049671594
A_21_P0014444	NR_121625	LOC100507639	11.42961641	8.649732334	9.319148772	7.052567609
A_23_P429998	NM_006732	FOSB	6.098139762	7.482981536	5.415883059	6.645789457
A_33_P3261298	NM_014228	SLC6A7	7.227029853	4.935701655	6.978927948	4.766260403
A_23_P131676	NM_020311	ACKR3	6.212143593	8.286860169	5.9684977	7.961842015
A_19_P00316340	NR_109780	LOC100506860	6.200639104	4.374530991	4.958324968	3.498082354
A_22_P00001177	ENST00000554926		11.10733197	8.56952541	9.988105721	7.706020312
A_21_P0006734	ENST00000419836		11.10581129	9.919917158	21.85482884	19.52113951
A_32_P76627	ENST00000417510		7.088165864	7.306743165	8.303949745	8.560018094
A_23_P395374	NM_003539	HIST1H4D	5.965446572	4.74540607	6.034159871	4.800066271
A_23_P30813	NM_003541	HIST1H4K	5.957186906	3.55083213	5.779557749	3.444954753
A_23_P94472	NM_003837	FBP2	8.88631368	6.131357211	5.47302293	3.776263118
A_32_P74409	NM_001145033	C11orf96	5.476512285	5.169658396	3.802463084	3.589407672
A_23_P329340	ENST00000261383		10.93112957	10.35029267	8.80615308	8.338229009
A_23_P376488	NM_000594	TNF	6.091461917	4.889979202	4.318207649	3.466482411
A_33_P3379391	NM_177925	H2AFJ	8.786335138	6.521683177	8.769555159	6.509228188
A_23_P209320	NM_022817	PER2	6.963698674	5.671563988	4.858985016	3.957386114
A_21_P0014172	BC010926	HIST1H4H	5.438373724	5.208748502	5.195299864	4.975937984
A_32_P135348	NM_033394	TANC1	6.054116166	4.680350841	5.409768851	4.182215123
A_23_P51936	NM_001561	TNFRSF9	6.945808516	4.535570373	5.084357701	3.320054404
A_33_P3378880	ENST00000377803	HIST1H4C	6.028180412	4.770350704	6.154561406	4.870361258
A_33_P3393766	NM_001130677	C17orf96	6.914261254	4.133304682	5.517571323	3.298371661
A_33_P3273143	ENST00000406566		8.692515734	8.973967394	10.95886284	11.31369569
A_33_P3331831	ENST00000612156		8.688617814	9.216084499	10.93184349	11.59549143
A_23_P130753	NM_001352	DBP	8.687770158	6.321680374	9.456858942	6.881309989
A_21_P0006504	XR_426531	LOC102723911	8.667605157	6.390909452	9.615567254	7.089872986
A_23_P7976	NM_005321	HIST1H1E	5.816502024	4.288895548	5.854405519	4.316844327
A_33_P3697530	NM_001142287	SEMA4D	8.644771519	9.850296841	5.850898739	6.666814644
A_24_P62783	NM_004102	FABP3	10.68028535	13.12610984	12.99037527	15.96521882
A_21_P0014532			10.6782714	11.17427397	6.679387838	6.989643437
A_23_P128215	NM_003877	SOCS2	5.980540767	4.924754396	5.527585992	4.551762872
A_24_P241183	NM_013269	CLEC2D	6.795741058	6.401486364	6.834210326	6.437723839
A_33_P3287879	NM_003536	HIST1H3H	5.923715539	4.241190614	6.303267464	4.512937637
A_23_P355439	NM_170745	HIST1H2AA	6.785822687	5.826064226	5.603003097	4.810537706
A_33_P3404989	NM_003536	HIST1H3H	6.771834603	4.832499669	8.369309546	5.972485742

A_23_P23279	NM_052862	RCSD1	6.716387103	7.223173354	9.29579671	9.997212798
A_23_P420863	NM_022162	NOD2	10.34191678	9.872810641	8.343773487	7.965302505
A_21_P0007899	NR_110036	LOC100996671	8.391555231	10.11806317	5.62863391	6.786688749
A_24_P414658	ENST00000359193	HIST1H2AG	8.352433321	8.476782151	8.541735719	8.668902833
A_33_P3397323	NM_152355	ZNF441	5.81822503	5.010776056	5.445183312	4.689504793
A_33_P3382031	NM_001098844	TMEM236	10.26761573	6.826925988	11.87099513	7.893011126
A_23_P149545	NM_003528	HIST2H2BE	6.632897263	6.027577977	5.882424627	5.345593596
A_24_P82419	NM_001013699	H3F3C	2.984299208	2.721295382	2.674656025	2.438940797
A_33_P3569068	NM_003550	MAD1L1	5.769911056	4.63321296	4.421469162	3.550420106
A_33_P3383955	NM_001300734	DDB2	6.59873158	5.999483333	5.973609486	5.431130228
A_23_P168828	NM_005655	KLF10	5.58143022	4.944352327	5.595059147	4.956425616
A_21_P0011983	ENST00000432711		6.576335371	5.16796188	6.607486507	5.192441757
A_21_P0010596	ENST00000453464	RNF223	6.574260601	4.244943149	9.932210374	6.413142244
A_33_P3335910	NM_033071	SYNE1	6.565782823	5.528005048	5.773669971	4.861092364
A_33_P3288942	NM_001282695	FAM107B	6.555266912	5.11118707	4.925777935	3.840663215
A_33_P3252394	NM_006705	GADD45G	5.711672862	7.487293631	4.643447465	6.086982828
A_33_P3242543	NM_001270458	MAOA	8.154296035	5.809362468	6.516430185	4.642498234
A_23_P205046	NM_017664	ANKRD10	6.483498569	8.044150116	7.43415656	9.223642253
A_33_P3363355	NM_022377	ICAM4	5.637350086	5.300122444	3.395955943	3.192809039
A_33_P3213468	NM_001164440	ANKRD33B	9.819248623	8.100172907	7.092298276	5.850635272
A_23_P93258	NM_003537	HIST1H3B	5.438091032	3.758068754	5.248593456	3.627113807
A_33_P3324974			7.970802724	8.181534853	7.699563567	7.903124673
A_33_P3225948	NM_001042478	AJAP1	6.386218699	5.742567577	7.235511082	6.50626189
A_23_P17103	NM_025244	TSGA10	7.947524545	6.718036473	7.864339839	6.647720504
A_23_P34915	NM_001040619	ATF3	2.923423665	3.026668243	2.554159458	2.644362981
A_32_P145867	NM_006188	OCM2	9.698498914	8.789115012	9.808119322	8.888456815
A_23_P59045	NM_021052	HIST1H2AE	5.402894697	4.619156377	5.665708559	4.843846731
A_23_P38537	NM_005557	KRT16	7.900746283	8.912662796	11.61230913	13.09959742
A_33_P3229083	NM_080593	HIST1H2BK	5.389251916	4.635066594	4.797406282	4.126045311
A_33_P3215640	NM_153370	PI16	6.329977347	6.528025367	6.264271208	6.46026346
A_21_P0013116	NR_028093	LPAL2	34.14431761	23.26764161	6.50045223	4.429732481
A_23_P39237	NM_003407	ZFP36	4.980159814	5.457137679	4.545607239	4.980965564
A_23_P70670	NM_004233	CD83	4.964987181	4.591705937	4.531236947	4.190566225
A_33_P3367361	NM_001282695	FAM107B	5.490794588	4.06335075	5.381689734	3.982609924
A_23_P134237	NM_002889	RARRES2	9.488854259	10.3638016	16.19285214	17.68596106

A_21_P0014724	XR_251947	LOC100507336	6.230329842	5.3374604	7.737567083	6.628695262
A_33_P3228450	NM_001136007	FXYD3	9.460642141	12.34785909	25.91750456	33.82705841
A_33_P3365087	NM_001130404	PRR20B	33.21250737	21.84146099	20.23423816	13.30659316
A_23_P93180	NM_003526	HIST1H2BC	5.434913847	4.754476688	4.995664529	4.370220249
A_24_P146211	NM_021063	HIST1H2BD	5.269235527	4.55552938	4.830830992	4.176505757
A_23_P124108	NM_000632	ITGAM	7.648774303	6.632617504	10.7207612	9.296484059
A_22_P00005863	ENST00000433197	ERN1	5.24979524	4.458955816	3.380226204	2.871022317
A_23_P161156	NM_182755	ZNF438	5.378906427	4.481741046	4.967095051	4.138617035
A_23_P363174	NM_003511	HIST1H2AL	6.097098414	4.150756077	4.967463895	3.381728414
A_33_P3249534	NM_005382	NEFM	5.358657248	6.580311327	3.7101763	4.556013569
A_22_P00014004	ENST00000568143		9.180045668	8.846625262	16.61393662	16.01051637
A_23_P133842	NM_005323	HIST1H1T	9.176501225	7.408622022	5.282964535	4.26518631
A_32_P52153	NR_038453	UNC5B-AS1	6.071588989	5.80300646	7.220932691	6.901507848
A_33_P3400248	NM_019851	FGF20	7.523835664	7.920733524	9.810228206	10.32773799
A_23_P42868	NM_000596	IGFBP1	5.328590988	4.805828725	5.075339527	4.577422539
A_23_P144337	NM_012118	CCRN4L	6.053183428	5.354494829	4.176183077	3.694147213
A_32_P226205	NM_033400	ZFHX2	5.31705479	4.359207173	4.535333871	3.718310365
A_33_P3347320	BC017676	ADIRF-AS1	7.479678433	6.253869755	5.901084457	4.933983986
A_24_P10137	NM_014059	RGCC	6.003897785	5.718086467	8.818140152	8.398358811
A_23_P111041	NM_003525	HIST1H2BI	5.134965342	4.507770736	4.557282424	4.000647128
A_33_P3265394	ENST00000538098	WDR74	9.043680897	9.887759279	8.988332676	9.827245214
A_23_P122443	NM_005319	HIST1H1C	4.785552036	3.867526363	4.73285598	3.824939137
A_22_P00008199	ENST00000519803		9.016979423	7.263489507	13.80894797	11.12358629
A_33_P3229301	NR_027003	DKFZp434J0226	8.991517161	11.16870212	10.76044403	13.36595281
A_23_P70448	NM_005325	HIST1H1A	5.262207675	3.317593674	7.609353593	4.79736736
A_19_P00317789	NR_015433	LOC93622	5.25855107	4.373149364	4.776697357	3.972427144
A_21_P0013364	NR_034163	ZNF890P	30.95520419	23.70567891	15.73379122	12.04903061
A_33_P3316539	NM_001008539	SLC7A2	5.962060131	4.718160107	4.138464258	3.275031875
A_23_P312920	NM_006235	POU2AF1	8.945341094	7.888672093	5.792417296	5.108187625
A_24_P303454	NM_012454	TIAM2	5.232883676	4.24985887	4.711358332	3.826304813
A_22_P00014305	NR_104064	SMIM2-AS1	8.911490095	6.050841648	10.33523979	7.017558083
A_21_P0000646	NR_034148	LINC00900	7.325394253	4.349152576	8.734290283	5.185626844
A_32_P3534	NM_001167902	PGPEP1L	30.54545692	21.44404251	17.85685512	12.53617392
A_23_P377839	NR_026552	LINC00161	5.918322854	6.191310963	7.523847774	7.870892204
A_33_P3364038	NM_001145805	IRGM	7.279154266	6.121023916	9.093745528	7.646909494

A_33_P3220837	NM_005461	MAFB	5.041504207	4.109593981	3.86488948	3.150473727
A_33_P3301709	NM_001098722	GNG4	7.220577569	7.272459867	7.966657026	8.023900157
A_23_P97700	NM_006472	TXNIP	4.67778491	3.568768831	4.756742032	3.629006683
A_23_P381368	NM_002148	HOXD10	7.202792046	7.107804013	8.196623068	8.088528721
A_23_P102364	NM_019850	NGEF	5.129739699	4.908710432	3.698848125	3.539472847
A_23_P307502	NR_024274	CDKN2A-AS1	5.798828587	5.060298976	5.259553195	4.589704843
A_24_P13001	NM_014978	SORCS3	29.26530637	26.27959084	29.02704894	26.06564099
A_33_P3271684	NM_001098844	TMEM236	8.658140358	5.24731093	7.878082173	4.774552616
A_23_P62901	NM_006763	BTG2	5.11283032	5.095626688	5.504858989	5.48633626
A_23_P16523	NM_004864	GDF15	4.963480159	5.044230064	3.36699586	3.421772869
A_23_P254863	NM_153361	NIM1K	8.606665412	4.659459781	15.192986	8.225149208
A_23_P61149	NM_001017915	INPP5D	7.089058609	8.663720959	7.392589518	9.034673894
A_33_P3298159	NM_000954	PTGDS	5.731713924	5.464692383	6.862725166	6.543013563
A_33_P3340852	ENST00000427543		8.543038375	5.447263806	8.204244565	5.231240048
A_33_P3868357	ENST00000507936		5.06069887	4.442530386	4.210621554	3.696290706
A_24_P3783	NM_003521	HIST1H2BM	4.894903299	4.185786408	4.577577817	3.914431366
A_23_P54488	NM_015162	ACSBG1	8.458792065	8.960865239	5.064983571	5.365616611
A_23_P116653	NM_138722	BCL2L14	8.455344349	7.540147347	6.65482811	5.934516969
A_33_P3881812	ENST00000520549	IRGM	5.682331116	5.591164661	6.603817626	6.497866982
A_33_P6474388	NR_120540	LOC101928053	5.674483093	3.933859567	4.78357638	3.316234695
A_33_P3344127	NM_003512	HIST1H2AC	5.011127411	3.876634572	4.323255163	3.344492976
A_23_P121120	NM_023915	GPR87	8.42227719	6.707722449	24.21353858	19.28429718
A_33_P3293164	NM_001275	CHGA	6.946631394	5.711358083	4.761119083	3.914480907
A_23_P51126	NM_016232	IL1RL1	5.637092686	5.294508836	6.756600422	6.345980566
A_32_P221799	NM_003514	HIST1H2AM	5.618827435	4.02817785	5.171005993	3.707131433
A_22_P00008339	XR_242926	LOC101928554	6.872265516	6.667475539	6.585803207	6.389549659
A_32_P14721	NM_001291661	DNAH12	5.587594266	5.995499414	4.700397833	5.043535931
A_23_P9614	NM_020142	NDUFA4L2	6.853896351	6.996641002	6.516647684	6.652368529
A_33_P3421913	NM_001301043	CADM1	5.582748386	5.256051476	4.772550629	4.49326569
A_33_P3421053	NM_000450	SELE	6.83480194	5.539447917	8.459100463	6.85590407
A_32_P141238	NM_001278597	ANO2	8.22643959	8.690440462	8.798946987	9.295239342
A_33_P3211608	NR_104167	IL1RL1	26.8757229	29.90268944	8.463987876	9.417272305
A_23_P167983	ENST00000314088	HIST1H2AC	4.770398121	4.050672144	5.503477766	4.673149603
A_23_P111054	NM_021062	HIST1H2BB	4.894918276	4.209362028	5.103043248	4.388338123
A_21_P0007784	NR_110034	LOC100996671	8.158440263	9.580900464	4.793774889	5.629590776

A_21_P0012528	ENST00000601814		5.508931775	5.744673484	5.355019802	5.584175212
A_23_P109488	NM_052880	PIK3IP1	8.109703292	8.010707805	4.500038261	4.445106106
A_21_P0009159	ENST00000436469		5.478179226	5.494004802	5.779713682	5.796410342
A_22_P00025133	ENST00000454307		8.07457382	6.307669975	31.60475368	24.68890127
A_22_P00025235	ENST00000509666		8.066550915	8.866658718	8.499154847	9.342171916
A_23_P350005	NM_173553	TRIML2	5.451407172	7.215896705	5.826453869	7.712337007
A_23_P103486	NM_000775	CYP2J2	8.022931439	5.261599006	6.694641319	4.390479761
A_22_P00015999	ENST00000431401	LOC101928106	8.007603865	5.963748938	6.449563564	4.803381699
A_33_P3376249	NM_005978	S100A2	4.821564511	4.364720543	5.039068997	4.561616445
A_33_P3316278	NR_003569	ABCC6P1	25.85222277	25.57048721	10.77889547	10.66142789
A_33_P8915099	NR_110396	LOC101928336	6.567951032	6.608731722	6.657718383	6.699056442
A_23_P17065	NM_004591	CCL20	4.351922675	4.981332471	4.892944429	5.60060111
A_23_P135669	NM_030958	SLCO5A1	6.560518117	6.830414598	6.453120769	6.718598976
A_22_P00023723	NM_001278587	LOC100134391	25.0633603	25.46234387	11.01684789	11.19222506
A_22_P00002038	lnc-BHLHE41-1:1	lnc-BHLHE41-1	7.818662585	8.055948687	18.31447864	18.87029892
A_23_P326760	NM_015460	MYRIP	6.502948141	6.830392784	12.59418608	13.22834442
A_22_P00022660	NR_046693	STARD13-AS	6.5011733	5.157266479	7.90413992	6.270215232
A_23_P59069	NM_003527	HIST1H2BO	4.587173236	4.149971918	4.578353839	4.141993093
A_19_P00316705	lnc-MMRN1-2:6	lnc-MMRN1-2	24.70122743	27.26657995	24.91399748	27.50144729
A_21_P0009009	ENST00000596416		6.454500099	4.32349153	7.137487196	4.780984579
A_23_P502750	NM_002392	MDM2	5.289127669	5.842143921	4.804159494	5.306468844
A_23_P151895	NM_003613	CILP	24.46220615	25.98230852	6.005885592	6.379096451
A_23_P25396	NM_005123	NR1H4	6.419599022	6.817149196	8.54251154	9.071528529
A_33_P3294524	NM_001204056	ANKRD12	5.258417889	4.074112714	5.387478434	4.174106137
A_23_P12199	NM_052943	FAM46B	6.382218509	4.904805516	6.381167047	4.903997456
A_24_P89891	NM_005658	TRAF1	4.660962708	3.260873987	4.229482032	2.959004137
A_23_P156708	NM_032470	TNXB	6.368140659	6.108713494	4.966682215	4.764348072
A_24_P9321	NM_003533	HIST1H3I	4.651413376	4.037317723	4.447002938	3.859894256
A_23_P74278	NM_001037341	PDE4B	5.217538796	3.904224753	5.81588017	4.351956777
A_23_P122439	NM_181531	BTN2A2	5.20811166	4.148823507	4.795808536	3.820379533
A_21_P0014515	NR_125858	LOC101928461	7.561373397	8.733010869	7.363126679	8.504045752
A_33_P3422043	NM_183375	PRSS48	23.68935357	18.13331366	6.275488038	4.803651253
A_23_P309381	NM_001040874	HIST2H2AA4	4.488324148	3.653484936	4.113168343	3.348109024
A_33_P3228460	NM_001136007	FXYD3	7.532012385	7.230237702	7.769726898	7.458428037
A_22_P00022297	ENST00000521681		7.514024443	6.619457629	9.917184614	8.73651448

A_24_P882732	ENST00000300992		5.155259809	5.609622377	6.39835669	6.962280504
A_23_P163278	NM_017851	PARP16	5.144585239	4.50255719	5.064263821	4.432259632
A_21_P0002605	XR_425445	LOC102724744	23.2593581	21.39517993	13.76025062	12.65740167
A_23_P42198	NM_003534	HIST1H3G	4.455882545	3.575926091	5.253531521	4.216053777
A_23_P431330	NM_175918	CRIPAK	5.133900853	4.359712557	4.210185145	3.575292466
A_23_P395404	NM_019851	FGF20	7.444719883	7.51997445	9.118701772	9.210877698
A_33_P3887081	NR_033890	LINC01252	7.437716613	5.527174937	5.798837251	4.309277913
A_23_P8013	NM_003519	HIST1H2BL	4.168440521	3.76759582	4.433578298	4.007237474
A_32_P95960	NM_001004419	CLEC2D	7.421644396	6.727882983	4.818191619	4.367796093
A_33_P3336397	NR_104614	LOC100506688	23.05279869	23.00793121	9.89696332	9.877700937
A_23_P30805	NM_021968	HIST1H4J	4.434628537	3.764052807	3.764018026	3.194847662
A_33_P3409746	NM_001166692	C11orf91	6.196477143	5.300079569	6.138570841	5.250550134
A_22_P00021182	lnc-LRRC40-2:2	lnc-LRRC40-2	5.071023567	6.070804212	4.856026541	5.813419321
A_24_P158946	NM_139241	FGD4	5.067050977	5.875411705	4.938346468	5.726174608
A_22_P00017797	ENST00000569134		6.14219058	5.439538089	4.519075588	4.002103723
A_23_P345118	NM_002648	PIM1	4.397391234	4.617781992	4.339396156	4.556880286
A_33_P3387646	NR_036494	LOC643201	6.131075025	5.14238354	4.630359007	3.883671598
A_24_P331128	NM_002068	GNA15	22.50156768	20.39834597	15.38155272	13.94383886
A_22_P00000215	NR_110542	PIK3IP1-AS1	7.280010043	5.306413024	7.313912819	5.331124822
A_24_P39211	NM_198219	ING1	5.018335996	5.261203804	4.571091389	4.79231431
A_23_P6293	NM_018961	UBASH3A	22.22424223	24.88459772	11.02525194	12.345031
A_21_P0013154	ENST00000417483		4.469221048	4.654374382	6.408912229	6.67442415
A_23_P17345	NM_005461	MAFB	4.466258341	4.009739244	3.871400193	3.475684588
A_33_P3356816	NM_178349	LCE1B	7.198523847	5.678867206	23.98788893	18.92388477
A_33_P3358626	NM_001293725	TAF4B	4.982987526	4.014712179	5.192733992	4.183701503
A_21_P0006440	ENST00000438499		7.151463604	6.140790988	7.053037627	6.056274953
A_23_P4773	NM_006840	LILRB5	21.73943386	22.61521974	13.40848414	13.94865281
A_33_P3223592	NM_001302688	APOE	4.422078434	4.948192635	5.031755689	5.630405886
A_33_P3229067	NM_003520	HIST1H2BN	4.948605721	4.65074933	4.639272952	4.360035289
A_33_P3354374	NR_040018	C1QTNF1-AS1	5.981084926	4.379995913	5.358361381	3.923970524
A_23_P41765	NM_002198	IRF1	4.41696265	4.014508259	3.382576065	3.074370472
A_33_P3265359	NM_018645	HES6	4.938365652	4.294792937	3.77919515	3.286686686
A_33_P3304748			4.93811218	4.532788614	4.662017435	4.279355912
A_23_P81158	NM_000669	ADH1C	5.959974943	5.898172393	4.605809187	4.558048793
A_33_P3741059	ENST00000456414		5.948484776	4.607685625	5.721197333	4.43162918

A_22_P00003688	ENST00000507600		7.05884043	7.430436605	18.52174524	19.49677928
A_21_P0005135	NR_125867	LOC102723649	5.928070954	4.635737914	7.193498611	5.625299444
A_21_P0011018	XR_246466	LOC101928620	5.91129852	5.556667504	4.262114074	4.006421041
A_22_P00009529	NR_110387	LOC101927501	21.13685483	18.08320773	8.649069075	7.39953574
A_23_P390068	NM_173481	MISP	5.879395888	6.006410947	6.182710051	6.316277734
A_21_P0004055	NR_104999	LOC102467226	6.972713382	5.380209373	9.263197272	7.147567677
A_33_P3392525	NM_001661	ARL4D	4.348235193	4.125608124	4.165820068	3.952532545
A_33_P3441031	BC038732	LINC00944	6.966264226	8.17298265	5.861410018	6.876742085
A_33_P3745146	NM_001098517	CADM1	6.954668175	4.861413977	10.26196584	7.173263043
A_33_P3352522	AK092862	GAS6-AS1	6.949389423	8.99292188	5.54499029	7.175546148
A_32_P133072	NM_006108	SPON1	6.948270921	7.104602016	4.495223636	4.596362933
A_33_P3269636	NM_001166034	SBSN	4.225237088	4.384192158	3.598558172	3.733937336
A_33_P3286273	NM_001199938	EBLN1	6.924490178	6.905769128	9.87327896	9.846585565
A_24_P623782	NM_001005217	FRG2	6.910862715	7.451990401	21.16223664	22.81926162
A_21_P0007476	NR_027358	LINC00592	6.903750349	5.061650559	8.575812295	6.287562976
A_23_P366216	NM_003524	HIST1H2BH	4.20545544	3.919779125	4.33438708	4.039952448
A_33_P3406047	NM_134444	NLRP4	5.78193151	5.60607858	5.901588522	5.722096318
A_32_P36235	NM_004907	IER2	2.473281312	2.473281312	2.198441031	2.198441031
A_23_P382775	NM_014417	BBC3	4.179514405	5.672266584	3.900731842	5.293914254
A_32_P70158	NM_006864	LILRB3	6.834295578	5.41083997	6.845950925	5.420067727
A_24_P166407	NM_003544	HIST1H4B	3.930777867	3.474882782	3.729833967	3.297244533
A_33_P3651911	ENST00000560295		5.754442705	6.218571822	4.01392554	4.337671873
A_33_P3356821	NM_178351	LCE1C	20.23911182	20.81606751	20.61451898	21.20217639
A_33_P3422010	NM_001291661	DNAH12	6.802170016	6.836644221	4.937764606	4.962789783
A_33_P3230269	NM_198182	GRHL1	5.73124164	5.447602847	8.391223767	7.975942624
A_22_P00001368	lnc-AP002478.1-2:2	lnc-AP002478.1-2	5.730745706	5.81638739	5.924279051	6.012812945
A_23_P112554	NM_001855	COL15A1	5.724398422	6.754279908	7.544087158	8.901350424
A_23_P398566	NM_173200	NR4A3	4.259660001	3.936937062	3.550727634	3.281715259
A_33_P3247332	THC2686537		19.99777458	15.05495801	21.42735588	16.13119209
A_33_P3399571	NM_004666	VNN1	6.758878056	7.046948278	6.696974009	6.982405818
A_22_P00009245	lnc-LRMP-2:1	lnc-LRMP-2	5.700398921	4.655968925	7.695308853	6.285370443
A_33_P3280905	ENST00000461229		6.746376643	6.000774486	6.219384602	5.532025028
A_33_P3270514	NR_003679	HAND2-AS1	6.731746388	4.679154703	7.060174426	4.907441022
A_33_P3813128	NM_004186	SEMA3F	5.671658166	6.041315436	4.654387551	4.957742962

A_23_P164258	NM_016518	PIPOX	5.671519911	6.496315515	4.937669534	5.655743029
A_24_P41801	NM_005577	LPA	6.711857133	6.152316273	6.662397906	6.106980265
A_22_P00015718	lnc-SYT16-1:1	lnc-SYT16-1	6.684556074	5.624539613	6.942672109	5.841724397
A_24_P241815	NM_002229	JUNB	4.210340581	4.216602036	2.960420699	2.964823321
A_23_P19619	NM_002114	HIVEP1	4.207364443	3.641344458	4.364347872	3.777208786
A_22_P00022787	NR_104624	LOC101929181	4.668190603	4.539418793	4.892132904	4.757183656
A_23_P145074	NM_006813	PNRC1	4.080754019	3.801217704	3.950424696	3.679816089
A_23_P52610	NM_000107	DDB2	4.075535209	3.577507683	4.359632776	3.826888727
A_21_P0004928	lnc-NT5DC1-4:1	lnc-NT5DC1-4	5.573833654	5.516064341	9.029336725	8.935753276
A_22_P00020093	ENST00000429352		6.585374433	6.528149481	5.070847294	5.026783135
A_23_P90419	NM_025245	PBX4	5.568440365	4.652259542	4.954025442	4.138934894
A_21_P0006028	NR_120623	TCERG1L-AS1	4.625277194	4.252690675	6.4527693	5.932970215
A_21_P0009110	ENST00000611381	ZFP90	6.548500425	5.435902548	5.653863004	4.69326507
A_23_P94800	NM_002961	S100A4	4.604534121	4.796115011	5.400692457	5.625399115
A_23_P328074	NM_002968	SALL1	5.476955976	5.137696192	5.315626129	4.986359621
A_22_P00002982	ENST00000562471		5.471213189	4.73155042	4.623868653	3.998759856
A_33_P3303810	NM_005558	LAD1	6.444057659	5.366686765	5.949649423	4.95493779
A_21_P0011750	ENST00000455211		6.421879837	4.822363471	6.499739979	4.88083076
A_19_P00321383	ENST00000447430		5.443443268	6.822077293	4.932057544	6.181175429
A_23_P215454	NM_001278939	ELN	5.44144541	6.812392492	6.70258665	8.391272452
A_33_P3274935	NM_030630	HID1	5.440629723	5.789931741	6.012411208	6.398423025
A_22_P00020790	ENST00000514966		5.429200254	4.384903033	5.32309943	4.299210518
A_21_P0011578	ENST00000577449		6.396366199	6.376661099	8.941676876	8.914130511
A_32_P175301	NM_014957	DENND3	4.532197672	4.035179674	5.826208006	5.187283923
A_21_P0014908	ENST00000586348		5.410457203	5.251230487	5.793656929	5.623152861
A_33_P3255914	NM_013262	MYLIP	4.515831394	4.109614598	3.329566115	3.030058546
A_33_P3285639	NM_025244	TSGA10	6.362756726	6.49273811	7.357485165	7.507787329
A_33_P3249897			5.391231389	7.656422305	4.820954365	6.846536508
A_21_P0012933	NR_036494	LOC643201	6.340606812	6.928148592	5.016805544	5.481679483
A_23_P58983	NM_017772	TBC1D22B	4.488255261	4.131570995	4.340608429	3.995657743
A_33_P3422802	NM_025218	ULBP1	4.025720582	4.413845804	3.687047997	4.042521333
A_22_P00013634	ENST00000510967		17.88474024	16.22003952	16.54216519	15.00243053
A_23_P304450	NM_005257	GATA6	3.929888169	3.424239553	3.556647527	3.099022826
A_23_P2705	NM_005767	LPAR6	4.469734645	5.619877468	7.083174434	8.90580215
A_22_P00011952	ENST00000521879		17.86362634	19.4378449	6.037736292	6.569807236

A_33_P3415097	NM_001830	CLCN4	5.335909373	4.589288472	5.557529459	4.779898625
A_33_P3577142	lnc-ANKRD11-5:1	lnc-ANKRD11-5	5.333232661	4.302619613	4.844152243	3.908050853
A_24_P261169	NM_006378	SEMA4D	6.274696956	5.497115484	5.497233863	4.815998223
A_33_P3274397	NM_001145414	CHM	4.457024626	4.400898389	4.643554452	4.58507929
A_32_P54503	NR_024451	JHDM1D-AS1	5.291376508	6.951062208	5.930775464	7.79101414
A_22_P00004878	ENST00000567540		6.219868249	5.680195559	5.69034342	5.196615447
A_33_P3278211	XM_006717970	MMRN2	6.196511298	4.896713385	5.426212694	4.287994817
A_24_P942630	NM_001080424	KDM6B	4.413659001	4.732054302	3.526626108	3.781032074
A_22_P00013430	NM_001731	BTG1	3.662609633	3.589786021	3.421542851	3.353512366
A_23_P149975	NM_031453	FAM107B	3.970774379	3.455593646	4.394850441	3.824648748
A_21_P0013256	ENST00000439784		5.248854082	5.712385445	4.192257462	4.562479759
A_32_P150030	NM_003620	PPM1D	3.96658788	3.632511656	3.734210625	3.41970581
A_23_P119143	NM_003259	ICAM5	5.247100022	5.211486416	4.094651151	4.066859554
A_23_P69497	NM_003278	CLEC3B	4.38951424	4.50571918	4.597375845	4.719083568
A_23_P36825	NM_003979	GPRC5A	4.386345213	4.404950565	3.842831285	3.85913124
A_22_P00014868	ENST00000537961		5.224763632	6.17412418	6.236284629	7.369442607
A_23_P96158	NM_000422	KRT17	4.381133028	4.890743704	5.483747223	6.121613298
A_23_P104073	NM_002960	S100A3	3.945376562	3.623059002	5.072335941	4.657951428
A_22_P00017841	NR_034182	MATN1-AS1	6.118255854	5.136007567	6.189893327	5.196144085
A_33_P3397835	NM_001013734	RFPL4B	5.188062614	4.001674115	4.891140152	3.77265087
A_24_P943472	NM_005126	NR1D2	4.348139301	4.85523089	5.386938159	6.015177239
A_33_P3342111	AK122832	ZNF169	5.136732495	4.684704126	5.404295339	4.928721653
A_22_P00018484	lnc-AC073348.1-1:2	lnc-AC073348.1-1	16.71804466	14.37509782	16.72324551	14.3795698
A_33_P3384835	NR_109874	LOC101927100	5.129976896	4.094870591	4.733644814	3.778508818
A_23_P125686	NR_002226	INGX	16.6333275	12.9456487	16.69759358	12.99566672
A_24_P108311	NM_015277	NEDD4L	3.885981589	3.844991117	3.33651496	3.301320423
A_23_P164966	NM_134444	NLRP4	16.61501182	14.16533851	8.675419472	7.396338614
A_23_P44665	NM_145004	ADAM32	5.981233072	4.82914411	7.567201802	6.109627826
A_33_P3367596	NM_001830	CLCN4	5.973535077	4.944146798	6.251294291	5.174041209
A_33_P3354607	NM_001291470	CCL4L2	3.870954515	4.578213618	3.835511087	4.536294349
A_33_P3514859	NR_125822	LOC101929709	4.278228488	4.617382821	3.316952377	3.579902047
A_23_P50646	NM_001193621	PINLYP	4.276748927	3.978398947	3.966750262	3.690026077
A_23_P216966	NM_000962	PTGS1	4.27074598	4.612208672	4.612844663	4.981659471
A_24_P169048	NR_001450	RFPL3S	5.073276309	7.048966452	4.717128686	6.554123968

A_23_P500861	NM_182961	SYNE1	3.762078082	3.357183344	3.824763774	3.41312247
A_33_P3364646	NM_152545	RASGEF1B	5.92592594	8.336645564	8.237248899	11.58823536
A_23_P430658	NM_014571	HEYL	4.253502019	4.506500995	3.912013209	4.144700376
A_24_P179183	NM_015208	ANKRD12	4.25154069	3.64143345	4.590383486	3.931651416
A_33_P3286953	NM_197941	ADAMTS6	3.833077495	3.301859725	3.629981973	3.126910764
A_22_P00022417	lnc-SFI1-1:2	lnc-SFI1-1	5.034706302	4.445775602	5.515049951	4.869931441
A_23_P70480	NM_003546	HIST1H4L	3.540100341	3.173181084	3.742056606	3.354205275
A_22_P00019588	NM_198581	ZC3H6	4.228508065	3.695156969	3.955647538	3.456712945
A_33_P3324909	NM_001286968	JUND	4.225003138	4.369592439	3.74532656	3.873500228
A_23_P216225	NM_004430	EGR3	4.215540049	5.745832054	5.19357196	7.078901374
A_24_P287473	NM_021818	SAV1	3.7867361	3.33089439	3.780535929	3.325440587
A_33_P3262376	NM_130901	OTUD7A	4.184828596	5.455495202	6.093431429	7.943619473
A_32_P165933	NM_203424	IQCF2	15.7601612	14.27671313	8.148436454	7.381453034
A_23_P336612	NM_173528	C15orf26	5.791804886	4.988652112	23.52919812	20.26639125
A_33_P3613000	NM_001105539	ZBTB10	4.951251715	5.645552719	5.67199419	6.467363016
A_23_P124742	NM_001277	CHKA	3.778192246	3.366623675	3.411203107	3.039611643
A_23_P354027	NM_001002914	KCTD11	3.777093336	3.700686309	3.169630561	3.105511931
A_23_P112159	NM_012154	AGO2	3.772525365	4.551579835	3.871444454	4.670926449
A_22_P00021262	NR_103561	NBPF18P	5.757035711	5.624538927	6.136781276	5.99554474
A_21_P0005617	ENST00000517910		4.160879329	4.288170075	4.27524393	4.406033349
A_23_P74229	NM_032017	STK40	3.679725627	3.599748356	3.735186017	3.654003338
A_33_P3728698	ENST00000517910		4.157684818	4.142547753	4.345269902	4.329449888
A_33_P3214096	NM_001040619	ATF3	4.151184723	4.764015012	3.652491489	4.191700789
A_22_P00020016	ENST00000538329		4.914057732	4.615578928	8.828675039	8.292423225
A_23_P108501	NM_004438	EPHA4	4.14899552	4.276730852	3.905050147	4.025275122
A_22_P00009283	ENST00000449713		5.697623566	4.696992505	6.572902973	5.418553129
A_23_P111724	NM_138290	RUNDC3B	15.34753614	11.50797686	15.06097874	11.29310876
A_21_P0014571	lnc-NT5DC3-1:1	lnc-NT5DC3-1	5.696649704	5.092425694	5.459298729	4.880249719
A_23_P213319	NM_197941	ADAMTS6	3.728229564	3.395491296	3.921911162	3.571887135
A_19_P00318803	NR_120512	SEPT7-AS1	5.656873288	6.263614247	4.883568949	5.407367372
A_22_P00000845	ENST00000514258	CRHBP	5.639809098	11.47538718	6.925366269	14.09112577
A_21_P0014224	XR_242480	LOC101929759	4.822151373	4.308016355	4.471763966	3.994987052
A_23_P18490	NM_001017405	MAEA	3.697775607	3.48334796	4.019236222	3.786167627
A_33_P3316223	NM_003082	SNAPC1	3.693601954	3.230650761	3.681524699	3.220087253
A_22_P00014386	lnc-SFI1-1:1	lnc-SFI1-1	4.809145487	4.367484549	5.576725822	5.06457206

A_33_P3696837	NR_033882	MLK7-AS1	5.599827785	6.271488655	6.533874877	7.317568279
A_21_P0014715	BC013229		4.793715043	4.201275524	4.393014453	3.850096205
A_23_P157117	NM_182898	CREB5	3.661188133	3.347425659	3.262074547	2.982515961
A_22_P00006287	NR_027252	CYP1B1-AS1	4.758446568	3.932355606	4.840499434	4.000163669
A_23_P90172	NM_014330	PPP1R15A	3.386685684	3.496356028	3.21227244	3.316294796
A_23_P114903	NM_002155	HSPA6	4.016845071	28.96607692	4.420802372	31.87907408
A_23_P300150	NM_172387	NFATC1	3.644340442	3.295268058	3.539296509	3.200285736
A_33_P3355252	ENST00000379526		3.995888036	5.954553176	3.69102495	5.500255297
A_33_P3231252	NM_005599	NHLH2	4.701833521	4.62258021	4.95320266	4.869712314
A_23_P115375	NM_001123375	HIST2H3D	3.36046327	2.915599853	3.122744835	2.709350959
A_24_P261259	NM_004566	PFKFB3	3.621267311	3.775859193	3.337129413	3.47959145
A_24_P68631	NM_175065	HIST2H2AB	3.347562905	2.929790899	3.468214648	3.035385443
A_23_P92161	NM_025047	ARL14	3.955162541	3.477750458	5.681781245	4.995955821
A_22_P00002916	NR_038848	LINC01021	5.40453827	6.833013929	6.479789024	8.192464637
A_23_P32707	NM_012291	ESPL1	3.579696547	3.279858064	3.821625524	3.50152286
A_23_P356616	NM_145804	ABTB2	3.574974248	3.419104013	3.522673583	3.369083677
A_33_P3361707	NM_002392	MDM2	3.572671925	4.479688809	4.334438593	5.434850013
A_22_P00014804	ENST00000583460		5.345984442	4.776281893	6.581111858	5.879786173
A_23_P121011	NM_033027	CSRNP1	3.557058067	3.540615816	2.996486324	2.982635276
A_23_P49643	NM_006613	GRAP	13.8653977	15.31695969	7.233793473	7.991095921
A_23_P130149	NM_001976	ENO3	3.909767279	4.20196052	4.238344067	4.555093224
A_23_P167017	NM_022135	POPDC2	3.907836294	5.308858169	4.989578787	6.778422664
A_33_P3212630	NM_018088	FAM90A1	5.324384914	5.495311984	4.595513057	4.743041381
A_22_P00002200	XR_246468	LOC101928666	4.583017041	3.961743475	4.646907431	4.016972887
A_19_P00322276	NR_027105	LINC01091	4.571103271	4.82142746	3.977908393	4.195747859
A_24_P238499	NM_001012716	TYMSOS	5.295216202	4.402562118	5.678192121	4.720976931
A_23_P106194	NM_005252	FOS	3.457982158	4.982695927	3.819268191	5.503282317
A_33_P3359017	NM_001082	CYP4F2	13.6405811	13.37044358	13.60485076	13.33542084
A_23_P207319	NM_003954	MAP3K14	3.526676703	3.205374207	3.972019355	3.610143334
A_23_P404494	NM_002185	IL7R	3.525065708	3.430875588	3.065405982	2.983498018
A_19_P00318954	ENST00000580948		4.549186687	5.8886159	3.814681783	4.937848751
A_24_P157926	NM_006290	TNFAIP3	3.273770833	3.425351423	2.937293076	3.073294232
A_23_P103110	NM_012323	MAFF	3.447177432	3.199976222	3.390969371	3.147798908
A_21_P0014605			13.581224	12.51761863	5.178699741	4.773132992
A_21_P0014188	BC087859	LOC401317	3.866118843	3.324766314	4.031468677	3.46696307

A_22_P00000134	ENST00000485020		5.252821105	6.844346912	5.535022791	7.212051466
A_21_P0014201	ENST00000447430		4.527258255	6.282455926	4.524086734	6.278054821
A_23_P207564	NM_001291470	CCL4L2	3.510482327	4.145866046	3.651645205	4.312578859
A_23_P14184	NM_018676	THSD1	3.49985383	3.798699969	2.983501623	3.238257388
A_23_P56938	NM_002908	REL	3.841045688	3.613423879	4.294186129	4.039711048
A_33_P3299416	NM_181711	GRASP	4.503561599	4.829587064	5.398854128	5.789692331
A_22_P00008748	lnc-KIAA1967-2:3	lnc-KIAA1967-2	13.37764441	15.15017204	19.46752059	22.04695215
A_23_P428129	NM_000076	CDKN1C	3.833019616	4.292009405	4.651726818	5.208753739
A_33_P3352213	THC2700874		5.198665408	6.10734246	5.961476762	7.003485952
A_33_P3335915	AL713682	SYNE1	4.490087174	4.299350489	4.06631723	3.893582083
A_24_P22976	NM_001025604	ARRDC2	3.828478861	3.797514906	3.356222394	3.329077953
A_33_P3391005	NM_001144967	NEDD4L	3.828010624	3.677947079	3.45662572	3.321120999
A_33_P3270429	NR_110524	NR1D2	5.162617183	6.382067577	5.101554727	6.306581693
A_33_P3371509	NR_109967	LL22NC03-75H12.2	13.04593509	13.84191619	13.1800315	13.98419432
A_21_P0007481	NR_033878	LINC00944	3.780548929	3.851554763	3.995525905	4.070569413
A_21_P0011898	NM_207366	SEPT14	3.776610232	3.79687136	4.014437533	4.035974581
A_23_P206140	NM_018602	DNAJA4	3.4379755	8.153034674	3.346306515	7.935644988
A_33_P3619171	NM_021127	PMAIP1	3.366080111	4.713967272	3.156176278	4.420011167
A_23_P61674	NM_020666	CLK4	3.767792425	3.404289234	4.657264328	4.207948057
A_24_P186608	NR_003561	DPY19L2P2	5.030590803	5.752611592	5.760828397	6.587657298
A_23_P110571	NM_001164664	MAST4	3.726334986	3.812715717	3.494069057	3.575065597
A_23_P214139	NM_002912	REV3L	3.400117993	4.247373089	3.269240028	4.083882425
A_21_P0014465	XR_245039	LOC101928173	3.394065089	3.701650668	3.307090402	3.606793941
A_32_P214665	NM_001102566	PCP4L1	5.008839318	5.193444374	6.866341437	7.119406322
A_23_P28582	ENST00000442128		4.330857832	4.066940986	4.773252226	4.482376441
A_23_P74290	NM_052942	GBP5	3.705918796	4.736774225	3.368704165	4.30575842
A_22_P00017934	lnc-ZFP90-2:1	lnc-ZFP90-2	12.50869853	13.17259514	4.840872053	5.097800346
A_33_P3395081	NM_017888	ACSM5	4.984321502	6.085595705	4.533802757	5.535535895
A_23_P138760	NM_013246	CLCF1	3.378314826	3.858293042	3.386803822	3.867988122
A_23_P131208	NM_006186	NR4A2	4.301687437	5.027776232	4.616950928	5.396253558
A_23_P26386	NM_016140	TPPP3	4.296571296	4.195457404	4.44261593	4.338065079
A_19_P00319537	NR_027105	LINC01091	4.291929418	4.056620346	4.436934574	4.193675458
A_33_P3294277	NM_000896	CYP4F3	12.29543803	15.1391113	12.27540035	15.11443933
A_22_P00003637	lnc-CD59-1:1	lnc-CD59-1	4.283718254	5.002068691	5.127672994	5.987548905

A_23_P156788	NM_003764	STX11	4.266256164	4.219620419	5.984127287	5.91871297
A_23_P34345	NM_001078	VCAM1	3.336590567	3.961669895	4.899218683	5.81704191
A_21_P0014177	NR_110054	LOC101926963	4.2387248	4.489488672	6.543812548	6.930945906
A_19_P00803850	NR_040025	LINC01539	4.236157612	4.369989616	4.49043838	4.632303821
A_21_P0010717	NR_027337	HIST2H2BA	4.872088067	6.367063906	4.987344953	6.51768679
A_23_P212792	NM_025009	CEP135	3.631031724	3.66329994	3.292178439	3.321435337
A_24_P174613	NM_033632	FBXW7	3.298950137	3.569071652	3.265617628	3.533009841
A_33_P3404588	NM_139241	FGD4	4.193276666	4.311754215	4.994273404	5.135382451
A_33_P7574262	NR_040030	CFLAR-AS1	4.191880097	4.274217746	4.378526553	4.464530344
A_33_P3400763	NM_001080400	PLIN4	11.81310105	15.86636523	7.445152294	9.999703289
A_23_P86195	NM_152369	SLC44A3	4.179110706	4.271478251	4.878159822	4.98597789
A_23_P142574	NM_058165	MOGAT1	4.175420372	4.32918816	3.988178208	4.135050448
A_22_P00014869	lnc-SLC6A12-2:1	lnc-SLC6A12-2	4.791286897	5.921293218	4.687234627	5.792700625
A_23_P145238	NM_080593	HIST1H2BK	3.05370875	2.72507879	3.706398783	3.307528496
A_23_P133814	NM_003531	HIST1H3C	3.036259979	2.734136535	2.982854268	2.686044966
A_33_P3389728	NM_205860	NR5A2	4.703673803	6.501138773	6.008365361	8.304406012
A_23_P501754	NM_000759	G-CSF(CSF3)	4.674397062	5.751415685	5.333600346	6.562504699
A_23_P144476	NM_199327	SPRY1	4.671801356	4.98522086	5.510612685	5.880305949
A_21_P0001902	ENST00000455309	LOC101928173	3.207748302	3.531854994	3.192959802	3.515572284
A_21_P0004770	ENST00000445974		3.496107832	3.965045467	4.150926729	4.707696102
A_23_P47322	NM_017589	BTG4	4.047384721	4.730933461	4.770631252	5.576326587
A_23_P132760	NM_007117	TRH	11.11079218	14.03145579	4.870056091	6.150234441
A_23_P61398	NM_001001852	PIM3	2.978523912	2.836802944	2.904451308	2.766254784
A_22_P00005132	NR_033878	LINC00944	3.466078329	3.714778991	3.611011459	3.870111471
A_33_P3409124	NM_005126	NR1D2	4.011708229	4.779015094	6.256309894	7.452934689
A_23_P44674	NM_001311	CRIP1	3.1765478	3.118650485	3.738164917	3.670031294
A_33_P3332175	ENST00000451170		0.285106691	0.183890196	0.253298082	0.163374048
A_33_P3213665	NR_038385	LOC728084	0.095397872	0.077187175	0.109589192	0.088669484
A_23_P338952	NM_022753	S100PBP	0.282869209	0.286306302	0.277734232	0.281108932
A_33_P3254380	NM_001257291	SLC9A7	0.242427241	0.206201531	0.218784344	0.186091572
A_23_P122052	NM_001008397	GPX8	0.310651034	0.323484549	0.322271893	0.335585485
A_33_P3299000	NM_031952	SPATA9	0.092365059	0.092987038	0.092174326	0.092795021
A_23_P145874	ENST00000411955	SAMD9L	0.310324094	0.279758092	0.28144733	0.253725604
A_23_P85209	NM_000640	IL13RA2	0.238597673	0.24597599	0.24767019	0.255329062
A_24_P721699	AK027222	A1BG-AS1	0.205221377	0.13310714	0.213104883	0.138220403

A_24_P6083	NM_213720	CHCHD10	0.277816052	0.283039491	0.296167295	0.30173577
A_33_P3211956	NM_005873	RGS19	0.237538961	0.295064555	0.211089309	0.262209503
A_24_P279489	NM_001128159	VPS53	0.23686234	0.228303446	0.223701803	0.215618458
A_33_P3333982	NM_022359	PDE4DIP	0.305809926	0.278895651	0.242380881	0.221048984
A_23_P144677	ENST00000231368	LNPEP	0.235596116	0.226065868	0.248060681	0.238026222
A_21_P0011126	NM_138459	NUS1	0.305510659	0.305960662	0.306374027	0.306825303
A_23_P137248	NR_028062	PRKY	0.201950424	0.183568793	0.15773599	0.143378779
A_21_P0000408	NR_003204	SNORD114-11	0.231343376	0.165149443	0.199100062	0.14213186
A_23_P68505	NM_001190826	FAM217B	0.22995118	0.257311102	0.212085661	0.237319918
A_33_P3260175	ENST00000415386		0.196745379	0.152444058	0.187086671	0.144960209
A_23_P206661	NM_000903	NQO1	0.227566917	0.221950052	0.255190232	0.248891561
A_24_P335305	NM_006187	OAS3	0.227006272	0.23502836	0.189447715	0.196142536
A_23_P20743	NM_032342	TMEM246	0.22377204	0.203903706	0.175898327	0.160280618
A_21_P0000411	NR_003207	SNORD114-14	0.223481084	0.164189181	0.18860813	0.138568391
A_22_P00002869	ENST00000568686		0.222255313	0.147572438	0.13999138	0.09295107
A_32_P123514	NM_001114734	PABPC4L	0.080614934	0.080829906	0.093285917	0.093534677
A_33_P3311267	NM_181608	KRTAP19-2	0.188514106	0.198248182	0.182988461	0.192437216
A_23_P113613	NM_022842	CDCP1	0.258871598	0.235472187	0.232156764	0.211172107
A_33_P3422712			0.21889474	0.254857593	0.213490068	0.248564972
A_19_P00804072	NM_001256071	RNF213	0.186068672	0.162507831	0.099734884	0.087106011
A_33_P3224858	NM_001135652	EIF2AK2	0.217442708	0.240417451	0.213956594	0.236562998
A_32_P21255	NM_013309	SLC30A4	0.216475184	0.219766081	0.233806272	0.23736064
A_21_P0000409	NR_003205	SNORD114-12	0.216454714	0.150414226	0.210244992	0.146099095
A_23_P111000	NM_002800	PSMB9	0.284464176	0.305490646	0.258498326	0.277605504
A_33_P3308055	NM_001282958	EIF4E2	0.254686146	0.287350933	0.242663281	0.273786075
A_23_P105794	NM_033255	EPSTI1	0.21554665	0.213327358	0.234916844	0.232498114
A_23_P404821	NM_001080392	KIAA1147	0.182986834	0.177711444	0.2061686	0.200224895
A_33_P3315779	NM_001165136	HERC6	0.182020438	0.139305978	0.120978103	0.092588355
A_33_P3382887	ENST00000590085		0.074679166	0.094035397	0.058065421	0.073115505
A_22_P00023279	ENST00000580242		0.073693959	0.07385921	0.068577067	0.068730844
A_23_P70818	NM_005631	SMO	0.208912618	0.166693867	0.2056665	0.164103751
A_32_P387648	NM_002016	FLG	0.208238371	0.104313305	0.160861233	0.080580571
A_23_P23616	NM_032129	PLEKHN1	0.17652405	0.128736434	0.168821564	0.123119122
A_21_P0000296	NR_002913	SNORD63	0.176487905	0.208725804	0.149958152	0.177350033
A_23_P209200	NM_001238	CCNE1	0.246226237	0.278960227	0.223970848	0.253746147

A_23_P361584	NM_152680	TMEM154	0.175750576	0.124205699	0.152728565	0.107935681
A_33_P3260342	NM_001005388	NFASC	0.173223736	0.19197805	0.142556431	0.157990505
A_33_P3402020	NM_032040	CCDC8	0.171528866	0.154396795	0.129721987	0.116765531
A_23_P362659	NM_002468	MYD88	0.240641001	0.250826569	0.23888935	0.249000776
A_21_P0001634	XR_425104	LOC102723817	0.068510131	0.069054464	0.091002148	0.091725187
A_23_P51487	NM_018284	GBP3	0.238439621	0.202041865	0.22662332	0.19202932
A_23_P25069	ENST00000621609	OVOS2	0.066851679	0.066514289	0.079913482	0.079510172
A_24_P205994	NM_001270989	EPGN	0.066637898	0.049890346	0.076364369	0.057172344
A_33_P3333455	NM_007046	EMILIN1	0.271153231	0.284159708	0.307221161	0.321957717
A_32_P56249	NR_038996	USP30-AS1	0.166146242	0.095994136	0.134529643	0.077727047
A_33_P3240328	NM_002653	PITX1	0.234189052	0.235650119	0.24008285	0.241580687
A_33_P3251492	NR_026913	LOC100133669	0.165181831	0.1024029	0.189058347	0.117204918
A_33_P3271651	NM_002121	HLA-DPB1	0.195455155	0.158050823	0.181019232	0.146377508
A_23_P34233	NM_014298	QPRT	0.165029361	0.100954858	0.160436098	0.098144981
A_33_P3250750	CU677518		0.164445183	0.176873133	0.160780801	0.172931816
A_23_P349566	NM_001080433	CCDC85A	0.164194123	0.087418858	0.192870458	0.102686471
A_23_P148410	NM_031894	FTHL17	0.266589945	0.3182762	0.266970147	0.318730116
A_22_P00006598	XR_427815	LOC101927675	0.192104067	0.208020773	0.197764282	0.214149963
A_33_P3263232	ENST00000291592	LRRC3	0.062559748	0.147458734	0.053001695	0.124929577
A_32_P76035	NM_033109	PNPT1	0.190685012	0.170607249	0.203760764	0.182306218
A_24_P406714	NM_004641	MLLT10	0.160365246	0.176564379	0.198984418	0.219084627
A_22_P00002230	NR_120643	TMEM26-AS1	0.189535061	0.191271528	0.197970891	0.199784644
A_23_P47728	NM_033063	MAP6	0.158497605	0.101720513	0.166260636	0.106702667
A_33_P3381771	NM_024052	GID4	0.225213178	0.253925039	0.183758964	0.207185932
A_23_P69810	NM_032717	AGPAT9	0.186682607	0.206798393	0.206019453	0.22821886
A_23_P56347	NM_021016	PSG3	0.155717389	0.07368544	0.144531311	0.06839219
A_21_P0008404	NR_110546	LOC102723354	0.154746713	0.152093279	0.150352577	0.147774488
A_24_P942481	NM_180989	GPR180	0.182745532	0.20278648	0.20267241	0.224898658
A_33_P3416321	NM_012434	SLC17A5	0.219336255	0.207252805	0.168492659	0.159210232
A_33_P3321372	ENST00000276974		0.152969451	0.162438485	0.112964964	0.119957662
A_33_P3241334	ENST00000595404	PSG9	0.057596428	0.057368871	0.051903183	0.05169812
A_21_P0000403	NR_003198	SNORD114-6	0.151862894	0.143161319	0.160056195	0.150885153
A_21_P0000406	NR_003201	SNORD114-9	0.216634007	0.174252063	0.209082177	0.168177661
A_23_P137097	NM_006517	SLC16A2	0.214375999	0.237198744	0.220851731	0.244363891
A_32_P54553	ENST00000454608	USP41	0.176574239	0.200817395	0.134391288	0.15284284

A_33_P3258923	NM_005664	MKRN3	0.054001586	0.043182487	0.058858283	0.047066156
A_33_P3685216	NM_130786	A1BG	0.172868952	0.165720185	0.197276372	0.189118268
A_24_P278299	NM_024701	ASB13	0.172689805	0.1933219	0.19111476	0.213948175
A_23_P258493	NM_005573	LMNB1	0.172542788	0.118466425	0.171595432	0.117815978
A_33_P3399755	ENST00000570199	EPB42	0.171928745	0.224702656	0.166598411	0.217736164
A_23_P386268	NM_178497	C4orf26	0.143164089	0.157831263	0.126949502	0.139955491
A_23_P33759	NM_004753	DHRS3	0.207092694	0.215249047	0.258613866	0.268799383
A_33_P3340639	ENST00000369425	GPAM	0.141616363	0.106291267	0.107883667	0.080972928
A_23_P132669	NM_001010983	GLT8D1	0.233182233	0.257263151	0.276025835	0.304531246
A_23_P164958	NM_032040	CCDC8	0.16887182	0.187622959	0.15070297	0.167436682
A_23_P121441	NM_014893	NLGN4Y	0.050294399	0.049911365	0.050790111	0.050403302
A_23_P364792	NR_045129	TXLNGY	0.049917133	0.055370953	0.059490234	0.065989987
A_23_P157607	NM_018142	INTS10	0.228565325	0.277114012	0.261003942	0.316442791
A_23_P258769	NM_002121	HLA-DPB1	0.164717164	0.167763537	0.156046476	0.158932488
A_33_P3358740	NM_145798	OSBPL7	0.199395959	0.232009021	0.20596945	0.239657668
A_32_P452655	NM_001040078	LGALS9C	0.198190498	0.213728845	0.167046411	0.180143028
A_33_P3289128	NM_001137601	ZBTB42	0.133645683	0.189337882	0.124809045	0.176818882
A_21_P0000996	ENST00000418242	LOC100505940	0.132672167	0.063991132	0.120259088	0.058003991
A_24_P68908	NR_033752	LOC344887	0.160061476	0.245359824	0.126955096	0.194610725
A_21_P0000425	NR_003221	SNORD114-28	0.195067825	0.170707165	0.18185317	0.159142796
A_33_P3216232	NM_004763	ITGB1BP1	0.158823759	0.187278	0.161071089	0.189927952
A_23_P143713	NM_021822	APOBEC3G	0.158590582	0.168815891	0.176316638	0.187684855
A_33_P3403132	NM_005234	NR2F6	0.219538205	0.254218491	0.247220843	0.286274135
A_21_P0000422	NR_003219	SNORD114-26	0.157573112	0.16129477	0.157477546	0.161196947
A_33_P3846177	NM_001478	B4GALNT1	0.156429737	0.229919417	0.141760104	0.208358084
A_23_P253012	NM_017577	GRAMD1C	0.129068529	0.132357592	0.188427459	0.19322917
A_33_P3272189	NM_032718	MFSD9	0.128924112	0.101956235	0.125005995	0.098857695
A_33_P3511265	NM_006475	POSTN	0.21738855	0.256139124	0.274570051	0.323513507
A_21_P0000420	NR_003216	SNORD114-23	0.1900455	0.173195031	0.19106542	0.174124519
A_33_P3262181	NM_001006666	APOBEC3F	0.189390764	0.152064475	0.170170071	0.136631914
A_23_P322	NM_182690	EFNA4	0.12717164	0.146819583	0.193150416	0.222992041
A_33_P3618429	NR_073405	PRKXP1	0.154077445	0.127675185	0.123094522	0.102001405
A_21_P0000418	NR_003214	SNORD114-21	0.188407167	0.154170731	0.181100669	0.148191934
A_33_P3263533	NM_001202435	SCN1A	0.125056659	0.149331795	0.121010329	0.144500019
A_23_P1962	NM_004585	RARRES3	0.185990409	0.177285344	0.24885076	0.237203589

A_23_P257971	NM_001353	AKR1C1	0.211690612	0.231052491	0.30075128	0.328258922
A_23_P65230	NM_032813	TMTC4	0.12275566	0.172137179	0.146063256	0.204820836
A_23_P134113	NM_052831	SLC18B1	0.149268016	0.160777481	0.107757582	0.116066342
A_23_P251421	NM_031942	CDCA7	0.122430072	0.159516511	0.098761001	0.128677621
A_21_P0000402	NR_003197	SNORD114-5	0.146367626	0.10845443	0.143092686	0.106027789
A_33_P3420446	NM_001161528	LRRD1	0.146305046	0.199696528	0.18361336	0.250619863
A_19_P00324470	NR_105059	MIR143HG	0.145924422	0.116261562	0.093538251	0.074524216
A_33_P3417695	NM_001014440	ODF3B	0.142970931	0.096689708	0.159844313	0.108100996
A_24_P85775	NM_001039477	THEMIS2	0.116525634	0.088463834	0.116346038	0.088327488
A_24_P166443	NM_002121	HLA-DPB1	0.175386216	0.190427778	0.179724243	0.195137845
A_23_P29922	NM_003265	TLR3	0.141422124	0.155351006	0.158699531	0.174330091
A_23_P372478	NM_175739	SERPINA9	0.140306333	0.15503664	0.137621488	0.152069922
A_23_P108662	NM_006302	MOGS	0.197471605	0.160521259	0.171091535	0.139077356
A_21_P0000412	NR_003208	SNORD114-15	0.16743081	0.127667383	0.163882836	0.124962023
A_24_P406006	NM_024830	LPCAT1	0.109303506	0.129050109	0.116761406	0.137855342
A_24_P392110	NM_182707	PSG8	0.134129485	0.133142045	0.094045016	0.093352672
A_23_P154488	NM_033109	PNPT1	0.191163058	0.251907198	0.22863048	0.301280301
A_21_P0000419	NR_003215	SNORD114-22	0.13303977	0.138433781	0.1347808	0.1402454
A_23_P167367	NM_153426	PITX2	0.162236378	0.176628926	0.13054933	0.142130811
A_21_P0013565	ENST00000479351		0.161340269	0.150107492	0.165497349	0.153975149
A_24_P170983	NM_194312	ESPNL	0.128966318	0.17672962	0.139187655	0.190736478
A_22_P00001349	NR_015389	LINC00667	0.032103542	0.032160982	0.037360646	0.037427491
A_23_P120002	NM_004510	SP110	0.159872637	0.164394666	0.142855401	0.146896094
A_24_P941912	NM_138287	DTX3L	0.12778644	0.118612146	0.110672213	0.102726617
A_33_P3319870	NM_001191323	GREM1	0.183115662	0.232709903	0.217122159	0.275926569
A_23_P121253	NM_003810	TNFSF10	0.101914727	0.08499336	0.104137172	0.086846802
A_33_P3276062			0.101360519	0.051700725	0.059568315	0.030383872
A_24_P48204	NM_003004	SECTM1	0.156667924	0.145397624	0.148236093	0.137572358
A_23_P370682	NM_138456	BATF2	0.124974091	0.138021503	0.117038089	0.129256975
A_23_P374082	NM_033274	ADAM19	0.18006467	0.225332342	0.168343361	0.210664335
A_23_P69109	NM_021105	PLSCR1	0.154836998	0.16974348	0.15790166	0.173103183
A_23_P97064	NM_018438	FBXO6	0.098622259	0.110938518	0.101802598	0.114516028
A_33_P3343175	NM_001565	CXCL10	0.121855511	0.130857035	0.142922479	0.15348023
A_23_P19987	NM_006547	IGF2BP3	0.120499665	0.143294008	0.166878779	0.198446436
A_33_P3226810	NM_003810	TNFSF10	0.096582364	0.068825549	0.113595918	0.080949575

A_24_P227069	NM_020918	GPAM	0.119876748	0.166292425	0.131695698	0.182687612
A_33_P3398448	NM_032789	PARP10	0.174451021	0.162530604	0.210520805	0.196135702
A_23_P207911	NM_016113	TRPV2	0.17326487	0.168242915	0.169605838	0.164689937
A_23_P152782	NM_005533	IFI35	0.145836121	0.157886974	0.121169553	0.131182137
A_23_P142750	NM_002759	EIF2AK2	0.166787854	0.185767579	0.163023843	0.181575239
A_19_P00322941	ENST00000518014	MIR143HG	0.113096235	0.101142362	0.078860858	0.070525543
A_24_P237511	NM_004681	EIF1AY	0.025597664	0.025637527	0.032425163	0.032475658
A_33_P3252359	NM_203314	BDH1	0.08985776	0.117067037	0.066263767	0.086328692
A_24_P348861	NR_001545	TTTY15	0.025008822	0.024916874	0.022852239	0.022768219
A_33_P3400374	NM_001037335	HELZ2	0.16421359	0.136584299	0.142432353	0.118467802
A_21_P0013996	ENST00000593000	TXLNGY	0.024788529	0.024684987	0.022260052	0.022167071
A_21_P0003931	ENST00000505254	MIR143HG	0.109871043	0.086990971	0.094205419	0.074587632
A_23_P49759	NM_002981	CCL1	0.087003945	0.058114236	0.107044979	0.071500634
A_23_P141624	NM_030967	KRTAP1-1	0.138380073	0.10474658	0.169046089	0.127959173
A_33_P3241661	NM_001287682	LOC388780	0.083606447	0.085919757	0.10746859	0.110442143
A_21_P0011609	XM_005275830	LOC100130520	0.082923714	0.21020795	0.083912683	0.212714944
A_23_P47691	NM_003141	TRIM21	0.104174507	0.14622458	0.137700417	0.193283234
A_22_P00001713	XR_428955	LOC102724448	0.082321126	0.095287287	0.075448128	0.087331742
A_23_P75741	NM_198183	UBE2L6	0.126202518	0.156991474	0.110354752	0.137277413
A_21_P0000015	NM_001146106	PARP9	0.074857679	0.046393613	0.084704075	0.052495991
A_23_P132159	NM_017414	USP18	0.122041394	0.147076627	0.143208848	0.172586313
A_23_P73848	NR_125733	TTTY14	0.01887862	0.019185828	0.020432875	0.020765375
A_23_P36882	NM_006183	NTS	0.072729854	0.091803093	0.1258124	0.158806416
A_23_P62081	NM_003020	SCG5	0.092047792	0.115334319	0.09456808	0.118492197
A_32_P164246	NM_033260	FOXQ1	0.090800882	0.08672453	0.091932512	0.087805357
A_33_P3219591	NM_020954	RNF213	0.117528394	0.126189237	0.090317859	0.096973517
A_33_P3401658	NM_031246	PSG2	0.0704955	0.123609169	0.071820789	0.125932975
A_33_P3271276	NM_001130014	PSG5	0.069278937	0.111900892	0.07639124	0.123388845
A_23_P162171	NM_006500	MCAM	0.087278804	0.089206788	0.076159326	0.077841681
A_24_P197964	NM_014788	TRIM14	0.067379554	0.049773222	0.048729607	0.035996522
A_23_P384044	NM_152495	CNIH3	0.066265798	0.097658829	0.074702235	0.110091978
A_23_P137238	NM_004653	KDM5D	0.083542831	0.042970691	0.133112097	0.068466901
A_23_P75786	NM_016582	SLC15A3	0.083449402	0.063103871	0.066938751	0.050618629
A_23_P25194	NM_003806	HRK	0.064380371	0.109773619	0.066315797	0.113073673
A_33_P3687198	ENST00000257572	HRK	0.062962959	0.02640955	0.061693816	0.025877213

A_33_P3373446	ENST00000453638	LOC647264	0.080643055	0.227923959	0.089160151	0.251996089
A_23_P72737	NM_003641	IFITM1	0.131116949	0.135815416	0.128974007	0.133595683
A_23_P250358	NM_017912	HERC6	0.077515422	0.122731325	0.088045021	0.139402995
A_24_P28722	NM_080657	RSAD2	0.1009299	0.23326594	0.094714517	0.218901149
A_23_P204087	NM_016817	OAS2	0.076033401	0.100034577	0.061227446	0.080554883
A_24_P303091	NM_001565	CXCL10	0.075596476	0.0976277	0.102632611	0.132543028
A_33_P3423941	NM_003641	IFITM1	0.119727607	0.126031568	0.135151877	0.142267965
A_24_P274270	NM_139266	STAT1	0.095111908	0.10232024	0.104805184	0.112748149
A_24_P296808	NM_018215	PNMAL1	0.065224538	0.044649866	0.050526574	0.034588283
A_23_P7313	NM_001040058	SPP1	0.085271355	0.142386216	0.101617997	0.16968186
A_23_P327140	NM_001256071	RNF213	0.047358834	0.048285145	0.047332597	0.048258395
A_33_P3391316	ENST00000594169		0.06262358	0.119219725	0.064321829	0.12245277
A_23_P27306	NM_130386	COLEC12	0.062093203	0.050232764	0.064443636	0.05213424
A_23_P216655	NM_014788	TRIM14	0.039601442	0.024491136	0.042229198	0.026116247
A_21_P0001111	ENST00000440492		0.039188878	0.06133518	0.033570444	0.052541673
A_33_P3225522	NM_001032731	OAS2	0.071572423	0.096271857	0.077504128	0.104250577
A_23_P69383	NM_031458	PARP9	0.070468307	0.079424878	0.083815028	0.094467976
A_23_P6263	NM_002463	MX2	0.085308638	0.134297738	0.087797165	0.13821532
A_33_P3225512	NM_002535	OAS2	0.049489324	0.080756428	0.044137476	0.072023309
A_23_P155755	NM_002993	CXCL6	0.049371382	0.053114008	0.069675523	0.074957317
A_23_P30243	NM_022350	ERAP2	0.064131047	0.074630202	0.067833465	0.078938759
A_23_P14174	NM_006573	TNFSF13B	0.045738426	0.055872222	0.063182831	0.077181605
A_33_P3388061	NM_181617	KRTAP21-2	0.045001458	0.162696783	0.052529489	0.189913377
A_33_P3372910	NM_014314	DDX58	0.060905064	0.062462995	0.067997676	0.069737034
A_24_P295543	NM_001001342	BLOC1S2	0.072597705	0.069522823	0.084239183	0.080671226
A_23_P166797	NM_022147	RTP4	0.056155938	0.078954264	0.045334001	0.063738811
A_33_P3258346	NM_017523	XAF1	0.036405424	0.038452912	0.04327374	0.045707511
A_23_P82775	NM_022454	SOX17	0.025347342	0.051180277	0.027835758	0.05620478
A_33_P3217700	NM_004654	USP9Y	0.017893568	0.010588848	0.018430527	0.010906603
A_23_P46781	NM_003638	ITGA8	0.017684002	0.017699574	0.022562152	0.022582019
A_24_P557479	NM_017523	XAF1	0.034496004	0.031967042	0.033917078	0.031430558
A_33_P3220911	NM_004335	BST2	0.032964053	0.029403973	0.030018932	0.026776922
A_23_P64828	NM_002534	OAS1	0.020886792	0.029985707	0.021592336	0.030998607
A_23_P17663	NM_002462	MX1	0.040595622	0.044680406	0.034345063	0.037800908
A_24_P942743	NM_003411	ZFY	0.013494353	0.013550087	0.026065719	0.026173376

A_21_P0006594	NR_001545	TTTY15	0.012018868	0.012057336	0.009813087	0.009844495
A_23_P39465	NM_004335	BST2	0.026684174	0.028278752	0.031118455	0.032978015
A_33_P3401826	NM_207315	CMPK2	0.01716061	0.020308669	0.013326205	0.015770854
A_23_P201459	NM_022873	IFI6	0.031502001	0.028797742	0.024024135	0.021961807
A_23_P112026	NM_002164	IDO1	0.008307861	0.010932447	0.010405952	0.013693358
A_23_P45871	NM_006820	IFI44L	0.010597138	0.01267829	0.011028189	0.013193994
A_33_P3224331	NM_004660	DDX3Y	0.002377843	0.00238584	0.002129222	0.002136383
A_24_P270460	NM_005532	IFI27	0.00595607	0.004622533	0.006719304	0.005214882
A_23_P324384	NM_001039567	RPS4Y2	0.000664061	0.000668152	0.000871728	0.000877099
A_23_P259314	NM_001008	RPS4Y1	0.000569549	0.000574406	0.000739016	0.000745318
